# Risk of SARS-CoV-2 transmission in the close contacts in a small rural area in the Veneto Region (NE-Italy): past evidence for future scenarios

**DOI:** 10.3389/fpubh.2023.1223109

**Published:** 2023-09-05

**Authors:** Marco Bassanello, Ruggero Geppini, Erminio Bonsembiante, Ugo Coli, Aldo Farencena, Maurizio D’Aquino, Andrea Gambaro, Alessandra Buja, Tatjana Baldovin

**Affiliations:** ^1^Emergency and Health Department, Monastier di Treviso Hospital, Treviso, Italy; ^2^Hygiene and Public Health Unit, Department of Cardiac, Thoracic and Vascular Sciences, School of Medicine and Surgery, University of Padua, Padua, Italy; ^3^Hygiene and Preventive Medicine Service Ulss 2 Marca Trevigiana, Treviso, Italy; ^4^Health Department, Monastier di Treviso Hospital, Treviso, Italy; ^5^Laboratory and Microbiology Monastier di Treviso Hospital, Treviso, Italy; ^6^Medical Department Monastier di Treviso Hospital, Treviso, Italy; ^7^Department of Environmental Sciences, Informatics and Statistics (DAIS), Ca’ Foscari University of Venice, Venice, Italy

**Keywords:** SARS-CoV-2, COVID-19, immunity, risk for transmission, social contacts, non-pharmaceutical interventions

## Abstract

**Background:**

During the first pandemic phase of COVID-19, an epidemiological study, named First survey, was conducted on the population of a small rural area in northern Italy. In spring 2020, the results showed how a prolonged lockdown slowed down the spread of the virus.

**Methods:**

After contacting positive First Survey subjects and their families, those who decided to join voluntarily underwent a blood test to assess the presence of qualitative lgG about 2 months after the previous one. This was to determine if IgG persisted in individuals who tested positive in the First Survey as well as to assess the antibody status of their close family members, to determine if they were unintentionally infected.

**Results:**

Based on serological analysis, 35.1% of the samples contained blood IgG. In subjects who tested positive during the First Survey, 62.5% remained IgG positive more than 2 months later. Among family members who were exposed to a positive relative, 23.7% were infected. Linear regression analysis showed that the presence of an infected person within a household resulted in the infection spreading to the others, but not excessively. Induced isolation extinguished the infection regardless of the extent of the contagion (intra-family or extra-family). Micro-outbreaks of SARS-Cov-2 infection which arose in the same household from extra-familial infections played a decisive role on the statistical significance of IgG-positive subjects (*p* < 0.001).

**Discussion:**

The study reveal 52.6% of the IgG-positive subjects in the Second Survey came from the First Survey and 47.4% were family members previously in contact with positive subjects. Data suggest that there have been undiagnosed patients feeding the spread of the virus since the beginning of the pandemic. In conclusion, for future pandemics, it will be necessary: i) to ensure the rapid isolation of symptomatic patients and the early identification of their close contacts, ii) to carry out the maximum number of tests in the shortest possible time, both on symptomatic and asymptomatic subjects, and iii) to implement information campaigns to make people aware of their risks, and implement clear, non-conflicting communication.

## 1. Introduction

### 1.1. Clinical, epidemiological and immunological features of SARS-CoV-2

Coronavirus-2 (SARS-CoV-2), the virus responsible for Coronavirus disease 2019 (COVID-19), emerged and spread worldwide and caused a health crisis that had never been seen before, as neither vaccines nor effective pharmaceutical treatments were available at the time (1). On March 11, 2020, the World Health Organization (WHO) declared the disease a pandemic after it was first described in China in Wuhan in December 2019 (2–4). A clear mechanism of infection transmission was discovered early on: aerosols, or microscopic respiratory particles suspended in the air, or droplets, larger respiratory particles falling within 2 meters of the source (5); this can also happen with asymptomatic people or before symptoms appear. Contact with fomites, inanimate objects, or surfaces infected with the virus, is another method of transmission (6–8). Due to these factors, contagion could occur both near and far, with the risk of transmission in the near field for a person close to an infected person far greater than the risk of transmission from afar (9), as in the case of cohabitants. SARS-CoV-2 was originally transmitted from animals to humans, which characterized it as a zoonosis. Since then, human-to-human transmission events have occurred (8), resulting in symptoms ranging from mild fevers, coughing, dyspnea, cytokine storms, respiratory failure, and death (10–15). An individual’s immune system and the SARS-CoV-2 virus closely interact to create COVID-19 disease. A crucial role for the immune system is thought to play in determining the severity of COVID-19. Cells of the lower respiratory system are infected by the SARS-CoV-2 virus, causing a rapid immune response so powerful which damages them (16–20). Using immunoassays, you can determine both active viral infections and past exposures. To date, several companies and research institutions have developed serological tests for detecting antibodies against SARS-CoV-2 in serum or plasma samples. Coronavirus serological tests primarily target spike protein (S), the most commonly exposed protein, and nucleocapsid protein (N), an abundantly expressed protein during infections. Serologic data can complement the results of RT-qPCR and contribute to seroepidemiology characterization (21). Induction of neutralizing antibodies has been the focus of most correlative studies on immune protection against SARS-CoV-2 (22–25). Despite this, in patients with less severe cases of COVID-19, antibody responses are not always detectable (26–28). Additionally, SARS infections tend to induce short-lived B-cell memory responses (29, 30). As a contrast, T-cell memories can persist for several decades (30–32).

### 1.2. Public health response: the Italian experience

Global health systems have suffered immeasurable pressures and disastrous consequences due to rapid pandemic spread and unprepared public policies to counteract it (33, 34).

During the early stages of the pandemic, Italy was one of the most affected countries after China. On February 21, 2020, the Italian National Health Service reported two hot-spots of COVID-19 cases in northern Italy: Vo’ Euganeo (Padua), Veneto region, and Codogno (Lodi), Lombardy region (35–38). Viral spread in the two regions was controlled using different strategies. Lombardy only investigated symptomatic cases, while Veneto tested both symptomatic and asymptomatic individuals. A different strategy resulted in a different impact of infection: in Lombardy, COVID-19 cases increased rapidly, and many patients developed severe forms (38).

Two red zones were set up by the Italian government on February 24 to contain the outbreak, with quarantined areas, severe mobility restrictions and temporary closings of schools and stores (36, 39, 40). Supermarkets and pharmacies with a distance of at least 1 meter between customers were the only businesses that could remain open (37). The epidemic spread rapidly throughout the country but mostly in northern Italy in the weeks that followed. On March 8, 2020, the above extraordinary measures were extended to the entire Lombardy region and neighboring provinces. After almost 100 percent of the total deaths from Covid-19 increased in the 48 h leading up to the decree on 11 March, Italy was put under lockdown. This makes Italy the second most infected country in the world after China (36, 37, 41). Since 31 March, the number of newly reported Covid-19 cases in Italy has stabilized after steadily increasing for almost 4 weeks (36). After expiration on 3 April, a restrictions decree was extended until 18 May (37, 41). Italian authorities initiated Phase 2 following 69 days of lockdown, known as “Coexistence with the virus” (42). Infections, which exceeded 6,000 at the end of March, began to decline at the end of May with daily increases of fewer than 500 (43, 44). As part of the previous epidemiological study, it was observed how much the virus circulated in a small rural Italian community during the spring of 2020, and how the infection developed after a prolonged lockdown, when the situation improved, and limitations had been reduced (45). Only 0.2% of the population tested positive for NAAT by nasopharyngeal swab during the first Phase of the epidemic, according with the studies carried out during the same phase in Vò Euganeo, where a reduction in infections from 2.6 to 0.3% was found following the application of restrictions (38). A random sampling of the general population was used to test for anti-SARS-CoV-2 immunoglobulin levels during phase 2, which showed 97.9% of respondents were negative, while 2.1% had mildly symptomatic or asymptomatic infection resulting from distantly positive IgG (45).

### 1.3. Aim of the study

Two months later, almost every subject (16 out of 19) found to be IgG positive against SARS-CoV-2 in the previous study by Bassanello and colleagues (referred to as the First Survey) (45) was retested by the same method, as were their family members (referred to as the Second Survey).

This study seeks to evaluate the risks of infection in the close contacts based on the intervening familial relationship and to retrospectively analyze the group of people who surrounded positive subjects early in the epidemic. In addition, the seroprevalence of subjects who were found positive in the first study 2–3 months after asymptomatic or pauci-symptomatic infection was observed. Several studies have now shown that anti-SARS-CoV-2 antibodies persist in nonvaccinated subjects for months after infection (46–49). Ex-post considerations will also be made about the effectiveness of restrictions at the beginning of the pandemic when vaccines were not yet available and there were no proven standardized drug therapies.

## 2. Patients and methods

The study was conducted between May 2020, and August 2020, in the Municipality of Monastier di Treviso in the Veneto Region (Northeast Italy). Data were collected in collaboration with Giovanni XXIII Hospital, a private healthcare Center that is part of the National Health Service.

### 2.1. Study design

#### 2.1.1. First Survey

During the first pandemic phase of COVID-19, characterized by the instant lockdown and a subsequent gradual release of personal and social constraints, a study on a complete voluntary basis was conducted about a population of 922 adult subjects (a quarter of the city’s population of about 4,400 people) and representative of one subject for each family (about 1750 families). Recruitment for this investigation began on May 25th, 2020. For the sake of simplicity, we define this investigation as *First Survey*. In addition to venous blood sampling, a complete analysis of the patient was performed, examining symptomatology, exposure to infected individuals, and comorbidities. In 19 subjects (2.1% of the study cohort), IgG antibodies were positive and IgM antibodies tested negative. Serological positivity was significantly correlated with only fever (especially when accompanied by a decrease in taste and smell), quarantined subjects, and some COVID-19 contact subjects (45).

#### 2.1.2. Second Survey

**Figure 1:**
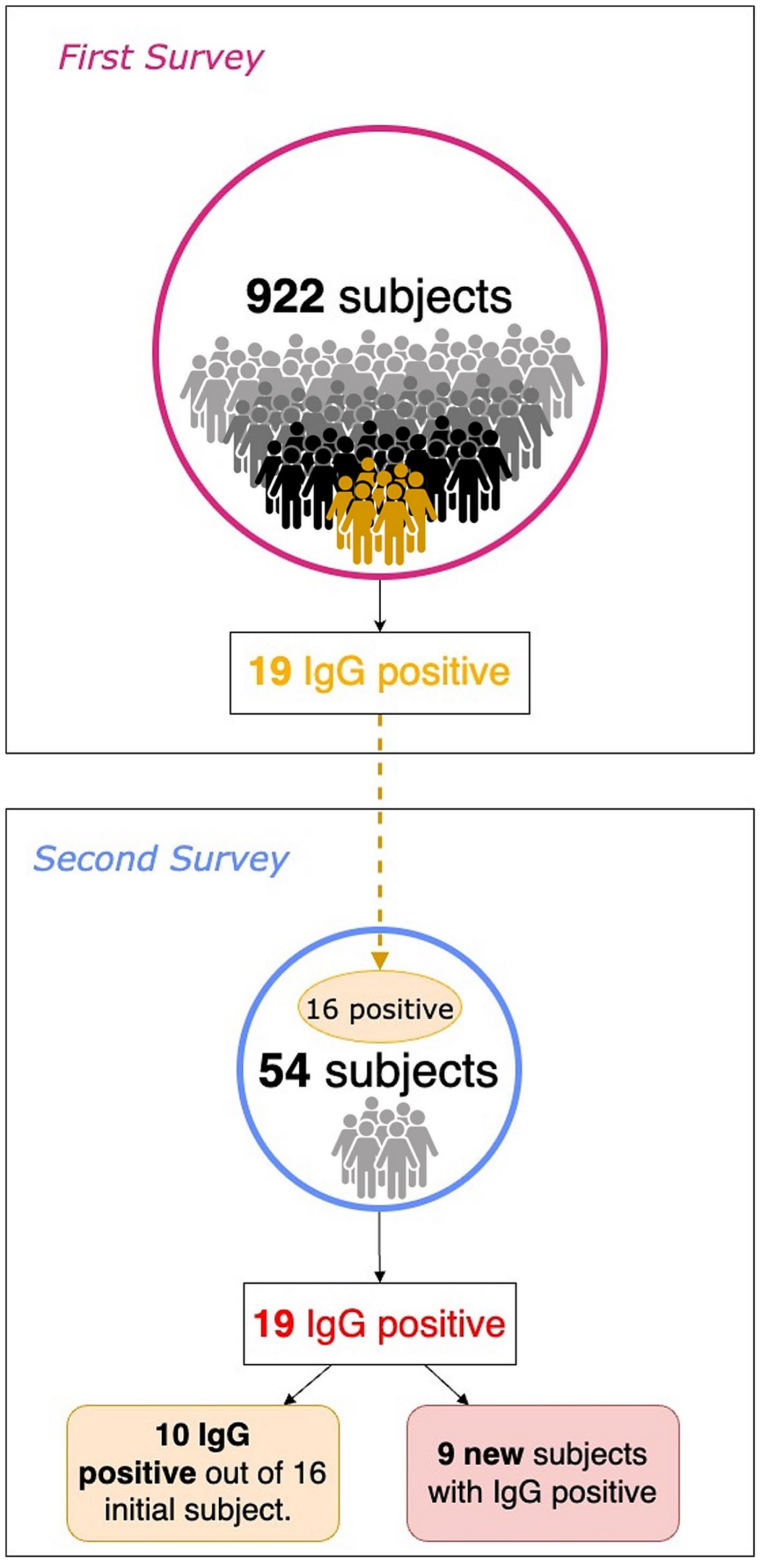
Population comparison between First and Second surveys.

In the summer of 2020, about 60 to 75 days after the First Survey, a subsample of patients with serum IgG positivity was evaluated along with their family members. To simplify, we refer to this new subsequent investigation as the *Second Survey*. Overall, 54 subjects were recruited, including 16 out of 19 positive subjects in the First Survey and 38 from their family networks (Figure 1). In the First Survey, the latter subjects were not included. The aim of the Second Survey is to investigate the living environment of the person who tested positive during the First Survey: the risk of transmission of the infection to family members, based also on the intervening kinship relationship, and the persistence of seroprevalence to anti-SARS-CoV-2 antibodies.

### 2.2. Study protocol and data collection

Peripheral venous blood samples (5 mL) were collected in Serum Separator Tubes (BD Diagnostic Systems, Franklin Lakes, NJ, USA) and centrifuged at room temperature at 1600 rpm for 10 min. Specific qualitative determination of Anti-Nucleocapsid (N) and Anti-Spike (S) IgG and IgM antibodies directed against the SARS-CoV-2 plasma levels were measured by immunochromatographic method and a two-phase immuno-enzyme sandwich method with final fluorescence detection (ELFA).

At the time of sampling, a complete medical history was obtained. Four closed-ended questions were asked by the health professional to the patient in the questionnaire:

1. *Presence of symptoms even in the previous weeks/months* (none, fever, coughing, general malaise, diarrhea, flu symptoms, sore throat, nasal discharge, altered taste or smell);
2. *Relationship in family context* (single, son/daughter, parent, grandfather/grandmother, husband, wife);
3. *Previous quarantine or buffer due to risk factors*;
4. *Contact with infected subjects through social and / or work activities*.

Symptomatology was classified according to priority (none, main symptom and up to 3 secondary symptoms).

Symptom numerosity (none, one, two, three, or four symptoms simultaneously) of each study participant was also collected. Scientific literature that existed during data collection guided the choice of grouping symptoms by number and type. The aim was to assess whether a diagnostic model and/or a main symptomatology could be hypothesized. In the current state of knowledge, this model does not exist. Five groups of symptoms were identified: *absence of symptoms (0)*, *primary symptom (1)*, *1° Secondary symptom (2)*, *2° Secondary symptom (3)* and *3° Secondary symptom (4)*.

Subjects who declared no symptoms were included in the group (0). Symptoms of fever, illness, diarrhea, flu symptoms, sore throat, nasal discharge, alteration of taste or smell, cough, headache, skin rash, dyspnoea, or not detected, could be included in each of groups (1) to (4) in order of importance in the presentation of each symptom.

The study was conducted in accordance with relevant guidelines and regulations, respecting the Privacy of patients as approved by the Ethics Committee of the Giovanni XXIII Hospital (study protocol # 12/2020 of 10 April, 2020). Informed written consent was obtained from all the participants included in the study. For minors, parental consent has been obtained.

### 2.3. Contact patterns and data analysis

For the statistical analysis, age, gender, family relationships, symptoms (additional or single), previous quarantine, contact with infected individuals, and molecular COVID-19 swab outcome were taken into account (Table 1). The same methods previously described were used to test all subjects for anti-SARS-CoV-2 antibodies. Regarding the 16 subjects already positive in the First Survey, the persistence of antibodies over time was considered. In the 38 subjects without previous analysis, the absence of previous exposure to the virus was assessed. Statistical analysis was performed using the Student-*t* test for paired data. Simple regression was used for correlation analysis. Groups were compared using the unpaired Student *t*-test (Tables 2, 3). Statistical significance was set at *p* ≤ 0.05.

**Table 1:** Characteristics of the study group.

	All patients (*n* = 54)
*Age (yr, mean* ± *SD)*	40.5 ± 19.2
*Gender (male/female)*	25/29
*Relationship*	
Single	1 (1.9%)
Son/Daughter	25 (46.3%)
Parent	7 (13.0%)
Grandfather/Grandmother	1 (1.9%)
Husband	9 (16.7%)
Wife	11 (20.4%)
*Absence of symptoms (0)*	21 (38.9%)
None	21 (38.9%)
*Primary symptom (1)*	11 (20.4%)
Fever	23 (42.6%)
Illness	0
Diarrhea	1 (1.9%)
Flu symptoms	4 (7.4%)
Sore throat	0
Nasal discharge	0
Alteration of taste or smell	3 (5.6%)
Cough	0
Headache	1 (1.9%)
Skin rash	1 (1.9%)
Not detected	0
*1° Secondary symptom (2)*	11 (20.4)
Diarrhea	1 (1.9%)
Flu symptoms	2 (3.7%)
Alteration of taste or smell	4 (7.4%)
Cough	3 (5.6%)
Headache	11 (20.4%)
Dyspnea	1 (1.9%)
*2° Secondary symptom (3)*	10 (18.5%)
Illness	1 (1.9%)
Nasal discharge	2 (3.7%)
Alteration of taste or smell	8 (14.8%)
*3° Secondary symptom (4)*	1 (1.9%)
Alteration of taste or smell	1 (1.9%)
*Quarantine*	
Yes	8 (14.8%)
No	46 (85.2%)
Contact with infected subjects	52 (96.3%)
Previous IgG positive in First Survey	16 (29.6%)
Total IgG positive in Second Survey	19 (35.2%)
*Covid-19 NAAT*	
Not performed	47 (87.0%)
Performed	7 (13.0%)

**Table 2:** Characteristics of the subjects grouped by lgG positivity and IgG negativity, and *p*-values of statistical comparison between groups (bold values are significant).

	IgG negativity	IgG positivity	*p*
*Age (yr, mean ± SE)*	37.4 + 19.5	46.3 + 17.7	0.1
*Gender (M/F)*	**21/14**	**4/15**	**<0.005**
*Relationship*			0.1
Single	0	1	
Son/Daughter	19	6	
Parent	2	5	
Grandfather/Grandmother	1	0	
Husband	6	3	
Wife	7	4	
*Absence of symptoms (0) and Primary symptom (1)*			0.2
None	17	4	
Fever	12	11	
Illness	0	1	
Diarrhea	3	1	
Flu symptoms	2	1	
Sore throat	0	1	
Nasal Discharge	1	0	
*1° Secondary symptom (2)*			0.6
Diarrhea	0	1	
Flu Symptoms	1	1	
Alteration of taste or smell	1	3	
Cough	2	1	
Headache	6	5	
Dyspnea	1	0	
*2° Secondary symptom (3)*			0.2
Illness	0	1	
Nasal Discharge	2	0	
Alteration of taste or smell	6	2	
*3° Secondary symptom (4)*			n.d.
Alteration of taste or smell		1	
*Symptoms by numerosity*			**0.017**
0	**17**	**4**	
1	**7**	**4**	
2	**3**	**8**	
3	**8**	**2**	
4	**0**	**1**	
*Quarantine*			**<0.001**
No	**35**	**11**	
Yes	**0**	**8**	
*Contact with infected subjects*			0.3
No	1	0	
Yes	34	18	
Not detected	0	1	
*Contact settings*			
Family infection	**30**	**8**	**<0.001**
Extra-Family infection	**5**	**11**	**<0.002**

**Table 3:** Characteristics of the subjects grouped by lgG positivity and IgG negativity and *p*-values (bold values are significant) in Second Survey.

	IgG negativity	IgG positivity	*p*
*Previous IgG-positive subjects in First Survey*	**6**	**10**	**0.006**
Familial in contact with previously positive subjects in First Survey	**29**	**9**	
*Covid-19 NAAT*			**0.007**
Not performed	**34**	**13**	
Positive NAAT	0	4	
Negative NAAT	1	2	

## 3. Results

Overall, 54 subjects participated in the study, with a mean age of 40.5 ± 19.2 years, 25 males and 29 females among them. Medical history information, shown in Table 1, was collected during the study after patient consent was obtained. This data included: relationship within the family context (single, son/daughter, parent, grandparent/grandmother, uncle/aunt, husband/wife), the number and presence of symptoms, which occurred in the past few months (none, fever, illness, diarrhea, flu symptoms, sore throat, nasal discharge, altered taste or smell, cough, dyspnea), a previous period of quarantine resulting from positivity or cohabitation with positive individuals, and any previous contact with infected individuals.

Each participant’s information was collected. No data missing is present. A descriptive analysis of the sample under study revealed that 46.3% of the subjects were children.

Considering the division of symptoms according to priority, 21 subjects (31.8%) had never experienced symptoms, while 33 had more than one symptom (whose 22 had two or more symptoms). In terms of primary symptom and first secondary symptom, out of the total sample, the most frequent were fever (42.6 percent) and headache (20.4 percent), respectively. The other symptoms occurred less (generally <5–8% each). A total of 52 subjects reported close contact with an infected person, but only 8 of them received a contumacies procedure, i.e., quarantine (14.8%). Only 7 subjects in the study population had a COVID-19 NAAT molecular swab. An overview of study group characteristics and outcomes can be found in Table 1.

**Figure 2:**
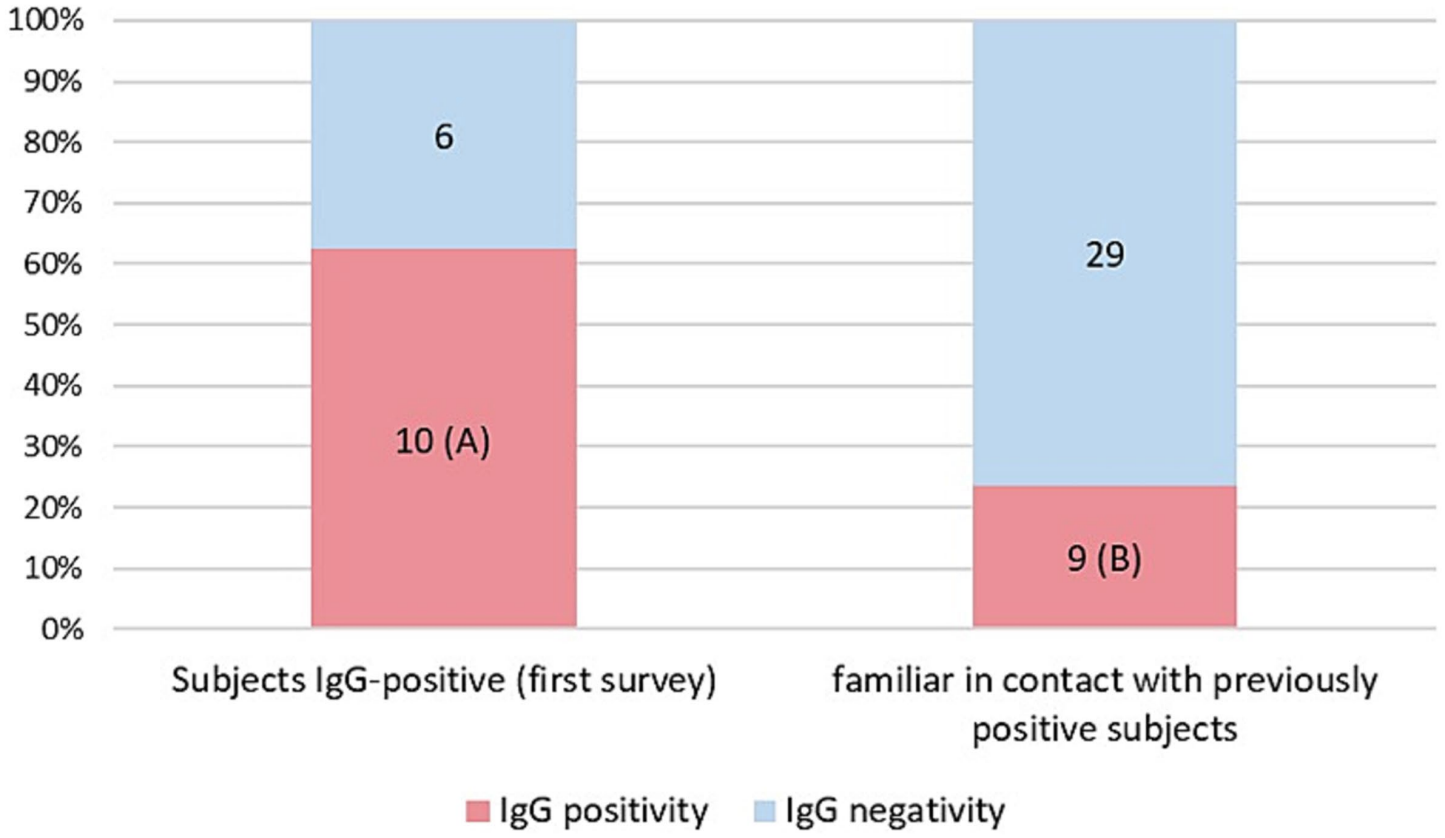
Percentage of IgG positive patients in the two different groups: IgG positive subjects in the first Survey still positive (A) and IgG positive subjects in family members previously in contact with positive subjects (B).

**Figure 3:**
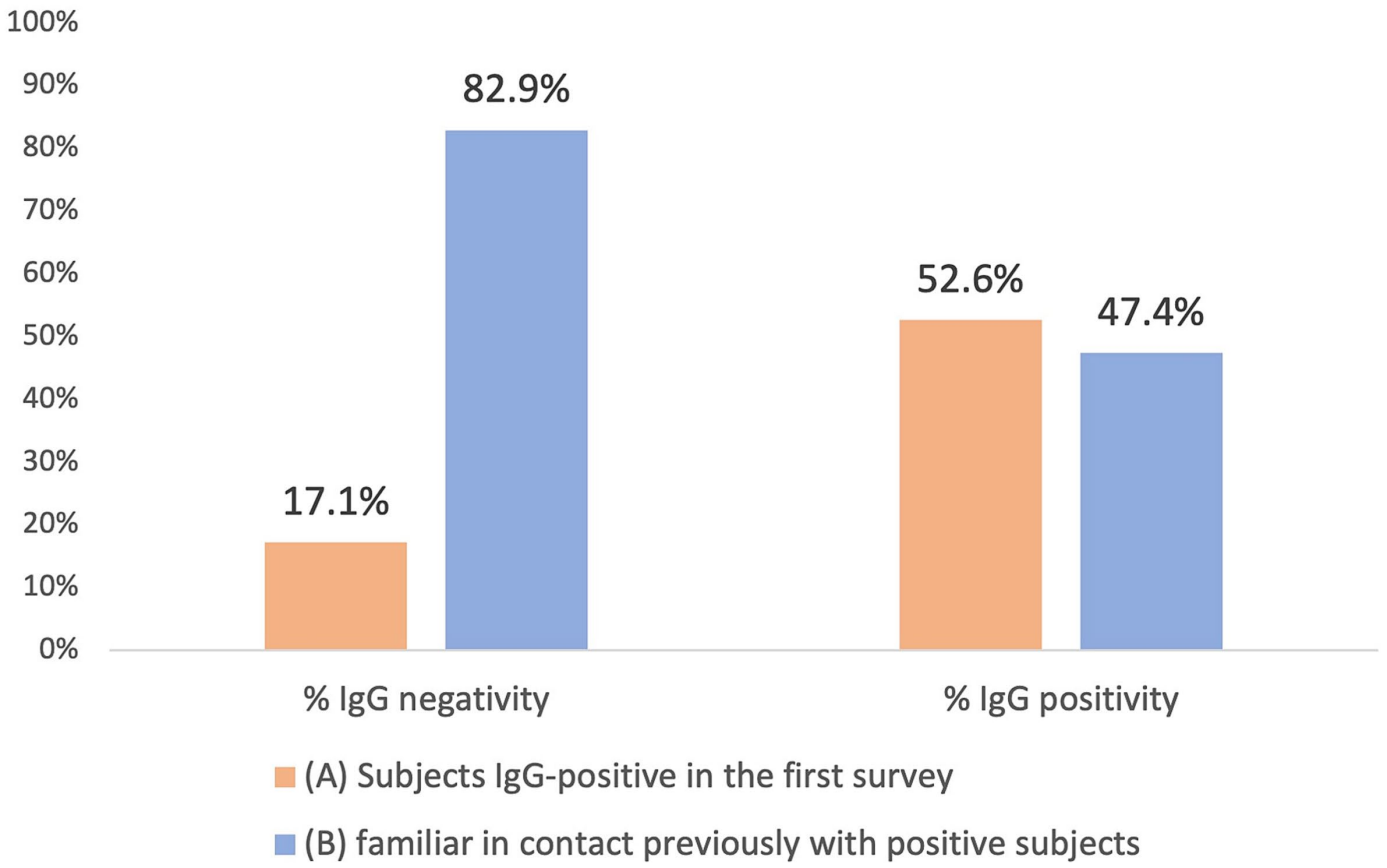
Comparison between IgG positive and IgG negative in the two different groups: IgG positive subjects in the first Survey group (A) and family members previously in contact with positive subject (B).

As a result of processing the venous blood samples of the 54 subjects, 19 samples (35.1%) contained blood IgG (Table 3). Among the 16 subjects who were positive at the First Survey, 10 remained positive, i.e., 62.5% of the subjects had persistence of IgG positivity more than two months later. Of the 38 family members in contact with a positive relative had 9 newly infected relatives in the family context, i.e., only 23.7% became infected following exposure (Figure 2). Analyzing the total number of IgG-positive subjects in the Second Survey, it is observed that 52.6% were positive subjects from the First Survey and 47.4% from family members previously in contact with positive subjects (Figure 3).

In linear regression analysis (Table 2), it was found that stratification by symptom priority, age, and kinship was not statistically significant in predicting IgG positivity. Conversely, the association between IgG positivity and the number of symptoms was statistically significant. Similarly, IgG positivity is also related to gender, quarantine performance, close contact settings (i.e., family or extra-family exposure) and NAAT Covid-19 results.

## 4. Discussion

During Phase 2 of the first pandemic wave of SARS-CoV-2, which struck Italy in the spring of 2020 and affected 2.1% of the population, a cohort study was conducted in a small rural municipality in the Veneto region (north-eastern Italy) (45). Following contact with positive subjects from the First Survey and their families, those who voluntarily participate underwent a blood test to assess the presence of qualitative IgG approximately two months after the initial one. We conducted a study to determine if IgG persisted in individuals who tested positive in the First Survey as well as to assess the antibody status of their close family members, to determine if they were unintentionally infected, developing an undiagnosed asymptomatic or pauci-symptomatic condition.

### 4.1. Gender differences in infection risk and contagiousness

The analysis revealed a statistically significant difference between males and females, suggesting a higher infection risk in females. Initially, there were conflicting hypotheses in the scientific literature regarding gender-based contagiousness during the early stages of the pandemic. Some studies leaned towards male prevalence (50–53), while others toward female prevalence (54–56). However, current systematic reviews and larger population samples have shown that such hypotheses do not hold (57, 58).

### 4.2. Symptomatic patterns and antibody status

The study explored the correlation between IgG positivity and the number of symptoms exhibited by the subjects, revealing a statistically significant association. Fever, a common symptom, was often linked to secondary symptoms, such as headache and/or loss of taste or smell (51, 59, 60). These features were specific to the time under investigation, predating the emergence of less symptomatic viral variants and the development of vaccines that have mitigated symptomatology (60–62). This study also confirmed that the presence of multiple symptoms in the early stages of the pandemic was an important factor in suspecting the presence of the disease, as COVID-19 was the most common, symptomatic, and heterogeneous disease at the time of lockdown (63–66). However, stratification according to symptomatological priority was not significant, refuting both the hypothesis of predictive diagnostic patterns and the recognition of a primary symptomatology (67, 68). Although initially supported by the scientific literature, these hypotheses have now been disproved (59–62). Due to the heterogeneity of symptoms caused by COVID-19 and the emergence of variants constantly changing its characteristics, it was impossible to create a standardized symptom pattern (60–62, 69, 70).

### 4.3. Impact of quarantine and close contact settings

The significant correlation between IgG positivity and quarantine can be attributed to the meticulous and precise *contact tracing* efforts of health workers, especially during the early stages of pandemic. They accurately identified a high risk of infection by carefully examining the detailed descriptions provided by the respondents. Therefore, it was possible to speculated specific thresholds of effectiveness for contact tracing in decision-making (71–73).

When considering close contact settings, it is evident that micro-outbreaks of SARS-CoV-2 infection arising in the same household from extra-familial infections (i.e., no cohabiting relatives, friends, or co-workers) played a decisive role in the statistical significance of IgG-positive subjects (*p* < 0.001). This was most likely due to the mode of virus transmission and less attention paid during phase 2 of the pandemic when infection was believed to occur more easily within intra-family households due to the greater intimacy (74–77). Hence, it is possible that people took greater care within their own households, partly out of concerns over infecting a relative, than with extra-familial contacts considered less at risk (76, 77). According to the current literature, infection risks are high even at several meters and/or when wearing unsuitable personal protective equipment (PPE), like fabric or surgical masks (78). Thus, the presence of an infected person in a household resulted in an inevitable spread of infection among household members, but not with an excessive rate of diffusion (75, 77). Regardless of the mode of infection (intra-family or extra-family), induced isolation still extinguished the infection (79, 80). It was already noted in the previous study and other investigations that quarantine of close contacts resulted in an increased risk of infection (compared to those who had not declared any contact), but at the same time allowed, along with lockdown, he extinguishment and prevention of further spreading of the family outbreak (77, 79, 81). Based on this current analysis, only 23.7% of family members became positive as a result of close contact with family members, which is similar to some studies (76), but nearly 10 percentage points higher than others (56).

### 4.4. Family transmission and persistence of seropositivity

Although contagion occurs within families, there is a greater likelihood of positive outcomes occurring between parents and children, presumably due to the difficulty of isolating children (75, 76, 82). In contrast, grandparents’ negativity indicates that their presence led to greater attention to providing protection to older adult subjects by reason of their frailty (83). SARS-CoV-2 rates of familial secondary infections have also changed significantly as a consequence of vaccination and variants, making it impossible to generate a predictive model based on them (75). The persistence of seropositivity in positive subjects from the First Survey is noteworthy, with 10 subjects (62.5%) remaining positive in the Second Survey. This suggests the presence of anti-SARS-CoV-2 antibodies for at least 2 months after infection (46, 47). Several studies have demonstrated that patients with mild COVID-19 maintain hight antibody titers during the initial 1–2 months (84), but these levels decrease significantly thereafter, affecting 44% of cases (85, 86).

### 4.5. Factors affecting IgG positivity in COVID-19: impact of contact setting and quarantine

Linear regression (Table 2) shows that stratification by symptom priority, age, and kinship is not statistically significant in correlation with IgG positivity. Only two variables show significant associations: contact setting, with extra-familial contacts having a greater likelihood of being positive and having quarantined (87).

In this study, carrying out a quarantine greatly increases the likelihood of being positive. The reason for this is that living with a positive person significantly increases the chances of getting infect, particularly in small living environments and depending on the level of rigor of the quarantine (79).

Despite being statistically significant, the number of symptoms now has a much different clinical and scientific value compared to previous studies. It is important to consider the absence of symptoms, as the lack of symptoms often suggests negative IgG results (88). Although, some asymptomatic positive cases are also present, which, according to many studies, contributed to the pandemic’s early outbreak and resulted in lockdown (70, 89). As the number of symptoms increases, the likelihood of having a positive IgG appears to increase linearly. With the advent of variants and mass vaccination, this linearity has been significantly altered. Currently, having more symptoms has no significant impact on predicting the likelihood of the general population being positive for COVID-19. The number and type of symptoms are not sufficient elements for determining whether COVID-19 is present.

### 4.6. Importance of testing and information campaigns

Data support the speculation that since beginning of pandemic, there has been an undiagnosed group of patients who contributed to the spread of the virus, as also revealed by some mathematical models (73, 81). Accordingly, nearly 60% of the infected individuals were undetected during the same period under analysis (81, 90). In this study, these individuals are mainly represented by family members of those who tested positive, who were typically asymptomatic or had mild symptoms, and therefore did not undergo a swab test. In fact, within this group, only a few people opted for COVID-19 swabs, and almost all of them were family members of the positive subjects in quarantine. Recent studies have evaluated periodic testing as an alternative to quarantine to mitigate the risk of COVID-19 transmission (91). As shown by Romagnani et al., the difference in the evolution of the epidemic in the early stages between the Veneto and Lombardy regions is strongly influenced by the number of subjects tested. Testing both symptomatic and asymptomatic individuals during epidemic peaks has been proven highly effective in curbing the spread of the virus (38). In the healthcare setting, a sequential approach with PCR testing involves adopting an organized and systematic method for conducting tests on both staff and patients. This approach entails administering the tests in a specific order or sequence, ensuring comprehensive and efficient screening. Thanks to this precise methodology, the Giovanni XXIII Hospital in Monastier di Treviso has been able to effectively manage and prevent the spread of Covid-19, maintaining its status as a Covid-free facility (92). Consequently, based on this evidence, several essential measures must be included in countering future pandemics:

- Promptly isolate and test symptomatic patients immediately upon the onset of symptoms using molecular testing.
- Conduct detailed and accurate *contact tracing* to identify close contacts, both within the family context and in extra-familial settings, as these individuals could unknowingly contribute to the virus spread, particularly if asymptomatic.
- Perform molecular testing regularly for close contacts and adjust the test frequency based on the epidemiological trend of the pandemic, which may be influenced by the development of virus variants. Utilize forecasting models to aid in determining appropriate testing intervals (93). Additionally, include serological testing to gain a comprehensive understanding of the actual extent of virus circulation in the target population. By combining molecular and serological testing, a more comprehensive and informed approach to managing the pandemic can be achieved.
- Implement timely information campaigns to raise awareness about the risks associated with the spread of infection disease, ensuring clear and consistent messages. It is crucial to include extra-family contacts in these awareness campaigns, given their role in the spread of the virus, as observed in this study.

Consequently, it is crucial that communication is institutionalized, grounded in scientific data, and initiated from the initial stages of an emergency to ensure its effectiveness. Swift and widespread communication aids in disseminating prevention measures, testing and treatment guidelines, and updates on the evolving situation. Additionally, it serves to combat infodemic and misinformation that can cause confusion and panic among the public. Governments, health authorities, and media outlets play a pivotal role in rapidly and extensively communicating information during emergency situations, thereby keeping the public well-informed and engaged in the response efforts.

## 5. Limitations

The findings pertain to a specific context, namely that of a geographical area in a rural municipality. Some studies have demonstrated that effective strategies for addressing COVID-19 can vary depending on the geographic level of evaluation, such as city, county, or neighborhood (93). Each of these geographic units may have unique characteristics, as population densities, healthcare infrastructure, and socio-economic factors and population behaviors that influence the spread and impact of the virus presenting different challenges and resources in responding to the pandemic. Changing the context, or the geographic level of analysis, can indeed alter the endings. Different geographical units may have different infection rates, vaccination rates, and health outcomes. As a result, strategies and interventions that prove effective at one level might not necessarily be suitable or successful at another level.

In addition to the intrinsic geographic diversities of the analyzed area, the limitations of this study arise from the small sample size analyzed and the data collection taking place during the early and intricate stages of the SARS-CoV-2 pandemic. Nevertheless, early sampling also provides strength as it allows critical examination of the onset of the pandemic, shedding light on the challenges and complexities of managing an unforeseen pandemic. As a result, some actions taken have been one-sided, overlooking the broader context, and underestimating crucial factors, such as the significant role of extra-family contacts in the spread of the virus. On the other hand, efficient management of close contacts has proved to be essential to contain emerging outbreaks within households. Thus, this preliminary study aims to highlight the risk of disease transmission among close relatives and emphasize the possibility that undiagnosed cases could contribute to the spread of future pandemics.

## 6. Conclusion

This study was conducted in 2020, during a time when the pandemic was in its early stages and significantly different from the current phase. Vaccine development and emergence of SARS-CoV-2 variants have substantially altered the trajectory initially predicted. However, several aspects of SARS-CoV-2 infection remain relevant and may serve as warnings for future decisions in managing other air-spread infections, like the one causing COVID-19.

The study reveals that at the beginning of the pandemic, when the NAAT swab was available especially for symptomatic cases, after 2 months almost half of the IgG positive subjects (47.4%) were family members who had been in contact with a positive relative. Therefore, serological analysis of anti-N and anti-S antibodies becomes necessary to assess the actual impact of virus shedding in the general population. Monitoring the serological status of patients at an early stage of the pandemic is of paramount importance because it demonstrates that anti-N antibody-positive individuals remained so even after 2 months from the initial infection. It is equally essential to stress that while the NAAT swab is considered the gold standard for diagnosis, it alone cannot provide a comprehensive understanding of the epidemiological extent of the pandemic. The First Survey suggests implementing measures such as quarantine and, if necessary, lockdown in limited epidemic situations to effectively contain the pandemic. However, it is crucial not to underestimate the importance of close contacts which, if undetected, could fuel a pandemic, and evade public health control. This evidence must be considered to set possible alerts for future infectious diseases outbreaks. To conclude, we must recognize the pivotal role played by contacts in spreading pandemics and contemplate the most effective ways to contain outbreaks based on the specific stage of the epidemic when action is taken. Taking a forward-looking and inclusive approach is imperative, without underestimating anything that could contribute to the ongoing epidemic, whatever its nature.

## Data availability statement

The raw data supporting the conclusions of this article will be made available by the authors, without undue reservation.

## Ethics statement

The studies involving humans were approved by Ethics Committee of the Giovanni XXIII Hospital (study protocol # 12/2020 of 10 April, 2020). The studies were conducted in accordance with the local legislation and institutional requirements. Written informed consent for participation in this study was provided by the participants' legal guardians/next of kin.

## Author contributions

MB: conceptualization, investigation, and writing – original draft preparation. RG: original draft and writing – review & editing. EB: conceptualization and writing – review & editing. UC, MD’A, and AG: supervision and writing – review & editing. AF: data curation, writing – review & editing. AB and TB: conceptualization, supervision, writing, and validation – review & editing. All authors contributed to the article and approved the submitted version.

## Conflict of interest

The authors declare that the research was conducted in the absence of any commercial or financial relationships that could be construed as a potential conflict of interest.

## Publisher’s note

All claims expressed in this article are solely those of the authors and do not necessarily represent those of their affiliated organizations, or those of the publisher, the editors and the reviewers. Any product that may be evaluated in this article, or claim that may be made by its manufacturer, is not guaranteed or endorsed by the publisher.

## Supplementary material

SUPPLEMENTARY DATA SHEET 1 The supplementary document describes the complete statistical analysis, including the source codes of the statistics.
