# Supplementary material for: Risk of SARS-CoV-2 transmission in the close contacts in a small rural area in the Veneto Region (NE-Italy): past evidence for future scenarios
Source: Front Public Health. 2023 Sep 5;11:1223109. doi: 10.3389/fpubh.2023.1223109 (PMC10507707; doi:10.3389/fpubh.2023.1223109)
Supplement: SUPPLEMENTARY DATA SHEET 1 — The supplementary document describes the complete statistical analysis, including the source codes of the statistics. [file Data_Sheet_1.DOC]

FREQUENCIES VARIABLES=ETA
  /STATISTICS=STDDEV MINIMUM MAXIMUM MEAN MEDIAN
  /ORDER=ANALYSIS.


Frequenze


Note	
Output creato	27-NOV-2020 12:41:43	
Commenti		
Input	Dataset attivo	Dataset1	
	Filtro	<nessuno>	
	Peso	<nessuno>	
	Suddividi file	<nessuno>	
	N di righe nel file di dati di lavoro	54	
Gestione valori mancanti	Definizione di mancante	I valori mancanti definiti dall'utente vengono trattati come mancanti.	
	Casi utilizzati	Le statistiche sono basate su tutti i casi con dati validi.	
Sintassi	FREQUENCIES VARIABLES=ETA
  /STATISTICS=STDDEV MINIMUM MAXIMUM MEAN MEDIAN
  /ORDER=ANALYSIS.	
Risorse	Tempo processore	00:00:00,00	
	Tempo trascorso	00:00:00,00	


Statistiche	
ETA'  	
N	Valido	54	
	Mancante	0	
Media	40,54	
Mediana	40,00	
Deviazione std.	19,207	
Minimo	7	
Massimo	78	


ETA'	
	Frequenza	Percentuale	Percentuale valida	Percentuale cumulativa	
Valido	7	1	1,9	1,9	1,9	
	11	1	1,9	1,9	3,7	
	13	2	3,7	3,7	7,4	
	14	1	1,9	1,9	9,3	
	17	2	3,7	3,7	13,0	
	18	2	3,7	3,7	16,7	
	19	1	1,9	1,9	18,5	
	22	4	7,4	7,4	25,9	
	23	3	5,6	5,6	31,5	
	24	2	3,7	3,7	35,2	
	26	1	1,9	1,9	37,0	
	34	1	1,9	1,9	38,9	
	36	1	1,9	1,9	40,7	
	37	2	3,7	3,7	44,4	
	38	1	1,9	1,9	46,3	
	39	1	1,9	1,9	48,1	
	40	2	3,7	3,7	51,9	
	46	2	3,7	3,7	55,6	
	47	1	1,9	1,9	57,4	
	50	2	3,7	3,7	61,1	
	51	1	1,9	1,9	63,0	
	52	1	1,9	1,9	64,8	
	53	2	3,7	3,7	68,5	
	54	2	3,7	3,7	72,2	
	57	1	1,9	1,9	74,1	
	58	1	1,9	1,9	75,9	
	59	2	3,7	3,7	79,6	
	60	1	1,9	1,9	81,5	
	61	1	1,9	1,9	83,3	
	62	2	3,7	3,7	87,0	
	64	2	3,7	3,7	90,7	
	66	2	3,7	3,7	94,4	
	69	2	3,7	3,7	98,1	
	78	1	1,9	1,9	100,0	
	Totale	54	100,0	100,0		

FREQUENCIES VARIABLES=Sesso
  /STATISTICS=STDDEV MINIMUM MAXIMUM MEAN MEDIAN
  /ORDER=ANALYSIS.


Frequenze


Note	
Output creato	27-NOV-2020 12:50:57	
Commenti		
Input	Dataset attivo	Dataset1	
	Filtro	<nessuno>	
	Peso	<nessuno>	
	Suddividi file	<nessuno>	
	N di righe nel file di dati di lavoro	54	
Gestione valori mancanti	Definizione di mancante	I valori mancanti definiti dall'utente vengono trattati come mancanti.	
	Casi utilizzati	Le statistiche sono basate su tutti i casi con dati validi.	
Sintassi	FREQUENCIES VARIABLES=Sesso
  /STATISTICS=STDDEV MINIMUM MAXIMUM MEAN MEDIAN
  /ORDER=ANALYSIS.	
Risorse	Tempo processore	00:00:00,00	
	Tempo trascorso	00:00:00,00	


Statistiche	
Sesso  	
N	Valido	0	
	Mancante	54	


Sesso	
	Frequenza	Percentuale	
Mancante	1	25	46,3	
	2	29	53,7	
	Totale	54	100,0	

FREQUENCIES VARIABLES=RelazioneNucleo
  /STATISTICS=STDDEV MINIMUM MAXIMUM MEAN MEDIAN
  /ORDER=ANALYSIS.


Frequenze


Note	
Output creato	30-NOV-2020 11:28:15	
Commenti		
Input	Dataset attivo	Dataset1	
	Filtro	<nessuno>	
	Peso	<nessuno>	
	Suddividi file	<nessuno>	
	N di righe nel file di dati di lavoro	54	
Gestione valori mancanti	Definizione di mancante	I valori mancanti definiti dall'utente vengono trattati come mancanti.	
	Casi utilizzati	Le statistiche sono basate su tutti i casi con dati validi.	
Sintassi	FREQUENCIES VARIABLES=RelazioneNucleo
  /STATISTICS=STDDEV MINIMUM MAXIMUM MEAN MEDIAN
  /ORDER=ANALYSIS.	
Risorse	Tempo processore	00:00:00,02	
	Tempo trascorso	00:00:00,03	


Statistiche	
RelazioneNucleo  	
N	Valido	54	
	Mancante	0	
Media	3,57	
Mediana	2,00	
Deviazione std.	3,124	
Minimo	0	
Massimo	8	


RelazioneNucleo	
	Frequenza	Percentuale	Percentuale valida	Percentuale cumulativa	
Valido	0	1	1,9	1,9	1,9	
	1	25	46,3	46,3	48,1	
	2	7	13,0	13,0	61,1	
	3	1	1,9	1,9	63,0	
	7	9	16,7	16,7	79,6	
	8	11	20,4	20,4	100,0	
	Totale	54	100,0	100,0		

FREQUENCIES VARIABLES=@1Sintomo @2Sintomo @3Sintomo @4Sintomo
  /STATISTICS=STDDEV MINIMUM MAXIMUM MEAN MEDIAN
  /ORDER=ANALYSIS.


Frequenze


Note	
Output creato	30-NOV-2020 11:36:21	
Commenti		
Input	Dataset attivo	Dataset1	
	Filtro	<nessuno>	
	Peso	<nessuno>	
	Suddividi file	<nessuno>	
	N di righe nel file di dati di lavoro	54	
Gestione valori mancanti	Definizione di mancante	I valori mancanti definiti dall'utente vengono trattati come mancanti.	
	Casi utilizzati	Le statistiche sono basate su tutti i casi con dati validi.	
Sintassi	FREQUENCIES VARIABLES=@1Sintomo @2Sintomo @3Sintomo @4Sintomo
  /STATISTICS=STDDEV MINIMUM MAXIMUM MEAN MEDIAN
  /ORDER=ANALYSIS.	
Risorse	Tempo processore	00:00:00,00	
	Tempo trascorso	00:00:00,00	


Statistiche	
	1 Sintomo	2 Sintomo	3 Sintomo	4 Sintomo	
N	Valido	54	22	11	1	
	Mancante	0	32	43	53	
Media	1,54	7,82	6,36	7,00	
Mediana	1,00	9,00	7,00	7,00	
Deviazione std.	2,447	1,893	1,502		
Minimo	0	3	2	7	
Massimo	11	10	7	7	


Tabella delle frequenze


1 Sintomo	
	Frequenza	Percentuale	Percentuale valida	Percentuale cumulativa	
Valido	0	21	38,9	38,9	38,9	
	1	23	42,6	42,6	81,5	
	3	1	1,9	1,9	83,3	
	4	4	7,4	7,4	90,7	
	7	3	5,6	5,6	96,3	
	9	1	1,9	1,9	98,1	
	11	1	1,9	1,9	100,0	
	Totale	54	100,0	100,0		


2 Sintomo	
	Frequenza	Percentuale	Percentuale valida	Percentuale cumulativa	
Valido	3	1	1,9	4,5	4,5	
	4	2	3,7	9,1	13,6	
	7	4	7,4	18,2	31,8	
	8	3	5,6	13,6	45,5	
	9	11	20,4	50,0	95,5	
	10	1	1,9	4,5	100,0	
	Totale	22	40,7	100,0		
Mancante	Sistema	32	59,3			
Totale	54	100,0			


3 Sintomo	
	Frequenza	Percentuale	Percentuale valida	Percentuale cumulativa	
Valido	2	1	1,9	9,1	9,1	
	6	2	3,7	18,2	27,3	
	7	8	14,8	72,7	100,0	
	Totale	11	20,4	100,0		
Mancante	Sistema	43	79,6			
Totale	54	100,0			


4 Sintomo	
	Frequenza	Percentuale	Percentuale valida	Percentuale cumulativa	
Valido	7	1	1,9	100,0	100,0	
Mancante	Sistema	53	98,1			
Totale	54	100,0			

FREQUENCIES VARIABLES=n.sintomi
  /STATISTICS=STDDEV MINIMUM MAXIMUM MEAN MEDIAN
  /ORDER=ANALYSIS.


Frequenze


Note	
Output creato	30-NOV-2020 12:02:39	
Commenti		
Input	Dataset attivo	Dataset1	
	Filtro	<nessuno>	
	Peso	<nessuno>	
	Suddividi file	<nessuno>	
	N di righe nel file di dati di lavoro	54	
Gestione valori mancanti	Definizione di mancante	I valori mancanti definiti dall'utente vengono trattati come mancanti.	
	Casi utilizzati	Le statistiche sono basate su tutti i casi con dati validi.	
Sintassi	FREQUENCIES VARIABLES=n.sintomi
  /STATISTICS=STDDEV MINIMUM MAXIMUM MEAN MEDIAN
  /ORDER=ANALYSIS.	
Risorse	Tempo processore	00:00:00,00	
	Tempo trascorso	00:00:00,00	


Statistiche	
n. sintomi  	
N	Valido	54	
	Mancante	0	
Media	1,24	
Mediana	1,00	
Deviazione std.	1,212	
Minimo	0	
Massimo	4	


n. sintomi	
	Frequenza	Percentuale	Percentuale valida	Percentuale cumulativa	
Valido	0	21	38,9	38,9	38,9	
	1	11	20,4	20,4	59,3	
	2	11	20,4	20,4	79,6	
	3	10	18,5	18,5	98,1	
	4	1	1,9	1,9	100,0	
	Totale	54	100,0	100,0		

CROSSTABS
  /TABLES=Esitodopo BY @1Sintomo @2Sintomo @3Sintomo @4Sintomo
  /FORMAT=AVALUE TABLES
  /CELLS=COUNT
  /COUNT ROUND CELL.


Tabelle di contingenza


Note	
Output creato	30-NOV-2020 12:06:22	
Commenti		
Input	Dataset attivo	Dataset1	
	Filtro	<nessuno>	
	Peso	<nessuno>	
	Suddividi file	<nessuno>	
	N di righe nel file di dati di lavoro	54	
Gestione valori mancanti	Definizione di mancante	I valori mancanti definiti dall'utente vengono trattati come mancanti.	
	Casi utilizzati	Le statistiche per ciascuna tabella sono basate su tutti i casi con dati validi nell'intervallo o negli intervalli specificati per tutte le variabili in ciascuna tabella.	
Sintassi	CROSSTABS
  /TABLES=Esitodopo BY @1Sintomo @2Sintomo @3Sintomo @4Sintomo
  /FORMAT=AVALUE TABLES
  /CELLS=COUNT
  /COUNT ROUND CELL.	
Risorse	Tempo processore	00:00:00,02	
	Tempo trascorso	00:00:00,02	
	Dimensioni richieste	2	
	Celle disponibili	524245	


Riepilogo elaborazione casi	
	Casi	
	Valido	Mancante	Totale	
	N	Percentuale	N	Percentuale	N	
Esito dopo * 1 Sintomo	54	100,0%	0	0,0%	54	
Esito dopo * 2 Sintomo	22	40,7%	32	59,3%	54	
Esito dopo * 3 Sintomo	11	20,4%	43	79,6%	54	
Esito dopo * 4 Sintomo	1	1,9%	53	98,1%	54	

Riepilogo elaborazione casi	
	Casi	
	Totale	
	Percentuale	
Esito dopo * 1 Sintomo	100,0%	
Esito dopo * 2 Sintomo	100,0%	
Esito dopo * 3 Sintomo	100,0%	
Esito dopo * 4 Sintomo	100,0%	


Tavola di contingenza Esito dopo * 1 Sintomo	
Conteggio  	
	1 Sintomo	
	0	1	3	4	7	9	11	
Esito dopo	1	4	11	1	1	1	1	0	
	2	17	12	0	3	2	0	1	
Totale	21	23	1	4	3	1	1	

Tavola di contingenza Esito dopo * 1 Sintomo	
Conteggio  	
	Totale	
		
Esito dopo	1	19	
	2	35	
Totale	54	


Tavola di contingenza Esito dopo * 2 Sintomo	
Conteggio  	
	2 Sintomo	Totale	
	3	4	7	8	9	10		
Esito dopo	1	1	1	3	1	5	0	11	
	2	0	1	1	2	6	1	11	
Totale	1	2	4	3	11	1	22	


Tavola di contingenza Esito dopo * 3 Sintomo	
Conteggio  	
	3 Sintomo	Totale	
	2	6	7		
Esito dopo	1	1	0	2	3	
	2	0	2	6	8	
Totale	1	2	8	11	


Tavola di contingenza Esito dopo * 4 Sintomo	
Conteggio  	
	4 Sintomo	Totale	
	7		
Esito dopo	1	1	1	
Totale	1	1	

CROSSTABS
  /TABLES=Esitodopo BY @1Sintomo @2Sintomo @3Sintomo @4Sintomo
  /FORMAT=AVALUE TABLES
  /STATISTICS=CHISQ CORR
  /CELLS=COUNT
  /COUNT ROUND CELL.


Tabelle di contingenza


Note	
Output creato	30-NOV-2020 12:06:54	
Commenti		
Input	Dataset attivo	Dataset1	
	Filtro	<nessuno>	
	Peso	<nessuno>	
	Suddividi file	<nessuno>	
	N di righe nel file di dati di lavoro	54	
Gestione valori mancanti	Definizione di mancante	I valori mancanti definiti dall'utente vengono trattati come mancanti.	
	Casi utilizzati	Le statistiche per ciascuna tabella sono basate su tutti i casi con dati validi nell'intervallo o negli intervalli specificati per tutte le variabili in ciascuna tabella.	
Sintassi	CROSSTABS
  /TABLES=Esitodopo BY @1Sintomo @2Sintomo @3Sintomo @4Sintomo
  /FORMAT=AVALUE TABLES
  /STATISTICS=CHISQ CORR
  /CELLS=COUNT
  /COUNT ROUND CELL.	
Risorse	Tempo processore	00:00:00,02	
	Tempo trascorso	00:00:00,02	
	Dimensioni richieste	2	
	Celle disponibili	524245	


Riepilogo elaborazione casi	
	Casi	
	Valido	Mancante	Totale	
	N	Percentuale	N	Percentuale	N	
Esito dopo * 1 Sintomo	54	100,0%	0	0,0%	54	
Esito dopo * 2 Sintomo	22	40,7%	32	59,3%	54	
Esito dopo * 3 Sintomo	11	20,4%	43	79,6%	54	
Esito dopo * 4 Sintomo	1	1,9%	53	98,1%	54	

Riepilogo elaborazione casi	
	Casi	
	Totale	
	Percentuale	
Esito dopo * 1 Sintomo	100,0%	
Esito dopo * 2 Sintomo	100,0%	
Esito dopo * 3 Sintomo	100,0%	
Esito dopo * 4 Sintomo	100,0%	


Esito dopo * 1 Sintomo


Tabella di contingenza	
Conteggio  	
	1 Sintomo	
	0	1	3	4	7	9	11	
Esito dopo	1	4	11	1	1	1	1	0	
	2	17	12	0	3	2	0	1	
Totale	21	23	1	4	3	1	1	

Tabella di contingenza	
Conteggio  	
	Totale	
		
Esito dopo	1	19	
	2	35	
Totale	54	


Test del chi-quadrato	
	Valore	df	Significatività asintotica (bilaterale)	
Chi-quadrato di Pearson	8,423a	6	,209	
Rapporto di verosimiglianza	9,438	6	,150	
Associazione lineare per lineare	,312	1	,577	
N di casi validi	54			

a. 10 celle (71,4%) hanno un conteggio previsto inferiore a 5. Il conteggio previsto minimo è ,35.	


Misure simmetriche	
	Valore	Errore standard asintoticoa	T approssimatob	
Intervallo per intervallo	R di Pearson	-,077	,136	-,555	
Ordinale per ordinale	Correlazione di Spearman	-,217	,128	-1,602	
N di casi validi	54			

Misure simmetriche	
	Significatività approssimata	
Intervallo per intervallo	R di Pearson	,581c	
Ordinale per ordinale	Correlazione di Spearman	,115c	
N di casi validi		

a. Non viene assunta l'ipotesi nulla.	
b. Viene utilizzato l'errore standard asintotico presumendo l'ipotesi nulla.	
c. Basato sull'approssimazione normale.	


Esito dopo * 2 Sintomo


Tabella di contingenza	
Conteggio  	
	2 Sintomo	Totale	
	3	4	7	8	9	10		
Esito dopo	1	1	1	3	1	5	0	11	
	2	0	1	1	2	6	1	11	
Totale	1	2	4	3	11	1	22	


Test del chi-quadrato	
	Valore	df	Significatività asintotica (bilaterale)	
Chi-quadrato di Pearson	3,424a	5	,635	
Rapporto di verosimiglianza	4,250	5	,514	
Associazione lineare per lineare	1,268	1	,260	
N di casi validi	22			

a. 10 celle (83,3%) hanno un conteggio previsto inferiore a 5. Il conteggio previsto minimo è ,50.	


Misure simmetriche	
	Valore	Errore standard asintoticoa	T approssimatob	
Intervallo per intervallo	R di Pearson	,246	,193	1,134	
Ordinale per ordinale	Correlazione di Spearman	,269	,198	1,250	
N di casi validi	22			

Misure simmetriche	
	Significatività approssimata	
Intervallo per intervallo	R di Pearson	,270c	
Ordinale per ordinale	Correlazione di Spearman	,226c	
N di casi validi		

a. Non viene assunta l'ipotesi nulla.	
b. Viene utilizzato l'errore standard asintotico presumendo l'ipotesi nulla.	
c. Basato sull'approssimazione normale.	


Esito dopo * 3 Sintomo


Tabella di contingenza	
Conteggio  	
	3 Sintomo	Totale	
	2	6	7		
Esito dopo	1	1	0	2	3	
	2	0	2	6	8	
Totale	1	2	8	11	


Test del chi-quadrato	
	Valore	df	Significatività asintotica (bilaterale)	
Chi-quadrato di Pearson	3,438a	2	,179	
Rapporto di verosimiglianza	3,894	2	,143	
Associazione lineare per lineare	1,942	1	,163	
N di casi validi	11			

a. 5 celle (83,3%) hanno un conteggio previsto inferiore a 5. Il conteggio previsto minimo è ,27.	


Misure simmetriche	
	Valore	Errore standard asintoticoa	T approssimatob	
Intervallo per intervallo	R di Pearson	,441	,279	1,473	
Ordinale per ordinale	Correlazione di Spearman	,165	,344	,501	
N di casi validi	11			

Misure simmetriche	
	Significatività approssimata	
Intervallo per intervallo	R di Pearson	,175c	
Ordinale per ordinale	Correlazione di Spearman	,628c	
N di casi validi		

a. Non viene assunta l'ipotesi nulla.	
b. Viene utilizzato l'errore standard asintotico presumendo l'ipotesi nulla.	
c. Basato sull'approssimazione normale.	


Esito dopo * 4 Sintomo


Tabella di contingenza	
Conteggio  	
	4 Sintomo	Totale	
	7		
Esito dopo	1	1	1	
Totale	1	1	


Test del chi-quadrato	
	Valore	
Chi-quadrato di Pearson	.a	
N di casi validi	1	

a. Non viene calcolata alcuna statistica perché Esito dopo e 4 Sintomo sono costanti.	


Misure simmetriche	
	Valore	
Intervallo per intervallo	R di Pearson	.a	
N di casi validi	1	

a. Non viene calcolata alcuna statistica perché Esito dopo e 4 Sintomo sono costanti.	

CROSSTABS
  /TABLES=Esitodopo BY n.sintomi
  /FORMAT=AVALUE TABLES
  /STATISTICS=CHISQ CORR
  /CELLS=COUNT
  /COUNT ROUND CELL.


Tabelle di contingenza


Note	
Output creato	30-NOV-2020 12:26:06	
Commenti		
Input	Dataset attivo	Dataset1	
	Filtro	<nessuno>	
	Peso	<nessuno>	
	Suddividi file	<nessuno>	
	N di righe nel file di dati di lavoro	54	
Gestione valori mancanti	Definizione di mancante	I valori mancanti definiti dall'utente vengono trattati come mancanti.	
	Casi utilizzati	Le statistiche per ciascuna tabella sono basate su tutti i casi con dati validi nell'intervallo o negli intervalli specificati per tutte le variabili in ciascuna tabella.	
Sintassi	CROSSTABS
  /TABLES=Esitodopo BY n.sintomi
  /FORMAT=AVALUE TABLES
  /STATISTICS=CHISQ CORR
  /CELLS=COUNT
  /COUNT ROUND CELL.	
Risorse	Tempo processore	00:00:00,00	
	Tempo trascorso	00:00:00,00	
	Dimensioni richieste	2	
	Celle disponibili	524245	


Riepilogo elaborazione casi	
	Casi	
	Valido	Mancante	Totale	
	N	Percentuale	N	Percentuale	N	
Esito dopo * n. sintomi	54	100,0%	0	0,0%	54	

Riepilogo elaborazione casi	
	Casi	
	Totale	
	Percentuale	
Esito dopo * n. sintomi	100,0%	


Tavola di contingenza Esito dopo * n. sintomi	
Conteggio  	
	n. sintomi	Totale	
	0	1	2	3	4		
Esito dopo	1	4	4	8	2	1	19	
	2	17	7	3	8	0	35	
Totale	21	11	11	10	1	54	


Test del chi-quadrato	
	Valore	df	Significatività asintotica (bilaterale)	
Chi-quadrato di Pearson	12,056a	4	,017	
Rapporto di verosimiglianza	12,277	4	,015	
Associazione lineare per lineare	2,282	1	,131	
N di casi validi	54			

a. 5 celle (50,0%) hanno un conteggio previsto inferiore a 5. Il conteggio previsto minimo è ,35.	


Misure simmetriche	
	Valore	Errore standard asintoticoa	T approssimatob	
Intervallo per intervallo	R di Pearson	-,208	,128	-1,530	
Ordinale per ordinale	Correlazione di Spearman	-,219	,130	-1,622	
N di casi validi	54			

Misure simmetriche	
	Significatività approssimata	
Intervallo per intervallo	R di Pearson	,132c	
Ordinale per ordinale	Correlazione di Spearman	,111c	
N di casi validi		

a. Non viene assunta l'ipotesi nulla.	
b. Viene utilizzato l'errore standard asintotico presumendo l'ipotesi nulla.	
c. Basato sull'approssimazione normale.	

CROSSTABS
  /TABLES=n.sintomi BY Esitodopo
  /FORMAT=AVALUE TABLES
  /STATISTICS=CHISQ CORR
  /CELLS=COUNT
  /COUNT ROUND CELL.


Tabelle di contingenza


Note	
Output creato	30-NOV-2020 12:26:46	
Commenti		
Input	Dataset attivo	Dataset1	
	Filtro	<nessuno>	
	Peso	<nessuno>	
	Suddividi file	<nessuno>	
	N di righe nel file di dati di lavoro	54	
Gestione valori mancanti	Definizione di mancante	I valori mancanti definiti dall'utente vengono trattati come mancanti.	
	Casi utilizzati	Le statistiche per ciascuna tabella sono basate su tutti i casi con dati validi nell'intervallo o negli intervalli specificati per tutte le variabili in ciascuna tabella.	
Sintassi	CROSSTABS
  /TABLES=n.sintomi BY Esitodopo
  /FORMAT=AVALUE TABLES
  /STATISTICS=CHISQ CORR
  /CELLS=COUNT
  /COUNT ROUND CELL.	
Risorse	Tempo processore	00:00:00,02	
	Tempo trascorso	00:00:00,02	
	Dimensioni richieste	2	
	Celle disponibili	524245	


Riepilogo elaborazione casi	
	Casi	
	Valido	Mancante	Totale	
	N	Percentuale	N	Percentuale	N	
n. sintomi * Esito dopo	54	100,0%	0	0,0%	54	

Riepilogo elaborazione casi	
	Casi	
	Totale	
	Percentuale	
n. sintomi * Esito dopo	100,0%	


Tavola di contingenza n. sintomi * Esito dopo	
Conteggio  	
	Esito dopo	Totale	
	1	2		
n. sintomi	0	4	17	21	
	1	4	7	11	
	2	8	3	11	
	3	2	8	10	
	4	1	0	1	
Totale	19	35	54	


Test del chi-quadrato	
	Valore	df	Significatività asintotica (bilaterale)	
Chi-quadrato di Pearson	12,056a	4	,017	
Rapporto di verosimiglianza	12,277	4	,015	
Associazione lineare per lineare	2,282	1	,131	
N di casi validi	54			

a. 5 celle (50,0%) hanno un conteggio previsto inferiore a 5. Il conteggio previsto minimo è ,35.	


Misure simmetriche	
	Valore	Errore standard asintoticoa	T approssimatob	
Intervallo per intervallo	R di Pearson	-,208	,128	-1,530	
Ordinale per ordinale	Correlazione di Spearman	-,219	,130	-1,622	
N di casi validi	54			

Misure simmetriche	
	Significatività approssimata	
Intervallo per intervallo	R di Pearson	,132c	
Ordinale per ordinale	Correlazione di Spearman	,111c	
N di casi validi		

a. Non viene assunta l'ipotesi nulla.	
b. Viene utilizzato l'errore standard asintotico presumendo l'ipotesi nulla.	
c. Basato sull'approssimazione normale.	

CROSSTABS
  /TABLES=n.sintomi BY ESITOprima BY Esitodopo
  /FORMAT=AVALUE TABLES
  /STATISTICS=CHISQ CORR
  /CELLS=COUNT
  /COUNT ROUND CELL.


Tabelle di contingenza


Note	
Output creato	30-NOV-2020 12:27:43	
Commenti		
Input	Dataset attivo	Dataset1	
	Filtro	<nessuno>	
	Peso	<nessuno>	
	Suddividi file	<nessuno>	
	N di righe nel file di dati di lavoro	54	
Gestione valori mancanti	Definizione di mancante	I valori mancanti definiti dall'utente vengono trattati come mancanti.	
	Casi utilizzati	Le statistiche per ciascuna tabella sono basate su tutti i casi con dati validi nell'intervallo o negli intervalli specificati per tutte le variabili in ciascuna tabella.	
Sintassi	CROSSTABS
  /TABLES=n.sintomi BY ESITOprima BY Esitodopo
  /FORMAT=AVALUE TABLES
  /STATISTICS=CHISQ CORR
  /CELLS=COUNT
  /COUNT ROUND CELL.	
Risorse	Tempo processore	00:00:00,02	
	Tempo trascorso	00:00:00,02	
	Dimensioni richieste	3	
	Celle disponibili	449353	


Riepilogo elaborazione casi	
	Casi	
	Valido	Mancante	Totale	
	N	Percentuale	N	Percentuale	N	
n. sintomi * ESITO prima * Esito dopo	54	100,0%	0	0,0%	54	

Riepilogo elaborazione casi	
	Casi	
	Totale	
	Percentuale	
n. sintomi * ESITO prima * Esito dopo	100,0%	


Tavola di contingenza n. sintomi * ESITO prima * Esito dopo	
Conteggio  	
Esito dopo	ESITO prima	Totale	
	0	1		
1	n. sintomi	0	2	2	4	
		1	2	2	4	
		2	5	3	8	
		3	0	2	2	
		4	0	1	1	
	Totale	9	10	19	
2	n. sintomi	0	16	1	17	
		1	7	0	7	
		2	1	2	3	
		3	5	3	8	
	Totale	29	6	35	
Totale	n. sintomi	0	18	3	21	
		1	9	2	11	
		2	6	5	11	
		3	5	5	10	
		4	0	1	1	
	Totale	38	16	54	


Test del chi-quadrato	
Esito dopo	Valore	df	Significatività asintotica (bilaterale)	
1	Chi-quadrato di Pearson	3,457b	4	,484	
	Rapporto di verosimiglianza	4,612	4	,330	
	Associazione lineare per lineare	,820	1	,365	
	N di casi validi	19			
2	Chi-quadrato di Pearson	10,480c	3	,015	
	Rapporto di verosimiglianza	10,060	3	,018	
	Associazione lineare per lineare	5,842	1	,016	
	N di casi validi	35			
Totale	Chi-quadrato di Pearson	8,749a	4	,068	
	Rapporto di verosimiglianza	8,954	4	,062	
	Associazione lineare per lineare	7,513	1	,006	
	N di casi validi	54			

a. 5 celle (50,0%) hanno un conteggio previsto inferiore a 5. Il conteggio previsto minimo è ,30.	
b. 10 celle (100,0%) hanno un conteggio previsto inferiore a 5. Il conteggio previsto minimo è ,47.	
c. 5 celle (62,5%) hanno un conteggio previsto inferiore a 5. Il conteggio previsto minimo è ,51.	


Misure simmetriche	
Esito dopo	Valore	Errore standard asintoticoa	
1	Intervallo per intervallo	R di Pearson	,213	,206	
	Ordinale per ordinale	Correlazione di Spearman	,192	,223	
	N di casi validi	19		
2	Intervallo per intervallo	R di Pearson	,414	,159	
	Ordinale per ordinale	Correlazione di Spearman	,383	,154	
	N di casi validi	35		
Totale	Intervallo per intervallo	R di Pearson	,376	,126	
	Ordinale per ordinale	Correlazione di Spearman	,363	,125	
	N di casi validi	54		

Misure simmetriche	
Esito dopo	T approssimatob	Significatività approssimata	
1	Intervallo per intervallo	R di Pearson	,901	,380c	
	Ordinale per ordinale	Correlazione di Spearman	,806	,431c	
	N di casi validi			
2	Intervallo per intervallo	R di Pearson	2,616	,013c	
	Ordinale per ordinale	Correlazione di Spearman	2,384	,023c	
	N di casi validi			
Totale	Intervallo per intervallo	R di Pearson	2,931	,005c	
	Ordinale per ordinale	Correlazione di Spearman	2,805	,007c	
	N di casi validi			

a. Non viene assunta l'ipotesi nulla.	
b. Viene utilizzato l'errore standard asintotico presumendo l'ipotesi nulla.	
c. Basato sull'approssimazione normale.	

CROSSTABS
  /TABLES=n.sintomi BY ContattiContagi BY Esitodopo
  /FORMAT=AVALUE TABLES
  /STATISTICS=CHISQ CORR
  /CELLS=COUNT
  /COUNT ROUND CELL.


Tabelle di contingenza


Note	
Output creato	30-NOV-2020 12:28:57	
Commenti		
Input	Dataset attivo	Dataset1	
	Filtro	<nessuno>	
	Peso	<nessuno>	
	Suddividi file	<nessuno>	
	N di righe nel file di dati di lavoro	54	
Gestione valori mancanti	Definizione di mancante	I valori mancanti definiti dall'utente vengono trattati come mancanti.	
	Casi utilizzati	Le statistiche per ciascuna tabella sono basate su tutti i casi con dati validi nell'intervallo o negli intervalli specificati per tutte le variabili in ciascuna tabella.	
Sintassi	CROSSTABS
  /TABLES=n.sintomi BY ContattiContagi BY Esitodopo
  /FORMAT=AVALUE TABLES
  /STATISTICS=CHISQ CORR
  /CELLS=COUNT
  /COUNT ROUND CELL.	
Risorse	Tempo processore	00:00:00,00	
	Tempo trascorso	00:00:00,02	
	Dimensioni richieste	3	
	Celle disponibili	449353	


Riepilogo elaborazione casi	
	Casi	
	Valido	Mancante	Totale	
	N	Percentuale	N	Percentuale	N	
n. sintomi * ContattiContagi * Esito dopo	54	100,0%	0	0,0%	54	

Riepilogo elaborazione casi	
	Casi	
	Totale	
	Percentuale	
n. sintomi * ContattiContagi * Esito dopo	100,0%	


Tavola di contingenza n. sintomi * ContattiContagi * Esito dopo	
Conteggio  	
Esito dopo	ContattiContagi	Totale	
	0	1	2		
1	n. sintomi	0	0	4		4	
		1	1	3		4	
		2	0	8		8	
		3	0	2		2	
		4	0	1		1	
	Totale	1	18		19	
2	n. sintomi	0		17	0	17	
		1		7	0	7	
		2		3	0	3	
		3		7	1	8	
	Totale		34	1	35	
Totale	n. sintomi	0	0	21	0	21	
		1	1	10	0	11	
		2	0	11	0	11	
		3	0	9	1	10	
		4	0	1	0	1	
	Totale	1	52	1	54	


Test del chi-quadrato	
Esito dopo	Valore	df	Significatività asintotica (bilaterale)	
1	Chi-quadrato di Pearson	3,958b	4	,412	
	Rapporto di verosimiglianza	3,337	4	,503	
	Associazione lineare per lineare	,281	1	,596	
	N di casi validi	19			
2	Chi-quadrato di Pearson	3,474c	3	,324	
	Rapporto di verosimiglianza	3,054	3	,383	
	Associazione lineare per lineare	2,546	1	,111	
	N di casi validi	35			
Totale	Chi-quadrato di Pearson	8,430a	8	,393	
	Rapporto di verosimiglianza	6,677	8	,572	
	Associazione lineare per lineare	1,361	1	,243	
	N di casi validi	54			

a. 11 celle (73,3%) hanno un conteggio previsto inferiore a 5. Il conteggio previsto minimo è ,02.	
b. 9 celle (90,0%) hanno un conteggio previsto inferiore a 5. Il conteggio previsto minimo è ,05.	
c. 5 celle (62,5%) hanno un conteggio previsto inferiore a 5. Il conteggio previsto minimo è ,09.	


Misure simmetriche	
Esito dopo	Valore	Errore standard asintoticoa	
1	Intervallo per intervallo	R di Pearson	,125	,081	
	Ordinale per ordinale	Correlazione di Spearman	,158	,109	
	N di casi validi	19		
2	Intervallo per intervallo	R di Pearson	,274	,132	
	Ordinale per ordinale	Correlazione di Spearman	,246	,120	
	N di casi validi	35		
Totale	Intervallo per intervallo	R di Pearson	,160	,101	
	Ordinale per ordinale	Correlazione di Spearman	,139	,108	
	N di casi validi	54		

Misure simmetriche	
Esito dopo	T approssimatob	Significatività approssimata	
1	Intervallo per intervallo	R di Pearson	,520	,610c	
	Ordinale per ordinale	Correlazione di Spearman	,660	,518c	
	N di casi validi			
2	Intervallo per intervallo	R di Pearson	1,634	,112c	
	Ordinale per ordinale	Correlazione di Spearman	1,461	,154c	
	N di casi validi			
Totale	Intervallo per intervallo	R di Pearson	1,171	,247c	
	Ordinale per ordinale	Correlazione di Spearman	1,009	,318c	
	N di casi validi			

a. Non viene assunta l'ipotesi nulla.	
b. Viene utilizzato l'errore standard asintotico presumendo l'ipotesi nulla.	
c. Basato sull'approssimazione normale.	

CROSSTABS
  /TABLES=n.sintomi BY PregressaQuarantena BY Esitodopo
  /FORMAT=AVALUE TABLES
  /STATISTICS=CHISQ CORR
  /CELLS=COUNT
  /COUNT ROUND CELL.


Tabelle di contingenza


Note	
Output creato	30-NOV-2020 12:29:11	
Commenti		
Input	Dataset attivo	Dataset1	
	Filtro	<nessuno>	
	Peso	<nessuno>	
	Suddividi file	<nessuno>	
	N di righe nel file di dati di lavoro	54	
Gestione valori mancanti	Definizione di mancante	I valori mancanti definiti dall'utente vengono trattati come mancanti.	
	Casi utilizzati	Le statistiche per ciascuna tabella sono basate su tutti i casi con dati validi nell'intervallo o negli intervalli specificati per tutte le variabili in ciascuna tabella.	
Sintassi	CROSSTABS
  /TABLES=n.sintomi BY PregressaQuarantena BY Esitodopo
  /FORMAT=AVALUE TABLES
  /STATISTICS=CHISQ CORR
  /CELLS=COUNT
  /COUNT ROUND CELL.	
Risorse	Tempo processore	00:00:00,00	
	Tempo trascorso	00:00:00,00	
	Dimensioni richieste	3	
	Celle disponibili	449353	


Riepilogo elaborazione casi	
	Casi	
	Valido	Mancante	Totale	
	N	Percentuale	N	Percentuale	N	
n. sintomi * PregressaQuarantena * Esito dopo	54	100,0%	0	0,0%	54	

Riepilogo elaborazione casi	
	Casi	
	Totale	
	Percentuale	
n. sintomi * PregressaQuarantena * Esito dopo	100,0%	


Tavola di contingenza n. sintomi * PregressaQuarantena * Esito dopo	
Conteggio  	
Esito dopo	PregressaQuarantena	Totale	
	1	2		
1	n. sintomi	0	2	2	4	
		1	1	3	4	
		2	5	3	8	
		3	0	2	2	
		4	0	1	1	
	Totale	8	11	19	
2	n. sintomi	0		17	17	
		1		7	7	
		2		3	3	
		3		8	8	
	Totale		35	35	
Totale	n. sintomi	0	2	19	21	
		1	1	10	11	
		2	5	6	11	
		3	0	10	10	
		4	0	1	1	
	Totale	8	46	54	


Test del chi-quadrato	
Esito dopo	Valore	df	Significatività asintotica (bilaterale)	
1	Chi-quadrato di Pearson	4,129b	4	,389	
	Rapporto di verosimiglianza	5,235	4	,264	
	Associazione lineare per lineare	,457	1	,499	
	N di casi validi	19			
2	Chi-quadrato di Pearson	.c			
	N di casi validi	35			
Totale	Chi-quadrato di Pearson	10,847a	4	,028	
	Rapporto di verosimiglianza	10,235	4	,037	
	Associazione lineare per lineare	,115	1	,734	
	N di casi validi	54			

a. 6 celle (60,0%) hanno un conteggio previsto inferiore a 5. Il conteggio previsto minimo è ,15.	
b. 10 celle (100,0%) hanno un conteggio previsto inferiore a 5. Il conteggio previsto minimo è ,42.	
c. Non viene calcolata alcuna statistica perché PregressaQuarantena è una costante.	


Misure simmetriche	
Esito dopo	Valore	Errore standard asintoticoa	
1	Intervallo per intervallo	R di Pearson	,159	,204	
	Ordinale per ordinale	Correlazione di Spearman	,123	,224	
	N di casi validi	19		
2	Intervallo per intervallo	R di Pearson	.d		
	N di casi validi	35		
Totale	Intervallo per intervallo	R di Pearson	-,047	,106	
	Ordinale per ordinale	Correlazione di Spearman	-,066	,116	
	N di casi validi	54		

Misure simmetriche	
Esito dopo	T approssimatob	Significatività approssimata	
1	Intervallo per intervallo	R di Pearson	,666	,515c	
	Ordinale per ordinale	Correlazione di Spearman	,509	,617c	
	N di casi validi			
2	Intervallo per intervallo	R di Pearson			
	N di casi validi			
Totale	Intervallo per intervallo	R di Pearson	-,337	,738c	
	Ordinale per ordinale	Correlazione di Spearman	-,479	,634c	
	N di casi validi			

a. Non viene assunta l'ipotesi nulla.	
b. Viene utilizzato l'errore standard asintotico presumendo l'ipotesi nulla.	
c. Basato sull'approssimazione normale.	
d. Non viene calcolata alcuna statistica perché PregressaQuarantena è una costante.	

CROSSTABS
  /TABLES=n.sintomi BY Esitodopo
  /FORMAT=AVALUE TABLES
  /STATISTICS=CHISQ CORR
  /CELLS=COUNT
  /COUNT ROUND CELL.


Tabelle di contingenza


Note	
Output creato	30-NOV-2020 12:29:36	
Commenti		
Input	Dataset attivo	Dataset1	
	Filtro	<nessuno>	
	Peso	<nessuno>	
	Suddividi file	<nessuno>	
	N di righe nel file di dati di lavoro	54	
Gestione valori mancanti	Definizione di mancante	I valori mancanti definiti dall'utente vengono trattati come mancanti.	
	Casi utilizzati	Le statistiche per ciascuna tabella sono basate su tutti i casi con dati validi nell'intervallo o negli intervalli specificati per tutte le variabili in ciascuna tabella.	
Sintassi	CROSSTABS
  /TABLES=n.sintomi BY Esitodopo
  /FORMAT=AVALUE TABLES
  /STATISTICS=CHISQ CORR
  /CELLS=COUNT
  /COUNT ROUND CELL.	
Risorse	Tempo processore	00:00:00,00	
	Tempo trascorso	00:00:00,00	
	Dimensioni richieste	2	
	Celle disponibili	524245	


Riepilogo elaborazione casi	
	Casi	
	Valido	Mancante	Totale	
	N	Percentuale	N	Percentuale	N	
n. sintomi * Esito dopo	54	100,0%	0	0,0%	54	

Riepilogo elaborazione casi	
	Casi	
	Totale	
	Percentuale	
n. sintomi * Esito dopo	100,0%	


Tavola di contingenza n. sintomi * Esito dopo	
Conteggio  	
	Esito dopo	Totale	
	1	2		
n. sintomi	0	4	17	21	
	1	4	7	11	
	2	8	3	11	
	3	2	8	10	
	4	1	0	1	
Totale	19	35	54	


Test del chi-quadrato	
	Valore	df	Significatività asintotica (bilaterale)	
Chi-quadrato di Pearson	12,056a	4	,017	
Rapporto di verosimiglianza	12,277	4	,015	
Associazione lineare per lineare	2,282	1	,131	
N di casi validi	54			

a. 5 celle (50,0%) hanno un conteggio previsto inferiore a 5. Il conteggio previsto minimo è ,35.	


Misure simmetriche	
	Valore	Errore standard asintoticoa	T approssimatob	
Intervallo per intervallo	R di Pearson	-,208	,128	-1,530	
Ordinale per ordinale	Correlazione di Spearman	-,219	,130	-1,622	
N di casi validi	54			

Misure simmetriche	
	Significatività approssimata	
Intervallo per intervallo	R di Pearson	,132c	
Ordinale per ordinale	Correlazione di Spearman	,111c	
N di casi validi		

a. Non viene assunta l'ipotesi nulla.	
b. Viene utilizzato l'errore standard asintotico presumendo l'ipotesi nulla.	
c. Basato sull'approssimazione normale.	

CROSSTABS
  /TABLES=n.sintomi ETA BY Esitodopo
  /FORMAT=AVALUE TABLES
  /STATISTICS=CHISQ CORR
  /CELLS=COUNT
  /COUNT ROUND CELL.


Tabelle di contingenza


Note	
Output creato	30-NOV-2020 12:36:49	
Commenti		
Input	Dataset attivo	Dataset1	
	Filtro	<nessuno>	
	Peso	<nessuno>	
	Suddividi file	<nessuno>	
	N di righe nel file di dati di lavoro	54	
Gestione valori mancanti	Definizione di mancante	I valori mancanti definiti dall'utente vengono trattati come mancanti.	
	Casi utilizzati	Le statistiche per ciascuna tabella sono basate su tutti i casi con dati validi nell'intervallo o negli intervalli specificati per tutte le variabili in ciascuna tabella.	
Sintassi	CROSSTABS
  /TABLES=n.sintomi ETA BY Esitodopo
  /FORMAT=AVALUE TABLES
  /STATISTICS=CHISQ CORR
  /CELLS=COUNT
  /COUNT ROUND CELL.	
Risorse	Tempo processore	00:00:00,03	
	Tempo trascorso	00:00:00,03	
	Dimensioni richieste	2	
	Celle disponibili	524245	


Riepilogo elaborazione casi	
	Casi	
	Valido	Mancante	Totale	
	N	Percentuale	N	Percentuale	N	
n. sintomi * Esito dopo	54	100,0%	0	0,0%	54	
ETA' * Esito dopo	54	100,0%	0	0,0%	54	

Riepilogo elaborazione casi	
	Casi	
	Totale	
	Percentuale	
n. sintomi * Esito dopo	100,0%	
ETA' * Esito dopo	100,0%	


n. sintomi * Esito dopo


Tabella di contingenza	
Conteggio  	
	Esito dopo	Totale	
	1	2		
n. sintomi	0	4	17	21	
	1	4	7	11	
	2	8	3	11	
	3	2	8	10	
	4	1	0	1	
Totale	19	35	54	


Test del chi-quadrato	
	Valore	df	Significatività asintotica (bilaterale)	
Chi-quadrato di Pearson	12,056a	4	,017	
Rapporto di verosimiglianza	12,277	4	,015	
Associazione lineare per lineare	2,282	1	,131	
N di casi validi	54			

a. 5 celle (50,0%) hanno un conteggio previsto inferiore a 5. Il conteggio previsto minimo è ,35.	


Misure simmetriche	
	Valore	Errore standard asintoticoa	T approssimatob	
Intervallo per intervallo	R di Pearson	-,208	,128	-1,530	
Ordinale per ordinale	Correlazione di Spearman	-,219	,130	-1,622	
N di casi validi	54			

Misure simmetriche	
	Significatività approssimata	
Intervallo per intervallo	R di Pearson	,132c	
Ordinale per ordinale	Correlazione di Spearman	,111c	
N di casi validi		

a. Non viene assunta l'ipotesi nulla.	
b. Viene utilizzato l'errore standard asintotico presumendo l'ipotesi nulla.	
c. Basato sull'approssimazione normale.	


ETA' * Esito dopo


Tabella di contingenza	
Conteggio  	
	Esito dopo	Totale	
	1	2		
ETA'	7	0	1	1	
	11	0	1	1	
	13	1	1	2	
	14	0	1	1	
	17	1	1	2	
	18	0	2	2	
	19	0	1	1	
	22	1	3	4	
	23	1	2	3	
	24	0	2	2	
	26	0	1	1	
	34	0	1	1	
	36	0	1	1	
	37	1	1	2	
	38	1	0	1	
	39	0	1	1	
	40	2	0	2	
	46	0	2	2	
	47	1	0	1	
	50	1	1	2	
	51	1	0	1	
	52	0	1	1	
	53	1	1	2	
	54	0	2	2	
	57	0	1	1	
	58	0	1	1	
	59	0	2	2	
	60	1	0	1	
	61	1	0	1	
	62	1	1	2	
	64	1	1	2	
	66	2	0	2	
	69	1	1	2	
	78	0	1	1	
Totale	19	35	54	


Test del chi-quadrato	
	Valore	df	Significatività asintotica (bilaterale)	
Chi-quadrato di Pearson	30,248a	33	,605	
Rapporto di verosimiglianza	39,549	33	,201	
Associazione lineare per lineare	2,606	1	,106	
N di casi validi	54			

a. 68 celle (100,0%) hanno un conteggio previsto inferiore a 5. Il conteggio previsto minimo è ,35.	


Misure simmetriche	
	Valore	Errore standard asintoticoa	T approssimatob	
Intervallo per intervallo	R di Pearson	-,222	,128	-1,640	
Ordinale per ordinale	Correlazione di Spearman	-,227	,130	-1,677	
N di casi validi	54			

Misure simmetriche	
	Significatività approssimata	
Intervallo per intervallo	R di Pearson	,107c	
Ordinale per ordinale	Correlazione di Spearman	,100c	
N di casi validi		

a. Non viene assunta l'ipotesi nulla.	
b. Viene utilizzato l'errore standard asintotico presumendo l'ipotesi nulla.	
c. Basato sull'approssimazione normale.	

CROSSTABS
  /TABLES=@1Sintomo @2Sintomo @3Sintomo @4Sintomo BY n.sintomi BY Esitodopo
  /FORMAT=AVALUE TABLES
  /STATISTICS=CHISQ CORR
  /CELLS=COUNT
  /COUNT ROUND CELL.


Tabelle di contingenza


Note	
Output creato	30-NOV-2020 12:38:59	
Commenti		
Input	Dataset attivo	Dataset1	
	Filtro	<nessuno>	
	Peso	<nessuno>	
	Suddividi file	<nessuno>	
	N di righe nel file di dati di lavoro	54	
Gestione valori mancanti	Definizione di mancante	I valori mancanti definiti dall'utente vengono trattati come mancanti.	
	Casi utilizzati	Le statistiche per ciascuna tabella sono basate su tutti i casi con dati validi nell'intervallo o negli intervalli specificati per tutte le variabili in ciascuna tabella.	
Sintassi	CROSSTABS
  /TABLES=@1Sintomo @2Sintomo @3Sintomo @4Sintomo BY n.sintomi BY Esitodopo
  /FORMAT=AVALUE TABLES
  /STATISTICS=CHISQ CORR
  /CELLS=COUNT
  /COUNT ROUND CELL.	
Risorse	Tempo processore	00:00:00,02	
	Tempo trascorso	00:00:00,02	
	Dimensioni richieste	3	
	Celle disponibili	449353	


Riepilogo elaborazione casi	
	Casi	
	Valido	Mancante	Totale	
	N	Percentuale	N	Percentuale	N	
1 Sintomo * n. sintomi * Esito dopo	54	100,0%	0	0,0%	54	
2 Sintomo * n. sintomi * Esito dopo	22	40,7%	32	59,3%	54	
3 Sintomo * n. sintomi * Esito dopo	11	20,4%	43	79,6%	54	
4 Sintomo * n. sintomi * Esito dopo	1	1,9%	53	98,1%	54	

Riepilogo elaborazione casi	
	Casi	
	Totale	
	Percentuale	
1 Sintomo * n. sintomi * Esito dopo	100,0%	
2 Sintomo * n. sintomi * Esito dopo	100,0%	
3 Sintomo * n. sintomi * Esito dopo	100,0%	
4 Sintomo * n. sintomi * Esito dopo	100,0%	


1 Sintomo * n. sintomi * Esito dopo


Tabella di contingenza	
Conteggio  	
Esito dopo	n. sintomi	Totale	
	0	1	2	3	4		
1	1 Sintomo	0	4	0	0	0	0	4	
		1	0	3	5	2	1	11	
		3	0	0	1	0	0	1	
		4	0	0	1	0	0	1	
		7	0	1	0	0	0	1	
		9	0	0	1	0	0	1	
	Totale	4	4	8	2	1	19	
2	1 Sintomo	0	17	0	0	0		17	
		1	0	1	3	8		12	
		4	0	3	0	0		3	
		7	0	2	0	0		2	
		11	0	1	0	0		1	
	Totale	17	7	3	8		35	
Totale	1 Sintomo	0	21	0	0	0	0	21	
		1	0	4	8	10	1	23	
		3	0	0	1	0	0	1	
		4	0	3	1	0	0	4	
		7	0	3	0	0	0	3	
		9	0	0	1	0	0	1	
		11	0	1	0	0	0	1	
	Totale	21	11	11	10	1	54	


Test del chi-quadrato	
Esito dopo	Valore	df	Significatività asintotica (bilaterale)	
1	Chi-quadrato di Pearson	26,341b	20	,155	
	Rapporto di verosimiglianza	26,369	20	,154	
	Associazione lineare per lineare	,523	1	,470	
	N di casi validi	19			
2	Chi-quadrato di Pearson	62,500c	12	,000	
	Rapporto di verosimiglianza	65,665	12	,000	
	Associazione lineare per lineare	,817	1	,366	
	N di casi validi	35			
Totale	Chi-quadrato di Pearson	84,628a	24	,000	
	Rapporto di verosimiglianza	93,063	24	,000	
	Associazione lineare per lineare	1,565	1	,211	
	N di casi validi	54			

a. 33 celle (94,3%) hanno un conteggio previsto inferiore a 5. Il conteggio previsto minimo è ,02.	
b. 30 celle (100,0%) hanno un conteggio previsto inferiore a 5. Il conteggio previsto minimo è ,05.	
c. 18 celle (90,0%) hanno un conteggio previsto inferiore a 5. Il conteggio previsto minimo è ,09.	


Misure simmetriche	
Esito dopo	Valore	Errore standard asintoticoa	
1	Intervallo per intervallo	R di Pearson	,170	,150	
	Ordinale per ordinale	Correlazione di Spearman	,519	,218	
	N di casi validi	19		
2	Intervallo per intervallo	R di Pearson	,155	,076	
	Ordinale per ordinale	Correlazione di Spearman	,780	,101	
	N di casi validi	35		
Totale	Intervallo per intervallo	R di Pearson	,172	,079	
	Ordinale per ordinale	Correlazione di Spearman	,689	,102	
	N di casi validi	54		

Misure simmetriche	
Esito dopo	T approssimatob	Significatività approssimata	
1	Intervallo per intervallo	R di Pearson	,713	,485c	
	Ordinale per ordinale	Correlazione di Spearman	2,506	,023c	
	N di casi validi			
2	Intervallo per intervallo	R di Pearson	,902	,374c	
	Ordinale per ordinale	Correlazione di Spearman	7,154	,000c	
	N di casi validi			
Totale	Intervallo per intervallo	R di Pearson	1,258	,214c	
	Ordinale per ordinale	Correlazione di Spearman	6,861	,000c	
	N di casi validi			

a. Non viene assunta l'ipotesi nulla.	
b. Viene utilizzato l'errore standard asintotico presumendo l'ipotesi nulla.	
c. Basato sull'approssimazione normale.	


2 Sintomo * n. sintomi * Esito dopo


Tabella di contingenza	
Conteggio  	
Esito dopo	n. sintomi	Totale	
	2	3	4		
1	2 Sintomo	3	1	0	0	1	
		4	1	0	0	1	
		7	3	0	0	3	
		8	1	0	0	1	
		9	2	2	1	5	
	Totale	8	2	1	11	
2	2 Sintomo	4	1	0		1	
		7	1	0		1	
		8	0	2		2	
		9	1	5		6	
		10	0	1		1	
	Totale	3	8		11	
Totale	2 Sintomo	3	1	0	0	1	
		4	2	0	0	2	
		7	4	0	0	4	
		8	1	2	0	3	
		9	3	7	1	11	
		10	0	1	0	1	
	Totale	11	10	1	22	


Test del chi-quadrato	
Esito dopo	Valore	df	Significatività asintotica (bilaterale)	
1	Chi-quadrato di Pearson	4,950b	8	,763	
	Rapporto di verosimiglianza	6,161	8	,629	
	Associazione lineare per lineare	2,116	1	,146	
	N di casi validi	11			
2	Chi-quadrato di Pearson	6,799c	4	,147	
	Rapporto di verosimiglianza	7,484	4	,112	
	Associazione lineare per lineare	4,064	1	,044	
	N di casi validi	11			
Totale	Chi-quadrato di Pearson	11,236a	10	,339	
	Rapporto di verosimiglianza	14,462	10	,153	
	Associazione lineare per lineare	6,503	1	,011	
	N di casi validi	22			

a. 16 celle (88,9%) hanno un conteggio previsto inferiore a 5. Il conteggio previsto minimo è ,05.	
b. 15 celle (100,0%) hanno un conteggio previsto inferiore a 5. Il conteggio previsto minimo è ,09.	
c. 10 celle (100,0%) hanno un conteggio previsto inferiore a 5. Il conteggio previsto minimo è ,27.	


Misure simmetriche	
Esito dopo	Valore	Errore standard asintoticoa	
1	Intervallo per intervallo	R di Pearson	,460	,116	
	Ordinale per ordinale	Correlazione di Spearman	,609	,147	
	N di casi validi	11		
2	Intervallo per intervallo	R di Pearson	,637	,196	
	Ordinale per ordinale	Correlazione di Spearman	,529	,260	
	N di casi validi	11		
Totale	Intervallo per intervallo	R di Pearson	,556	,092	
	Ordinale per ordinale	Correlazione di Spearman	,631	,124	
	N di casi validi	22		

Misure simmetriche	
Esito dopo	T approssimatob	Significatività approssimata	
1	Intervallo per intervallo	R di Pearson	1,554	,155c	
	Ordinale per ordinale	Correlazione di Spearman	2,301	,047c	
	N di casi validi			
2	Intervallo per intervallo	R di Pearson	2,482	,035c	
	Ordinale per ordinale	Correlazione di Spearman	1,872	,094c	
	N di casi validi			
Totale	Intervallo per intervallo	R di Pearson	2,995	,007c	
	Ordinale per ordinale	Correlazione di Spearman	3,640	,002c	
	N di casi validi			

a. Non viene assunta l'ipotesi nulla.	
b. Viene utilizzato l'errore standard asintotico presumendo l'ipotesi nulla.	
c. Basato sull'approssimazione normale.	


3 Sintomo * n. sintomi * Esito dopo


Tabella di contingenza	
Conteggio  	
Esito dopo	n. sintomi	Totale	
	3	4		
1	3 Sintomo	2	0	1	1	
		7	2	0	2	
	Totale	2	1	3	
2	3 Sintomo	6	2		2	
		7	6		6	
	Totale	8		8	
Totale	3 Sintomo	2	0	1	1	
		6	2	0	2	
		7	8	0	8	
	Totale	10	1	11	


Test del chi-quadrato	
Esito dopo	Valore	df	Significatività asintotica (bilaterale)	Sign. esatta (bilaterale)	
1	Chi-quadrato di Pearson	3,000b	1	,083		
	Correzione di continuitàc	,188	1	,665		
	Rapporto di verosimiglianza	3,819	1	,051		
	Test esatto di Fisher				,333	
	Associazione lineare per lineare	2,000	1	,157		
	N di casi validi	3				
2	Chi-quadrato di Pearson	.d				
	N di casi validi	8				
Totale	Chi-quadrato di Pearson	11,000a	2	,004		
	Rapporto di verosimiglianza	6,702	2	,035		
	Associazione lineare per lineare	9,290	1	,002		
	N di casi validi	11				

Test del chi-quadrato	
Esito dopo	Sign. esatta (unilaterale)	
1	Chi-quadrato di Pearson		
	Correzione di continuitàc		
	Rapporto di verosimiglianza		
	Test esatto di Fisher	,333	
	Associazione lineare per lineare		
	N di casi validi		
2	Chi-quadrato di Pearson		
	N di casi validi		
Totale	Chi-quadrato di Pearson		
	Rapporto di verosimiglianza		
	Associazione lineare per lineare		
	N di casi validi		

a. 5 celle (83,3%) hanno un conteggio previsto inferiore a 5. Il conteggio previsto minimo è ,09.	
b. 4 celle (100,0%) hanno un conteggio previsto inferiore a 5. Il conteggio previsto minimo è ,33.	
c. Calcolato solo per una tabella 2x2	
d. Non viene calcolata alcuna statistica perché n. sintomi è una costante.	


Misure simmetriche	
Esito dopo	Valore	Errore standard asintoticoa	
1	Intervallo per intervallo	R di Pearson	-1,000	,000	
	Ordinale per ordinale	Correlazione di Spearman	-1,000	,000c	
	N di casi validi	3		
2	Intervallo per intervallo	R di Pearson	.d		
	N di casi validi	8		
Totale	Intervallo per intervallo	R di Pearson	-,964	,037	
	Ordinale per ordinale	Correlazione di Spearman	-,638	,234	
	N di casi validi	11		

Misure simmetriche	
Esito dopo	T approssimatob	Significatività approssimata	
1	Intervallo per intervallo	R di Pearson	-94906265,624	,000c	
	Ordinale per ordinale	Correlazione di Spearman			
	N di casi validi			
2	Intervallo per intervallo	R di Pearson			
	N di casi validi			
Totale	Intervallo per intervallo	R di Pearson	-10,854	,000c	
	Ordinale per ordinale	Correlazione di Spearman	-2,487	,035c	
	N di casi validi			

a. Non viene assunta l'ipotesi nulla.	
b. Viene utilizzato l'errore standard asintotico presumendo l'ipotesi nulla.	
c. Basato sull'approssimazione normale.	
d. Non viene calcolata alcuna statistica perché n. sintomi è una costante.	


4 Sintomo * n. sintomi * Esito dopo


Tabella di contingenza	
Conteggio  	
Esito dopo	n. sintomi	Totale	
	4		
1	4 Sintomo	7	1	1	
	Totale	1	1	
Totale	4 Sintomo	7	1	1	
	Totale	1	1	


Test del chi-quadrato	
Esito dopo	Valore	
1	Chi-quadrato di Pearson	.a	
	N di casi validi	1	
Totale	Chi-quadrato di Pearson	.a	
	N di casi validi	1	

a. Non viene calcolata alcuna statistica perché 4 Sintomo e n. sintomi sono costanti.	


Misure simmetriche	
Esito dopo	Valore	
1	Intervallo per intervallo	R di Pearson	.a	
	N di casi validi	1	
Totale	Intervallo per intervallo	R di Pearson	.a	
	N di casi validi	1	

a. Non viene calcolata alcuna statistica perché 4 Sintomo e n. sintomi sono costanti.	

CROSSTABS
  /TABLES=@1Sintomo @2Sintomo @3Sintomo @4Sintomo BY n.sintomi BY ESITOprima BY Esitodopo
  /FORMAT=AVALUE TABLES
  /STATISTICS=CHISQ CORR
  /CELLS=COUNT
  /COUNT ROUND CELL.


Tabelle di contingenza


Note	
Output creato	30-NOV-2020 12:40:55	
Commenti		
Input	Dataset attivo	Dataset1	
	Filtro	<nessuno>	
	Peso	<nessuno>	
	Suddividi file	<nessuno>	
	N di righe nel file di dati di lavoro	54	
Gestione valori mancanti	Definizione di mancante	I valori mancanti definiti dall'utente vengono trattati come mancanti.	
	Casi utilizzati	Le statistiche per ciascuna tabella sono basate su tutti i casi con dati validi nell'intervallo o negli intervalli specificati per tutte le variabili in ciascuna tabella.	
Sintassi	CROSSTABS
  /TABLES=@1Sintomo @2Sintomo @3Sintomo @4Sintomo BY n.sintomi BY ESITOprima BY Esitodopo
  /FORMAT=AVALUE TABLES
  /STATISTICS=CHISQ CORR
  /CELLS=COUNT
  /COUNT ROUND CELL.	
Risorse	Tempo processore	00:00:00,02	
	Tempo trascorso	00:00:00,02	
	Dimensioni richieste	4	
	Celle disponibili	393184	


Riepilogo elaborazione casi	
	Casi	
	Valido	Mancante	Totale	
	N	Percentuale	N	Percentuale	N	
1 Sintomo * n. sintomi * ESITO prima * Esito dopo	54	100,0%	0	0,0%	54	
2 Sintomo * n. sintomi * ESITO prima * Esito dopo	22	40,7%	32	59,3%	54	
3 Sintomo * n. sintomi * ESITO prima * Esito dopo	11	20,4%	43	79,6%	54	
4 Sintomo * n. sintomi * ESITO prima * Esito dopo	1	1,9%	53	98,1%	54	

Riepilogo elaborazione casi	
	Casi	
	Totale	
	Percentuale	
1 Sintomo * n. sintomi * ESITO prima * Esito dopo	100,0%	
2 Sintomo * n. sintomi * ESITO prima * Esito dopo	100,0%	
3 Sintomo * n. sintomi * ESITO prima * Esito dopo	100,0%	
4 Sintomo * n. sintomi * ESITO prima * Esito dopo	100,0%	


1 Sintomo * n. sintomi * ESITO prima * Esito dopo


Tabella di contingenza	
Conteggio  	
Esito dopo	ESITO prima	n. sintomi	Totale	
		0	1	2	3	4		
1	0	1 Sintomo	0	2	0	0			2	
			1	0	1	3			4	
			3	0	0	1			1	
			7	0	1	0			1	
			9	0	0	1			1	
		Totale	2	2	5			9	
	1	1 Sintomo	0	2	0	0	0	0	2	
			1	0	2	2	2	1	7	
			4	0	0	1	0	0	1	
		Totale	2	2	3	2	1	10	
	Totale	1 Sintomo	0	4	0	0	0	0	4	
			1	0	3	5	2	1	11	
			3	0	0	1	0	0	1	
			4	0	0	1	0	0	1	
			7	0	1	0	0	0	1	
			9	0	0	1	0	0	1	
		Totale	4	4	8	2	1	19	
2	0	1 Sintomo	0	16	0	0	0		16	
			1	0	1	1	5		7	
			4	0	3	0	0		3	
			7	0	2	0	0		2	
			11	0	1	0	0		1	
		Totale	16	7	1	5		29	
	1	1 Sintomo	0	1		0	0		1	
			1	0		2	3		5	
		Totale	1		2	3		6	
	Totale	1 Sintomo	0	17	0	0	0		17	
			1	0	1	3	8		12	
			4	0	3	0	0		3	
			7	0	2	0	0		2	
			11	0	1	0	0		1	
		Totale	17	7	3	8		35	
Totale	0	1 Sintomo	0	18	0	0	0		18	
			1	0	2	4	5		11	
			3	0	0	1	0		1	
			4	0	3	0	0		3	
			7	0	3	0	0		3	
			9	0	0	1	0		1	
			11	0	1	0	0		1	
		Totale	18	9	6	5		38	
	1	1 Sintomo	0	3	0	0	0	0	3	
			1	0	2	4	5	1	12	
			4	0	0	1	0	0	1	
		Totale	3	2	5	5	1	16	
	Totale	1 Sintomo	0	21	0	0	0	0	21	
			1	0	4	8	10	1	23	
			3	0	0	1	0	0	1	
			4	0	3	1	0	0	4	
			7	0	3	0	0	0	3	
			9	0	0	1	0	0	1	
			11	0	1	0	0	0	1	
		Totale	21	11	11	10	1	54	


Test del chi-quadrato	
Esito dopo	ESITO prima	Valore	df	Significatività asintotica (bilaterale)	
1	0	Chi-quadrato di Pearson	13,275e	8	,103	
		Rapporto di verosimiglianza	13,412	8	,098	
		Associazione lineare per lineare	,851	1	,356	
		N di casi validi	9			
	1	Chi-quadrato di Pearson	12,381f	8	,135	
		Rapporto di verosimiglianza	12,217	8	,142	
		Associazione lineare per lineare	,934	1	,334	
		N di casi validi	10			
	Totale	Chi-quadrato di Pearson	26,341d	20	,155	
		Rapporto di verosimiglianza	26,369	20	,154	
		Associazione lineare per lineare	,523	1	,470	
		N di casi validi	19			
2	0	Chi-quadrato di Pearson	50,306h	12	,000	
		Rapporto di verosimiglianza	52,095	12	,000	
		Associazione lineare per lineare	1,292	1	,256	
		N di casi validi	29			
	1	Chi-quadrato di Pearson	6,000i	2	,050	
		Rapporto di verosimiglianza	5,407	2	,067	
		Associazione lineare per lineare	4,122	1	,042	
		N di casi validi	6			
	Totale	Chi-quadrato di Pearson	62,500g	12	,000	
		Rapporto di verosimiglianza	65,665	12	,000	
		Associazione lineare per lineare	,817	1	,366	
		N di casi validi	35			
Totale	0	Chi-quadrato di Pearson	70,242b	18	,000	
		Rapporto di verosimiglianza	72,461	18	,000	
		Associazione lineare per lineare	2,459	1	,117	
		N di casi validi	38			
	1	Chi-quadrato di Pearson	18,133c	8	,020	
		Rapporto di verosimiglianza	17,489	8	,025	
		Associazione lineare per lineare	1,962	1	,161	
		N di casi validi	16			
	Totale	Chi-quadrato di Pearson	84,628a	24	,000	
		Rapporto di verosimiglianza	93,063	24	,000	
		Associazione lineare per lineare	1,565	1	,211	
		N di casi validi	54			

a. 33 celle (94,3%) hanno un conteggio previsto inferiore a 5. Il conteggio previsto minimo è ,02.	
b. 26 celle (92,9%) hanno un conteggio previsto inferiore a 5. Il conteggio previsto minimo è ,13.	
c. 15 celle (100,0%) hanno un conteggio previsto inferiore a 5. Il conteggio previsto minimo è ,06.	
d. 30 celle (100,0%) hanno un conteggio previsto inferiore a 5. Il conteggio previsto minimo è ,05.	
e. 15 celle (100,0%) hanno un conteggio previsto inferiore a 5. Il conteggio previsto minimo è ,22.	
f. 15 celle (100,0%) hanno un conteggio previsto inferiore a 5. Il conteggio previsto minimo è ,10.	
g. 18 celle (90,0%) hanno un conteggio previsto inferiore a 5. Il conteggio previsto minimo è ,09.	
h. 19 celle (95,0%) hanno un conteggio previsto inferiore a 5. Il conteggio previsto minimo è ,03.	
i. 6 celle (100,0%) hanno un conteggio previsto inferiore a 5. Il conteggio previsto minimo è ,17.	


Misure simmetriche	
Esito dopo	ESITO prima	Valore	Errore standard asintoticoa	T approssimatob	
1	0	Intervallo per intervallo	R di Pearson	,326	,223	,913	
		Ordinale per ordinale	Correlazione di Spearman	,576	,299	1,863	
		N di casi validi	9			
	1	Intervallo per intervallo	R di Pearson	,322	,180	,962	
		Ordinale per ordinale	Correlazione di Spearman	,582	,260	2,023	
		N di casi validi	10			
	Totale	Intervallo per intervallo	R di Pearson	,170	,150	,713	
		Ordinale per ordinale	Correlazione di Spearman	,519	,218	2,506	
		N di casi validi	19			
2	0	Intervallo per intervallo	R di Pearson	,215	,082	1,143	
		Ordinale per ordinale	Correlazione di Spearman	,840	,085	8,048	
		N di casi validi	29			
	1	Intervallo per intervallo	R di Pearson	,908	,078	4,333	
		Ordinale per ordinale	Correlazione di Spearman	,707	,228	2,000	
		N di casi validi	6			
	Totale	Intervallo per intervallo	R di Pearson	,155	,076	,902	
		Ordinale per ordinale	Correlazione di Spearman	,780	,101	7,154	
		N di casi validi	35			
Totale	0	Intervallo per intervallo	R di Pearson	,258	,095	1,601	
		Ordinale per ordinale	Correlazione di Spearman	,770	,097	7,239	
		N di casi validi	38			
	1	Intervallo per intervallo	R di Pearson	,362	,181	1,451	
		Ordinale per ordinale	Correlazione di Spearman	,575	,219	2,628	
		N di casi validi	16			
	Totale	Intervallo per intervallo	R di Pearson	,172	,079	1,258	
		Ordinale per ordinale	Correlazione di Spearman	,689	,102	6,861	
		N di casi validi	54			

Misure simmetriche	
Esito dopo	ESITO prima	Significatività approssimata	
1	0	Intervallo per intervallo	R di Pearson	,392c	
		Ordinale per ordinale	Correlazione di Spearman	,105c	
		N di casi validi		
	1	Intervallo per intervallo	R di Pearson	,364c	
		Ordinale per ordinale	Correlazione di Spearman	,078c	
		N di casi validi		
	Totale	Intervallo per intervallo	R di Pearson	,485c	
		Ordinale per ordinale	Correlazione di Spearman	,023c	
		N di casi validi		
2	0	Intervallo per intervallo	R di Pearson	,263c	
		Ordinale per ordinale	Correlazione di Spearman	,000c	
		N di casi validi		
	1	Intervallo per intervallo	R di Pearson	,012c	
		Ordinale per ordinale	Correlazione di Spearman	,116c	
		N di casi validi		
	Totale	Intervallo per intervallo	R di Pearson	,374c	
		Ordinale per ordinale	Correlazione di Spearman	,000c	
		N di casi validi		
Totale	0	Intervallo per intervallo	R di Pearson	,118c	
		Ordinale per ordinale	Correlazione di Spearman	,000c	
		N di casi validi		
	1	Intervallo per intervallo	R di Pearson	,169c	
		Ordinale per ordinale	Correlazione di Spearman	,020c	
		N di casi validi		
	Totale	Intervallo per intervallo	R di Pearson	,214c	
		Ordinale per ordinale	Correlazione di Spearman	,000c	
		N di casi validi		

a. Non viene assunta l'ipotesi nulla.	
b. Viene utilizzato l'errore standard asintotico presumendo l'ipotesi nulla.	
c. Basato sull'approssimazione normale.	


2 Sintomo * n. sintomi * ESITO prima * Esito dopo


Tabella di contingenza	
Conteggio  	
Esito dopo	ESITO prima	n. sintomi	Totale	
		2	3	4		
1	0	2 Sintomo	7	2			2	
			8	1			1	
			9	2			2	
		Totale	5			5	
	1	2 Sintomo	3	1	0	0	1	
			4	1	0	0	1	
			7	1	0	0	1	
			9	0	2	1	3	
		Totale	3	2	1	6	
	Totale	2 Sintomo	3	1	0	0	1	
			4	1	0	0	1	
			7	3	0	0	3	
			8	1	0	0	1	
			9	2	2	1	5	
		Totale	8	2	1	11	
2	0	2 Sintomo	4	1	0		1	
			8	0	1		1	
			9	0	3		3	
			10	0	1		1	
		Totale	1	5		6	
	1	2 Sintomo	7	1	0		1	
			8	0	1		1	
			9	1	2		3	
		Totale	2	3		5	
	Totale	2 Sintomo	4	1	0		1	
			7	1	0		1	
			8	0	2		2	
			9	1	5		6	
			10	0	1		1	
		Totale	3	8		11	
Totale	0	2 Sintomo	4	1	0		1	
			7	2	0		2	
			8	1	1		2	
			9	2	3		5	
			10	0	1		1	
		Totale	6	5		11	
	1	2 Sintomo	3	1	0	0	1	
			4	1	0	0	1	
			7	2	0	0	2	
			8	0	1	0	1	
			9	1	4	1	6	
		Totale	5	5	1	11	
	Totale	2 Sintomo	3	1	0	0	1	
			4	2	0	0	2	
			7	4	0	0	4	
			8	1	2	0	3	
			9	3	7	1	11	
			10	0	1	0	1	
		Totale	11	10	1	22	


Test del chi-quadrato	
Esito dopo	ESITO prima	Valore	df	Significatività asintotica (bilaterale)	
1	0	Chi-quadrato di Pearson	.d			
		N di casi validi	5			
	1	Chi-quadrato di Pearson	6,000e	6	,423	
		Rapporto di verosimiglianza	8,318	6	,216	
		Associazione lineare per lineare	3,059	1	,080	
		N di casi validi	6			
	Totale	Chi-quadrato di Pearson	4,950c	8	,763	
		Rapporto di verosimiglianza	6,161	8	,629	
		Associazione lineare per lineare	2,116	1	,146	
		N di casi validi	11			
2	0	Chi-quadrato di Pearson	6,000g	3	,112	
		Rapporto di verosimiglianza	5,407	3	,144	
		Associazione lineare per lineare	4,562	1	,033	
		N di casi validi	6			
	1	Chi-quadrato di Pearson	2,222h	2	,329	
		Rapporto di verosimiglianza	2,911	2	,233	
		Associazione lineare per lineare	,667	1	,414	
		N di casi validi	5			
	Totale	Chi-quadrato di Pearson	6,799f	4	,147	
		Rapporto di verosimiglianza	7,484	4	,112	
		Associazione lineare per lineare	4,064	1	,044	
		N di casi validi	11			
Totale	0	Chi-quadrato di Pearson	4,143b	4	,387	
		Rapporto di verosimiglianza	5,655	4	,226	
		Associazione lineare per lineare	2,815	1	,093	
		N di casi validi	11			
	1	Chi-quadrato di Pearson	8,067c	8	,427	
		Rapporto di verosimiglianza	10,154	8	,254	
		Associazione lineare per lineare	3,969	1	,046	
		N di casi validi	11			
	Totale	Chi-quadrato di Pearson	11,236a	10	,339	
		Rapporto di verosimiglianza	14,462	10	,153	
		Associazione lineare per lineare	6,503	1	,011	
		N di casi validi	22			

a. 16 celle (88,9%) hanno un conteggio previsto inferiore a 5. Il conteggio previsto minimo è ,05.	
b. 10 celle (100,0%) hanno un conteggio previsto inferiore a 5. Il conteggio previsto minimo è ,45.	
c. 15 celle (100,0%) hanno un conteggio previsto inferiore a 5. Il conteggio previsto minimo è ,09.	
d. Non viene calcolata alcuna statistica perché n. sintomi è una costante.	
e. 12 celle (100,0%) hanno un conteggio previsto inferiore a 5. Il conteggio previsto minimo è ,17.	
f. 10 celle (100,0%) hanno un conteggio previsto inferiore a 5. Il conteggio previsto minimo è ,27.	
g. 8 celle (100,0%) hanno un conteggio previsto inferiore a 5. Il conteggio previsto minimo è ,17.	
h. 6 celle (100,0%) hanno un conteggio previsto inferiore a 5. Il conteggio previsto minimo è ,40.	


Misure simmetriche	
Esito dopo	ESITO prima	Valore	Errore standard asintoticoa	
1	0	Intervallo per intervallo	R di Pearson	.d		
		N di casi validi	5		
	1	Intervallo per intervallo	R di Pearson	,782	,074	
		Ordinale per ordinale	Correlazione di Spearman	,885	,024	
		N di casi validi	6		
	Totale	Intervallo per intervallo	R di Pearson	,460	,116	
		Ordinale per ordinale	Correlazione di Spearman	,609	,147	
		N di casi validi	11		
2	0	Intervallo per intervallo	R di Pearson	,955	,045	
		Ordinale per ordinale	Correlazione di Spearman	,696	,235	
		N di casi validi	6		
	1	Intervallo per intervallo	R di Pearson	,408	,394	
		Ordinale per ordinale	Correlazione di Spearman	,323	,459	
		N di casi validi	5		
	Totale	Intervallo per intervallo	R di Pearson	,637	,196	
		Ordinale per ordinale	Correlazione di Spearman	,529	,260	
		N di casi validi	11		
Totale	0	Intervallo per intervallo	R di Pearson	,531	,142	
		Ordinale per ordinale	Correlazione di Spearman	,578	,189	
		N di casi validi	11		
	1	Intervallo per intervallo	R di Pearson	,630	,121	
		Ordinale per ordinale	Correlazione di Spearman	,714	,163	
		N di casi validi	11		
	Totale	Intervallo per intervallo	R di Pearson	,556	,092	
		Ordinale per ordinale	Correlazione di Spearman	,631	,124	
		N di casi validi	22		

Misure simmetriche	
Esito dopo	ESITO prima	T approssimatob	Significatività approssimata	
1	0	Intervallo per intervallo	R di Pearson			
		N di casi validi			
	1	Intervallo per intervallo	R di Pearson	2,511	,066c	
		Ordinale per ordinale	Correlazione di Spearman	3,809	,019c	
		N di casi validi			
	Totale	Intervallo per intervallo	R di Pearson	1,554	,155c	
		Ordinale per ordinale	Correlazione di Spearman	2,301	,047c	
		N di casi validi			
2	0	Intervallo per intervallo	R di Pearson	6,455	,003c	
		Ordinale per ordinale	Correlazione di Spearman	1,936	,125c	
		N di casi validi			
	1	Intervallo per intervallo	R di Pearson	,775	,495c	
		Ordinale per ordinale	Correlazione di Spearman	,591	,596c	
		N di casi validi			
	Totale	Intervallo per intervallo	R di Pearson	2,482	,035c	
		Ordinale per ordinale	Correlazione di Spearman	1,872	,094c	
		N di casi validi			
Totale	0	Intervallo per intervallo	R di Pearson	1,878	,093c	
		Ordinale per ordinale	Correlazione di Spearman	2,126	,062c	
		N di casi validi			
	1	Intervallo per intervallo	R di Pearson	2,434	,038c	
		Ordinale per ordinale	Correlazione di Spearman	3,062	,014c	
		N di casi validi			
	Totale	Intervallo per intervallo	R di Pearson	2,995	,007c	
		Ordinale per ordinale	Correlazione di Spearman	3,640	,002c	
		N di casi validi			

a. Non viene assunta l'ipotesi nulla.	
b. Viene utilizzato l'errore standard asintotico presumendo l'ipotesi nulla.	
c. Basato sull'approssimazione normale.	
d. Non viene calcolata alcuna statistica perché n. sintomi è una costante.	


3 Sintomo * n. sintomi * ESITO prima * Esito dopo


Tabella di contingenza	
Conteggio  	
Esito dopo	ESITO prima	n. sintomi	Totale	
		3	4		
1	1	3 Sintomo	2	0	1	1	
			7	2	0	2	
		Totale	2	1	3	
	Totale	3 Sintomo	2	0	1	1	
			7	2	0	2	
		Totale	2	1	3	
2	0	3 Sintomo	6	1		1	
			7	4		4	
		Totale	5		5	
	1	3 Sintomo	6	1		1	
			7	2		2	
		Totale	3		3	
	Totale	3 Sintomo	6	2		2	
			7	6		6	
		Totale	8		8	
Totale	0	3 Sintomo	6	1		1	
			7	4		4	
		Totale	5		5	
	1	3 Sintomo	2	0	1	1	
			6	1	0	1	
			7	4	0	4	
		Totale	5	1	6	
	Totale	3 Sintomo	2	0	1	1	
			6	2	0	2	
			7	8	0	8	
		Totale	10	1	11	


Test del chi-quadrato	
Esito dopo	ESITO prima	Valore	df	Significatività asintotica (bilaterale)	Sign. esatta (bilaterale)	Sign. esatta (unilaterale)	
1	1	Chi-quadrato di Pearson	3,000d	1	,083			
		Correzione di continuitàe	,188	1	,665			
		Rapporto di verosimiglianza	3,819	1	,051			
		Test esatto di Fisher				,333	,333	
		Associazione lineare per lineare	2,000	1	,157			
		N di casi validi	3					
	Totale	Chi-quadrato di Pearson	3,000d	1	,083			
		Correzione di continuitàe	,188	1	,665			
		Rapporto di verosimiglianza	3,819	1	,051			
		Test esatto di Fisher				,333	,333	
		Associazione lineare per lineare	2,000	1	,157			
		N di casi validi	3					
2	0	Chi-quadrato di Pearson	.b					
		N di casi validi	5					
	1	Chi-quadrato di Pearson	.b					
		N di casi validi	3					
	Totale	Chi-quadrato di Pearson	.b					
		N di casi validi	8					
Totale	0	Chi-quadrato di Pearson	.b					
		N di casi validi	5					
	1	Chi-quadrato di Pearson	6,000c	2	,050			
		Rapporto di verosimiglianza	5,407	2	,067			
		Associazione lineare per lineare	4,800	1	,028			
		N di casi validi	6					
	Totale	Chi-quadrato di Pearson	11,000a	2	,004			
		Rapporto di verosimiglianza	6,702	2	,035			
		Associazione lineare per lineare	9,290	1	,002			
		N di casi validi	11					

a. 5 celle (83,3%) hanno un conteggio previsto inferiore a 5. Il conteggio previsto minimo è ,09.	
b. Non viene calcolata alcuna statistica perché n. sintomi è una costante.	
c. 6 celle (100,0%) hanno un conteggio previsto inferiore a 5. Il conteggio previsto minimo è ,17.	
d. 4 celle (100,0%) hanno un conteggio previsto inferiore a 5. Il conteggio previsto minimo è ,33.	
e. Calcolato solo per una tabella 2x2	


Misure simmetriche	
Esito dopo	ESITO prima	Valore	Errore standard asintoticoa	
1	1	Intervallo per intervallo	R di Pearson	-1,000	,000	
		Ordinale per ordinale	Correlazione di Spearman	-1,000	,000c	
		N di casi validi	3		
	Totale	Intervallo per intervallo	R di Pearson	-1,000	,000	
		Ordinale per ordinale	Correlazione di Spearman	-1,000	,000c	
		N di casi validi	3		
2	0	Intervallo per intervallo	R di Pearson	.d		
		N di casi validi	5		
	1	Intervallo per intervallo	R di Pearson	.d		
		N di casi validi	3		
	Totale	Intervallo per intervallo	R di Pearson	.d		
		N di casi validi	8		
Totale	0	Intervallo per intervallo	R di Pearson	.d		
		N di casi validi	5		
	1	Intervallo per intervallo	R di Pearson	-,980	,023	
		Ordinale per ordinale	Correlazione di Spearman	-,775	,224	
		N di casi validi	6		
	Totale	Intervallo per intervallo	R di Pearson	-,964	,037	
		Ordinale per ordinale	Correlazione di Spearman	-,638	,234	
		N di casi validi	11		

Misure simmetriche	
Esito dopo	ESITO prima	T approssimatob	
1	1	Intervallo per intervallo	R di Pearson	-94906265,624	
		Ordinale per ordinale	Correlazione di Spearman		
		N di casi validi		
	Totale	Intervallo per intervallo	R di Pearson	-94906265,624	
		Ordinale per ordinale	Correlazione di Spearman		
		N di casi validi		
2	0	Intervallo per intervallo	R di Pearson		
		N di casi validi		
	1	Intervallo per intervallo	R di Pearson		
		N di casi validi		
	Totale	Intervallo per intervallo	R di Pearson		
		N di casi validi		
Totale	0	Intervallo per intervallo	R di Pearson		
		N di casi validi		
	1	Intervallo per intervallo	R di Pearson	-9,798	
		Ordinale per ordinale	Correlazione di Spearman	-2,449	
		N di casi validi		
	Totale	Intervallo per intervallo	R di Pearson	-10,854	
		Ordinale per ordinale	Correlazione di Spearman	-2,487	
		N di casi validi		

Misure simmetriche	
Esito dopo	ESITO prima	Significatività approssimata	
1	1	Intervallo per intervallo	R di Pearson	,000c	
		Ordinale per ordinale	Correlazione di Spearman		
		N di casi validi		
	Totale	Intervallo per intervallo	R di Pearson	,000c	
		Ordinale per ordinale	Correlazione di Spearman		
		N di casi validi		
2	0	Intervallo per intervallo	R di Pearson		
		N di casi validi		
	1	Intervallo per intervallo	R di Pearson		
		N di casi validi		
	Totale	Intervallo per intervallo	R di Pearson		
		N di casi validi		
Totale	0	Intervallo per intervallo	R di Pearson		
		N di casi validi		
	1	Intervallo per intervallo	R di Pearson	,001c	
		Ordinale per ordinale	Correlazione di Spearman	,070c	
		N di casi validi		
	Totale	Intervallo per intervallo	R di Pearson	,000c	
		Ordinale per ordinale	Correlazione di Spearman	,035c	
		N di casi validi		

a. Non viene assunta l'ipotesi nulla.	
b. Viene utilizzato l'errore standard asintotico presumendo l'ipotesi nulla.	
c. Basato sull'approssimazione normale.	
d. Non viene calcolata alcuna statistica perché n. sintomi è una costante.	


4 Sintomo * n. sintomi * ESITO prima * Esito dopo


Tabella di contingenza	
Conteggio  	
Esito dopo	ESITO prima	n. sintomi	Totale	
		4		
1	1	4 Sintomo	7	1	1	
		Totale	1	1	
	Totale	4 Sintomo	7	1	1	
		Totale	1	1	
Totale	1	4 Sintomo	7	1	1	
		Totale	1	1	
	Totale	4 Sintomo	7	1	1	
		Totale	1	1	


Test del chi-quadrato	
Esito dopo	ESITO prima	Valore	
1	1	Chi-quadrato di Pearson	.a	
		N di casi validi	1	
	Totale	Chi-quadrato di Pearson	.a	
		N di casi validi	1	
Totale	1	Chi-quadrato di Pearson	.a	
		N di casi validi	1	
	Totale	Chi-quadrato di Pearson	.a	
		N di casi validi	1	

a. Non viene calcolata alcuna statistica perché 4 Sintomo e n. sintomi sono costanti.	


Misure simmetriche	
Esito dopo	ESITO prima	Valore	
1	1	Intervallo per intervallo	R di Pearson	.a	
		N di casi validi	1	
	Totale	Intervallo per intervallo	R di Pearson	.a	
		N di casi validi	1	
Totale	1	Intervallo per intervallo	R di Pearson	.a	
		N di casi validi	1	
	Totale	Intervallo per intervallo	R di Pearson	.a	
		N di casi validi	1	

a. Non viene calcolata alcuna statistica perché 4 Sintomo e n. sintomi sono costanti.	

CROSSTABS
  /TABLES=@1Sintomo @2Sintomo @3Sintomo @4Sintomo BY n.sintomi BY Esitodopo
  /SHOWDIM=2
  /FORMAT=NOTABLES
  /STATISTICS=CHISQ CORR
  /COUNT ROUND CELL
  /BARCHART.


Tabelle di contingenza


Note	
Output creato	30-NOV-2020 12:43:43	
Commenti		
Input	Dataset attivo	Dataset1	
	Filtro	<nessuno>	
	Peso	<nessuno>	
	Suddividi file	<nessuno>	
	N di righe nel file di dati di lavoro	54	
Gestione valori mancanti	Definizione di mancante	I valori mancanti definiti dall'utente vengono trattati come mancanti.	
	Casi utilizzati	Le statistiche per ciascuna tabella sono basate su tutti i casi con dati validi nell'intervallo o negli intervalli specificati per tutte le variabili in ciascuna tabella.	
Sintassi	CROSSTABS
  /TABLES=@1Sintomo @2Sintomo @3Sintomo @4Sintomo BY n.sintomi BY Esitodopo
  /SHOWDIM=2
  /FORMAT=NOTABLES
  /STATISTICS=CHISQ CORR
  /COUNT ROUND CELL
  /BARCHART.	
Risorse	Tempo processore	00:00:02,28	
	Tempo trascorso	00:00:01,62	
	Dimensioni richieste	3	
	Celle disponibili	449353	


Riepilogo elaborazione casi	
	Casi	
	Valido	Mancante	Totale	
	N	Percentuale	N	Percentuale	N	
1 Sintomo * n. sintomi * Esito dopo	54	100,0%	0	0,0%	54	
2 Sintomo * n. sintomi * Esito dopo	22	40,7%	32	59,3%	54	
3 Sintomo * n. sintomi * Esito dopo	11	20,4%	43	79,6%	54	
4 Sintomo * n. sintomi * Esito dopo	1	1,9%	53	98,1%	54	

Riepilogo elaborazione casi	
	Casi	
	Totale	
	Percentuale	
1 Sintomo * n. sintomi * Esito dopo	100,0%	
2 Sintomo * n. sintomi * Esito dopo	100,0%	
3 Sintomo * n. sintomi * Esito dopo	100,0%	
4 Sintomo * n. sintomi * Esito dopo	100,0%	


1 Sintomo * n. sintomi * Esito dopo


Test del chi-quadrato	
Esito dopo	Valore	df	Significatività asintotica (bilaterale)	
1	Chi-quadrato di Pearson	26,341b	20	,155	
	Rapporto di verosimiglianza	26,369	20	,154	
	Associazione lineare per lineare	,523	1	,470	
	N di casi validi	19			
2	Chi-quadrato di Pearson	62,500c	12	,000	
	Rapporto di verosimiglianza	65,665	12	,000	
	Associazione lineare per lineare	,817	1	,366	
	N di casi validi	35			
Totale	Chi-quadrato di Pearson	84,628a	24	,000	
	Rapporto di verosimiglianza	93,063	24	,000	
	Associazione lineare per lineare	1,565	1	,211	
	N di casi validi	54			

a. 33 celle (94,3%) hanno un conteggio previsto inferiore a 5. Il conteggio previsto minimo è ,02.	
b. 30 celle (100,0%) hanno un conteggio previsto inferiore a 5. Il conteggio previsto minimo è ,05.	
c. 18 celle (90,0%) hanno un conteggio previsto inferiore a 5. Il conteggio previsto minimo è ,09.	


Misure simmetriche	
Esito dopo	Valore	Errore standard asintoticoa	
1	Intervallo per intervallo	R di Pearson	,170	,150	
	Ordinale per ordinale	Correlazione di Spearman	,519	,218	
	N di casi validi	19		
2	Intervallo per intervallo	R di Pearson	,155	,076	
	Ordinale per ordinale	Correlazione di Spearman	,780	,101	
	N di casi validi	35		
Totale	Intervallo per intervallo	R di Pearson	,172	,079	
	Ordinale per ordinale	Correlazione di Spearman	,689	,102	
	N di casi validi	54		

Misure simmetriche	
Esito dopo	T approssimatob	Significatività approssimata	
1	Intervallo per intervallo	R di Pearson	,713	,485c	
	Ordinale per ordinale	Correlazione di Spearman	2,506	,023c	
	N di casi validi			
2	Intervallo per intervallo	R di Pearson	,902	,374c	
	Ordinale per ordinale	Correlazione di Spearman	7,154	,000c	
	N di casi validi			
Totale	Intervallo per intervallo	R di Pearson	1,258	,214c	
	Ordinale per ordinale	Correlazione di Spearman	6,861	,000c	
	N di casi validi			

a. Non viene assunta l'ipotesi nulla.	
b. Viene utilizzato l'errore standard asintotico presumendo l'ipotesi nulla.	
c. Basato sull'approssimazione normale.	

ìzPö544,Z´¨°°0%%eØ°awÜqÇÁÛ|í1&¬®®.//ÏÎÎÎÈÈ(++«­­=Þ?ñÔSO;699yÔ¨QO?ýtc?k>þøã#GLJJ|yë=ïkc?ÁVBó-[¶Lö²èAÙ²©M-]º´Ãì«««ËÉÉi½177wûöíí÷¿nÝº>úD¯Öæ9¶ØûlILLlýåo¼ñF_;û&NnÕºÌo²àSË¾y[Ã+¯¼ÒÒÒ²|ùò°ß>ÛÒ¢EÂpÚ´iÛ?TRR.l.½ôÒpÑìÙ³ßùÐ%zW±÷¹fiii¸èÝwß+axÕUWèmøÄM ûøÉ¾I&õ¢¢¢%K¬X±¢©©éUÔ¦BaeeedXUUn"OÈE_~Ý´iSë]ÅÞOäÕÕÕáæÍÃpÈ!~m'ßÅ"ûÙÄ[öÅ¸BMMÍ1c¢aÔ¿ÿU«VuIIIaØÜÜÈ«±í÷¹fKKKëkFw?k¶¹4|I_+ûÙÈ¾c[¿~ýwÞùLâââ³¯   õ3ma%ÛïyèÐ¡á¢·Þz+2<cÝUìýD®ùúë¯Ga'a8hÐ ½ÿÇÉ@<d_ä9³G´¥¥%Ä_XÏÉÉiÿµ			a½®®®±±1+**Â°¤¤¤õïÕ-^¼¸ýþ¯½öÚpÑ¬Y³ÂÕÞyçÈïçEw?kN<yëÖ­;vì¸ì²ËZÿn_çoìdÐS²/Æ+W^yeíK,i_EÃËÊÊÂ°¶¶6ò^¨¼¼¼úúúö7 ²²2mÞ¹4ö~"["a]ÿÿùÎ|­ìd û>MMM·Ýv[¨ºâââ¯íkh_E+V¬6lX¸NIIIdËæÍËËË³²²ÒÓÓÃÊ-[wyæQ£FE>xï©§j[1ö¹æ5kF¾|Â	a½õ;d ûºz³@öÈ>Ù ûd²Ùì N²ï»ßýn]]» Î³ïïþîïÞ~ûmwì@öÈ>Ù ûdì²OöÈ>Ù ûdì@ö û þNçÚ°aCiiiZZZffæüùóÌ²â0ûµRQQafÙWZZZSSÓØØxë­·avv¶Aö@f_mmmdØÐÐ½÷63È>Ãìkii=ÙbfÙt¹"T]ë_G ûÏìðÁc¼ó(dßã?¾nhõêÕùe3fu¸|eÑbÓÄ¥ßýîw§$û$ ]4û®¿þú~8Fö½ôÒKïÐ­X±¢0ÀY;céâÒ=d²ïO§L>iÒ¤M6y7n¬]»vlîó®êpñØ="û"=ZUUuÞyçÉ>ÙÄsöEÊ/--MöÉ> >³ï¬³ÎÚ°aÃ#GÞxãY³fÉ>ÙÄgö½ôÒKcÇMKK+--­««² §ª;ýðH;uêÔSx&Ù'ûdßgÜg²OöÉ>d@wDdìCö-*,,LII6lØwÜqðàÁö1Yß°aCiiiZZZffæüùóÃ×F/jóg®ª««ËËË³³³322ÊÊÊjkkÛ4âªU«F<uêÔwß÷W¿úÕÈ#SSSgÌ±ûv5)ûd²àSâ¬M·-]ºôxÙ*­õ5#ÝªöÕÕÕåää´ÞÛ&æúôé½ôÜsÏÑ^zé¥²OöÉ>dÀ§,###<ú½òÊ+---Ë/ëùùùÇË¾ÒÒÒÆÆÆ[o½5³³³Ùg-ÃiÓ¦mÿPIII.°õo¼ñÆ=öDþÅ`ÁaøÈ#õpdìÈ>OÙ¤IÂ£_QQÑ%KV¬XÑÔÔô§Su»ì¾VÛÐÐ½÷>fpÃÊÊÊÈ°ªª*ÜúÊÍhnn·mÛô·ÝdìCö555cÆ¾ÀÚ¿ÿU«V/ûZZZ"Ã£GÆè³¤¤¤0IFÚ.11±õ9Òzxøðáãý£î#Ù'ûõë×ßyç3gÎÅÅÅ)°´~¶/¬aaaá1¯c(ûdìCö|j"ÏÌ=úè£---!þÂzNNÎf_BBBäuÛÆÆÆ0¬¨¨ÃÖ¿Û·xñbÙ'ûd²à¹òÊ+Û¼wÉ%'ÃËÊÊÂ°¶¶6òN¨¼¼¼úúzÙ'ûd²àijjºí¶ÛB·¥¤¤ík_;pàÀfß+öPRRÙ²yóæòòò¬¬¬ôôô°²eËÎtì²Ù²Oö!û@öÉ>dÈ>Ù'ûdÈ>Ù'ûÙìd²È>²Ù²Oö!û@öÉ>dÈ>Ùìø´åµ½:Ü/½¶¶ÖûÝØ§dÜòXÿïí±õ¹ðÀk®È>÷î3gNZZZVVÖ¼yóÞÿs(ûd² ³ïßøÆÒ¥K÷ïßßÔÔtÛm·ýõ_ÿµ9²ÙÙwñÅ¿ñÆõ­[·Î1ÃÊ>ÙìÃìËÍÍmjj¬8pà´ÓN3²Oö!ûâ0û=Y+aheìCöÄaö%$$´Î¾¤¤$s(ûd² ³/77÷ÀõC4ÈÊ>ÙìÃì<yòÖ­[#ë;wî2e9²ÙÙw÷Ýwõ«_mlllnnþÞ÷¾wÏ=÷CÙ'ûqõõõ3gÎÌÈÈHKK3gÎÞ½Í¡ìÈ>n`î:þ¼©þ&/²Ù²Oö!û@öÉ>dÈ>ÙìøÌüà?¼ìÊ+c/_¼áûö+d² þù½FîuÑE1~¹¹ÑwòìCötÛì5«×1ìaÃd²ÙÐ³oÿþýEEEfOöÉ>d@<gßÞ½gÏíVöÉ>d@gßùç_YYéVöÉ>d@gßêÕ«ÿxÖ÷+ûd² ¾³ïÏú`eìCöÈ>dìCöÈ>dìCöÈ>dìCöÈ>dì²à8iRÿ3Ï4aB¥_VÖ/¼ ûÈ>nìµ×^¨#+W®4QÈ>dÈ>ÙìÙ'û]yÈ>áâsÏyú´#b,9ý2Ú¿¥Ù'ûÝÉEÿï¼Ûs¦Ä~,¹í?ÀÙ'ûñ7o¾äK²²²PQQ±÷ns(ûd² ³oÌ1=ôÐ¾û.]zÝu×CÙ'ûq­íÙ³';;ÛÊ>ÙìóìÛµk×°aÃÌ¡ìÈ>8Ï¾þðwÝu9²ÙÏÙ·sçÎ+®¸¢¥¥ÅÊ>ÙìÛìµwíµ×Ö××@Ù'ûq[¶lùÒ¾´yóf³'ûd² n³oõêÕãÇß¾»©]ÑªU«yî²§¸1kbåôÑì+..îõqæPöuÍÍÍ£F²czàGË®»f^ìåæoÚ·o¹¢«gßßþíß~óß<^ö­X±â¿OkæWËåW?ûì³ÿÍûþ÷¿?²a'³ÏtqiëÖ­ÊÙ÷'[¶l3fÌ¡C¯¾úê'AJfNÚußëxÉ?ãç?ÿù¸0o£u2ûL<ýìû+®¸âgùã-ûl_äÍ8­¨ÿ÷vv¸õ¹c~ú%^äÙw7(æïÊ>ÙÀ¼¹su$=5µ¶¶Ö¥³¯uÿµß(ûdÓÎ;ïÙ¦ýþó×ÄX¦qÂBöÉ>ÙÐ³²¯¾¾þª«®êß¿zzúÕW_½cÇs(ûºÙ'û8Ñì»è¢þéþiÏn¿ýö3fCÙ'ûdìÃìkmïÞ½©©©æPöÉ>Ù'ûâ6û>øà÷Þï¾ûî»öÚkÍ¡ì²OöÄmöÍ3'===--í¥^2²OöÉ>Ù·Ù9rä[n3f9²OöÉ>xÎ¾`÷îÝIIIæPöÉ>Ù'ûâ<ûvíÚUTTdeì² ³oÜ¸q?ýéO[ZZöíÛ÷ùwß·9²OöÉ>8Ì¾ÚÚÚÙ³g§§§Üu×]6²OöÉ>ÙÐÌ+/÷7y²Oö²OöÉ>²OöÉ>²OöÉ>.äG÷ßuYYìåËûöí3WÈ>Ù'ûº±óÆO8§OÂc,ÓÒ°²Oötûì+étmBJeHÿ',dì=4ûþy±²OöÉ>ÙÿÙwùå²OöÉ>8Ï¾W^yåK_úÇXÙ'ûdìóì+++ûÍo~ã1VöÉ>Ù'ûâ9ûÞ|óÍ3gþñÄï1VöÉ>Ù'ûâ8ûæÍ÷_üBöÉ>Ù'ûd@<g_MMÍØ±cÿ÷Äï1VöÉ>Ù'ûâ5ûÊÊÊz9²OöÉ>8Ì¾ø=ÆÊ>Ù'ûd@wqÁ¹çÍÌ30ÆÚï^È>Ù'ûº±×^í¡¬ÒD!ûdìÙ'ûdìÙ'ûdìÙ'û ûd²dì²dìì²²Oö²OöÉ>²OöÉ>²OöÉ>²Ù²OöÉ>Ù²OöÉ>Ù²OöÉ>@öÉ>ÙÈ>Ù'ûÙ'ûdÈ>Ù'ûdÈ>Ù'ûdÈ>ÙìÙ'ûdìÙ'ûdìÙ'ûd ûdìdìì²dì²dìsÜÈ>²Ù²OöÉ>Ù²OöÉ>Ù²OöÉ>@öÉ>ÙÈ>Ù'ûÙ'ûdÈ>Ù'ûdÈ>ÙìÛ·o_¸páàÁ/^_/ûWÙWSSÛëãòòò¶nÝú	öVUU5iÒ¤1cÆ¬Y³FöÉ> «dß9sÂyº¤¤$[ssseeå´iÓÂòòòO°·ñãÇ?òÈ#zòÉ'C;Ê>ÙtìKOOçéíÛ·G·õ°%##ãï³¥¥%Ä_HÀöÙ÷üdíIÐo`Ag²/kÄ¤ûî»o-¹hÊ3Fìp;qâ7¿ùÍý;&K¯½öò g_vvö1³/lÿd;ljjÊÌÌìÝ»÷Ã?Ü>û^ýõÆ ã´Ó;9#/^µjU#ùã+ú³fu¸¤ååÝsÏ=cw2ûL,öíÛ§<èÞÙ7wîÜÈ¼ÿ÷y£/_ïEÞ®~t¸dvß÷yºwöUWWçää´yKGffææÍÿ/»ÿ%JMM²è*ÙÔÕÕ-Z´(???))©  àúë¯ß²eË'ÛÕ#^xá+VÌ=[öÉ> eß§hÃãÆKKK5kV¨IÙ'ûSs£+Çô©ß'ûÙ'ûdp²ï²8eÙw¼§úúõë7räÈoë[²Oö@Üf_ÔwÜ!ûdìnÛ¶m;ë¬³¦O^UUuðàÁÊÊÊÉ'_xáï¿ÿþ½÷ÞNáC²Oö@·Ï¾3f´ùãl[·n[®¾úêÈïJJJ²Oö@·Ï¾äääp®¯¯nÙ±cGäwûZZZdì'ÙwöÙgóôe]V]]èÐ¡·Þz+¬-gyæªU«ÂÊ!CdìÐí³ï'èÛ·o·q-+W®¬/]ºTöÉ>ÙÝ>û^xá+®0`@BBBAAÁ¾ð_~9lï×¯ßüùódìÙ÷²8eÙçãeì>®YöÉ>«ìóqÍ²Oö@È>×,ûdôìóqÍ²Oö@È>×,ûdôìóqÍ²Oö@È¾?ø¸fÙ'û dßgFöÉ>àTfßóÏ?_VV&Lxì±ÇdìWÙ÷óÿ¼O>ÑßêûÃGàüÀÈ>Ù'û ~²oÔ¨Qá<ýøãG³oùòåaå3Î²Oö@üd_RRR8OG>¢/rÂÞ³gOXIII²Oö@üdß9çÎÓ?ýéO#Ù·k×®nº)¬?^öÉ>ÙñO>ùä1?·ï'²Oö@üd_ðúë¯_sÍ5CMNNÎÏÏ///_¿~ýÉ¸¹²Oö§,û¢¿ÒÕØØXVV6útÙ'ûdÄsöÕÕÕ-©©©²OöÉ>èöÙ7uêÔ^1yæ²OöÉ>èöÙêçxÁ0|øð+WÊ>Ù'û ÛgßÇNüÕyZöÉ>àeß®]»ózvvïÞ½Û<í'ûdìøÉ¾ùóçïÕ^Ù'ûdÄOöeffóô×¿þõ£GÔ+ûdpÊ²¯_¿~á<½÷îÏàæÊ>Ù²ì9sf8OoÙ²EöÉ>Ùñ/¿ürrrò%[[ÛÒÒ"ûdìøÌ¾Ø,ûdìÙ'ûdìn%Ù'ûSÛ·o_¸páàÁ/^_/ûdì¸Ê¾ÜÜÜ6/ïæååmÝºUöÉ>ÙñsæÌ	çéªªªæææÊÊÊiÓ¦-ååå²OöÉ>ìKOOçéíÛ·G·õ°%##CöÉ>ÙñÙÙÙÇÌ¾°]öÉ>ÙñsçÎ¼È[YYéE^Ù'û n³¯ºº:''§Í[:2337oÞ,ûdìøÉ¾ ®®nÑ¢EùùùIII×_ýIú½²Oö§2û>3²Oö§ û^õÕ/¼°MáôéÓ«ªªdìÙW]]ÎÐS§Nm½=ÃÆAýîw¿²Oö@·Ï¾pz.**zñÅ[oþùç.ºå[dìÐí³/???ùË_¶¿èÉ'qÆ²OöÉ>èöÙNÏû÷ïoÑ¾ûÂE©©©²OöÉ>èöÙùã[·nmÑ¶mÛÂEYYY²OöÉ>èöÙùSõWÕþ¢¿ù¿	]|ñÅ²OöÉ>èöÙ÷è£Ósß¾CùUWW·´´:thÓ¦MwÞygØ.zðÁeìÐí³/¸å[zÇM7ÝôÁÈ>Ù'û ²/X³fÍüùóXXXxÕUWóí½²OöÉ>èÆÙ÷²²Oö²OöÉ>²OöÉ>²OöÉ>²Ï$û@ö2/¾øâøñãSSS'L°qãFÙ'ûøÌ¾³Î:kÕªUÍÍÍ?øÁF%ûdÙuðàÁìììöÙ÷ðÃ¯>	ú,èLöe¸à;ßùÎj>ÒÉìK?ýô%K]ÐÉìwô¹gÝåë_ýªè.Ú¿²ïþë¿þëúë¯o¿ýío÷~¶ïâgyf?édöeúíoÌÀâNfßu×]wÛg?Ñ´ØK¸Îu_øè.²¯­Ïþóöìñ"oÏ|÷nX6þüßþØK¸Îóæ¹ »fß;æÏ¿mÛ¶öÉ>Ù'û N²ï¿üåäÉwîÜyÌKeì'ÙWTTÔ«Ù'ûdÄgöÅ&ûdìÙ'ûd ûdìÙ'ûdìÙ'ûdìÙ'û@²dì²dì²dì²dì²dìì²dì²dì²dì²dì²dì²dì²dì²dìì²dì²dì²dì²dì²dì²dì²dì²dìì²dì²dì²dì²dì²dì²dì²dì²dìì²dì²dì²dì²dì²dì²dì²dì²dìì²dì²dì²dì²dì²dì²dì²dìî²Oö²OöÉ>²OöÉ>²OöÉ>²OöÉ>²OöÉ>²OöÉ>²OöÉ>²Oö²OöÉ>@öÉ>Ù²OöÉ>Ù²ïäª««ËËË²Oö@<gßc=6|øðpâ²Oö@<gßìÙ³7mÚ#û-[öÔIÐo`Ag²/kÄßúÖ·â#Ì¾´ÓO_´hÑ9ÙÌ¾3gv2û.:µdòäÌ¬YÙÿðÿÐEæmÄÐ3eåt¸åå?ôÐCññªk:3a¹ýöÛ»Ýwî¾At¸¾råJ)QE:sT5¤ÍÛK/½¤<ÇxéxÙWSSÓrtúÙ¾öÙ>ÒÙgû½÷ÞÇ,îdöUTTt2û*®¹&oàÀ®èñ£ý¨ëÌÛwr/íp9;§ î·ÇO¼9û§bVÚwÝuW·ûîÂÝyúé½ÊË;õïÜº©ÜôìÎü«u¯y;|ø°ò@ö@öy×¼m^äÙªîÚØË9Y=ôP×·ÎLE±¸?Þ.pÞí9S:¹é#Ã·ûîÂÝþÎü¤pÜº©¼Ìù	W3o ûdì²OöÉ>@öÉ>Ù'ûdì ûdì²OöÉ>Ù²ïÔ²OöÉ>Ù'ûdÈ>Ù'ûdì²²OöÉ>Ù'ûdÈ>Ù'ûdì²Oöì²OöÉ>Ù'ûdÈ>Ùç²OöÉ>Ù²OöÉ>Ù'ûdì ûdì²OöÉ>Ù²OöÉ>Ù'ûdì ûdì²OöÉ>Ù²OöÉ>Ù'ûdìdì²OöÉ>Ù²OöÉ>Ù'ûdì ûdì²OöÉ>Ù²OöÉ>Ù'ûdì ûdì²OöÉ>Ù²OöÉ>Ù'ûdì ûdì²OöÉ>Ù²OöÉ>Ù'ûdì ûdì²OöÉ>@öÉ>Ù'ûdì ûdì²OöÉ>Ù²OöÉ>Ù'ûdì ûdì²OöÉ>Ù²OöÉ>Ù'ûdì ûdì²OöÉ>Ù²OöÉ>Ù'ûdì ûdì²OöÉ>Ù²OöÉ>Ù'ûdìdì²OöÉ>Ù²OöÉ>Ù'ûdì ûdì²OöÉ>Ù²OöÉ>Ù'ûdì ûdì²OöÉ>Ù²OöÉ>Ù'ûdì ûdì²OöÉ>Ù²OöÉ>Ù'ûdì3o ûdì²OöÉ>@öÉ>Ù'ûdì ûdì²OöÉ>Ù²OöÉ>Ù'ûdì ûdì²OöÉ>Ù²OöÉ>Ù'ûdì ûdì²OöÉÙ²OöÉ>Ù'ûdì ûdì²OöÉ>Ó²OöÉ>Ù'ûdìdì²OöÉ>Ù²OöÉ>Ù'ûdì ûdì²OöÉ>Ù²OöÉ>Ù'ûdì ûdì²OöÉ>Ù²OöÉ>Ù'ûd| ûdì²OöÉ>Ù²OöÉ>Ù'ûdìdì²OöÉ>ÙÈ>Ù'ûdì²dì²OöÉ>Ù'û@öÉ>Ù'ûdì²dì²OöÉ>Ù'û@öÉ>Ù'ûdì²dì²OöÉ>dÈ>Ù'ûdì²Oöì²OöÉ>Ù'ûÙ'ûdì²Oö²OöÉ>Ù'ûdìÙ'ûdì²OöÉ>'ÑË/¿<dÈÄÄÄ1cÆ¬[·NöÉ>Ù'ûdìÙWVVöÏÿüÏMMM÷ÜsÏ9sdì²OöÉ>Ùñ÷î+;wî,**jsi(ÂÉ'O?	ú&¥$fåv¸ôIL¾à¦óää¾ýúu¸ôIH=ztßÞ²ûu¸¤&&g&%Ú/ö®3¬ðô¬´´Ô¾ûõM½$ôé3~üø.2oáìÌT$öé÷Ç[an^zBÇ³Ò7qÄÝî»w_8ø;ó3Ò71ñ$=¸uSI;ó3®Ö½æmÉ%ÊÙ÷'III|ðAX9räHbbâgöïîÚµëíNØ±c¦µÝ»ww~Þêêê:så°ÏÃ¿Ý9ÍÍÍûöíëÌ5Ã¿ÞMç-¾°÷u7ýÃØï.<y<ùÉæºwö¥¥¥=z4!ÝCñÃohhüW¯°°Ð=Ù7þüïÿûüîw¿;wî@|fßÚµk^|ñE÷@|f²Ùì|v~øááÃ'''sÎ9÷ß¿	illÌÏÏ7?þñÇý¯|åàÁ&$xðÁõêÑP½>®O>=y6/_>lØ°~ýúM>ý7Þð²¯«ûí·Gñì³ÏîÝ»wõêÕn]OÝ»w_vÙe=üÔüë¿þë3¶mÛ&ä/þâ/þüÏÿÜËoûÛI&96¢î¹ë®»®Ç~ûkÖ¬0aÂo¾ùûßÿ~Ë-ååå]êîìãî¾ûîÿüÏÿï¿ÿþ¥KöØÙxægFýâ/:µuÖYÕÕÕÑÎÉÉéáràÀóÏ?óæÍ¨É'¿õÖ[=öÛ>zxÄ×¯_ß<Aöu%%%µµµÑá¦MÂ¹­ÇÎFjjêêÕ«ÿðá+Y¨wÞygàÀ=|n¸áGyÄ±õÚk¯M:µ'Ï@ø¡hllFéÀÙ×¥<xÿþýÑáÞ½òó:Ñç·Ú£Þ÷Ý²²²oûÛ=y|ðÁ/ùËÖ.]úoÿöo=y²³³[ÿÎkHÀÈ¾.-%%åèÑ£ÑááÃMSÄ%KN?ýôqãÆUVVöØIüJ_ôïØ(..Þ±cGOÏîs7nì±þÝ@RRÒ#GZg_AÓâÔÞÚûï¿_ZZú_ü¢~ïÑ_ésl´öæo?¾OBø9sæ-[:´fÍóÎ;/99Ù±²¯KËËËký"oSSÏ.qjo¯ªªêsÎéßxôWú­ýË¿üË%KÌÃ<0tèÐð_åéÓ§?÷ÜsÙÙÙæd_þ·ZSSVWWÇ/ÓâÔÞÆÁæÓÀ½£ög¶lÙ2?­­[·Î[:@öuu÷Þoë'3~üãß÷Ý¦ÅyèÐ¡6m7nÜ8qâDc#âÂ/~¹sç¶þü¿ÿû¿_°`cd_¶k×®qãÆ­Y³fß¾Ï=÷ÜðáÃ·mÛfZÚÿñÿñÊ+¯|ï½÷9R[[;úô§~ÚáØ<x°&3ùæýû÷?ùä#FøÍo~ãØÙ×Õýìg?+**JHHÍ÷ïÿþï&Ä©=øàî»ï¾3Ï<399yìØ±O<ñ£Â±¯/øÿøüüü¬¬¬Ë/¿üÕW_5! û²Ùì@ö ûÈ>d²@ö ûÈ>d²Ùì@öÈ> ËÚµkWFFF¯^±~`~úéÒÒÒ¼¼¼yóæmÚ´éO?êêÌ¿µvíÚ©S§sÙ|ÖgÎ»Ûyä^í¤§§WUUhöuþÈ>àSóë_ÿzôèÑûö]cÃÞvÛmÛ¶m;tèPeeåÅ_¶ðìÀ)øíÕ+4ß²eËb×XJJJ¸tãÆÑ-6m[²²²ÚÇÃ¥¥¥iiióçÏohh^¹~uuuyyyvvvFFFèÈÚÚÚ6¸jÕªÐ¦ÉÉÉS§N÷Ýwõ«_9255uÆÛ·o^9Æ~ÐÓM8qÝºuèèI¸9sæK®¾úê'x¢¥¥¥>¶É¾Pi­¯¢¢âÙWWWÓzcnnn4æ"[úôé½ôÜsÏ4hÄ¥^¹fìý ûct[õõõãÆUvvöÍ7ß¼uëÖÙWZZZSSÓØØxë­·F¾äÿÐ¢EÂpÚ´iÛ?TRR.låo¼qÏ=Ë/,X_7ÌÈÈèÌ~@§²/8räÈO~òË/¿<úd[jjêóÏ?¼ì¾ÆÚÐÐ½÷>æ?aUUU<¸õëêêþðá[O"ÃmÛ¶áÁ[ï*ö~@g³/jÿþý+V¬2eJä%×ãe_ôà£G¶¿4º·¤¤¤0IFÚ.11±õCq¶>|¸ý®bïÙt*û.ýõ×£[vïÞ¶¤¤¤/û·ó6´~.¬aaaá1¯c?È> SÙ·xñâpé	6nÜxèÐ¡ºººo¾9l=zôf_BBBäuÛÆÆÆ0¬¨¨ÃÖ¿þ¹Í¾Øû@öÊ¾;w4¨Íûp÷îý³ýìD³/òÑÏü«­­ü¨¼¼¼úúúÍ¾Øû@öÊ¾ ¦¦æ_üâi§SZZºzõêc~yìì[±bÅ°aÃRRRJJJ"[6oÞ^V¶lÙÒÎk?±d²ÙìÈ>d²Ùì@ö û²Ùì@öpêü=pÕIEND®B`


;'WÎ!ÎsNÂ'Lì@ö ûÈ>d²Ù ûÈ>d²ÙìÙ¿2§v×,WWWÏ3çÞ¤!mÛ¶-^¼8777;;æÌ¯½öû	 ûÙpÎ[mh³oóæÍý¦âÙguWdÙ÷jµ¡Í¾ÒÒÒðÕ-[¶ÿþ>úè£a8iÒ$w@öqmmm«V­*((HMMõóÈ#tuuõ»îI&¬¯¯¯¨¨ÈÎÎÎÌÌ,//oll<ÕøÅ/~qíµ×¦¤¤L<ù¿üe¿ã8=_yåk®¹&999rõ¾G>Õuc<ÁyóÍ7geeíÝ»7²ç¡CÂúppw@öq!úµÑÚµk?7ûsrrú®ÌÍÍmjjxüÍ792ºÛ¨Q£úÞ¤ØÇ¬IJJêõ;v|îuOÿ¼öo¼Ö*uWdÙ£233Ãò»ï¾ÛÝÝýÂ/å	&LÆ~Ù´jÕª0;wnÓ§JJJÂpåÊoÀâÅÃ¦EýîS·ÜrKßCÅ>NdÏÒÒÒ°iïÞ½ó³wÜqÇÞS	×8qb¸ÖúõëÝUÙÄyöÍ1#,®Y³fÃý®Òìi555ammm?~à<!=ýºsçÎ¾Èõõõa]]]PûÜëÎ³áhá»+ï¹ç÷@öq1vhhh:uj4Æ»qãÆÏÍ¾äää0<zôhd"gc?²gwwwß=£Èý¶Fÿ/Æu?7û¶oß>nÜ¸°fùòåÇw?dÿÙñÎ;ï<úè£,;nöåçç÷¦-,aAAÁÀ#_~ùåaÓ~F±*öq"~ðÁa8H^rÉ%gzúy÷ÝwÇvßò'ÜIÙÙyÎìÅ_ìîîñsrr^7ò^wÍÍÍíííaXUU%%%ÿ®nõêÕ×]wM.»ýîw¿ü^ô°±ÙsÖ¬Y»wïÞ·oß­·ÞÚ÷oûNÿ6ôõûßÿþÒK/~ïßs÷doÙãçí·ßÞoý5kf_qqqdX^^×Dåååµ´´¼555ß9ú²ÜÈÖØÇ¬itù?þã?Nçº§ò'ò'ójÙïìëìì|øáCÕ¥¦¦ûÛß>räÈÀìÛ°aÃ¤IÂ>%%%5uuuYYYaa×®]§º¿úÕ¯&Oyã½_üâý2+Æq"¾ùæS¦L	W>zXîäÓ¿Q]vìdÀ±YÍ ûdì²Ùì@ö·Ù÷äO677ûAÄyöýõ_ÿõGä	 û²OöÈ>Ù ûdì²OöÈ>Ù û²OöÀpÿuþ©íÛ·¦§§3¦²²²­­ÍÌ û ³/%%%¡ªª*3ì8Ì¾ÒÒÒöööz(³³³Í²â0û#Ã¶¶¶01bAö@f_wwwdØÛÛYcfÙ|áî[¶l6mZZZÚôéÓßï½°fÛ¶m'NLJJ:uêæÍeÈ>â!û®ºêª7=zô?øÁäÉÃòòò§~º³³óñÇ/++¯¼òÊvøÂûío;$Ù'ùf_TWWWä%Hùùùaaÿþý³oëÖ­¿/¼Ê>dßIüë¿þë+ÂBrrò'ÂBOOORR¼ñmmmßøÆ7"ÿ+JOOïííd_H@Ù'Ù·oß¾ÊÊÊ=öDÅÅÅimm-((ñ¯¿þú¬Y³öïß]ð©§êêêzòÉ',Y"ûâ!ûû~`XS]]]PP6mÙ²Eöpqýª>ídßsæÌÂì;¿d²ï¼öì²k "ûd¶¶¶U«V¤¦¦N4éGéêêsåíÛ·¦§§3¦²²2òÂÇÏì____QQY^^ÞØØØ¯7nÜ8eÊ9sæìÝ»÷7Þ¸ækÒÒÒæÏßÔÔ¤&eìs,ÄY¿n[»ví©²/TZß=«ªªNÍÍÍ999Wæææö¹#GF·^ýõ!:£ÃÅË>Ù'ûàËÌÌiõî»ïvww¿ðÂayÂ	§Ê¾ÒÒÒöööz(#j5°ÏV­ZsçÎmúTIII®²ïÎ÷ÝwßÁ#_1X¾|y>ÿüóa9Ü$Ù'ûdc3fÌiUXX¸fÍ6tvvþï¯êÙ=WÛÖÖ#F8ipÃÈ°¶¶6Çßwçæææ°|ôèÑÈ0ò¦¹]]]>ÛMöÉ>8/¦N=Á:vìØ7*ûº»»#ÃÞÞÞ!é"ÃHÛE?æ4²sOOOßáñãÇOõEýdìsæwÞyôÑG,X2«¨¨èt,ÆÖüüü¾Ïö0~ÞU¿ceìpÎDñÅ»»»Cüå3Í¾ÄÄÄÈyÛööö0¬ªªÃ¾Û·zõjÙ'ûdÛo¿½ßKq×¬Ys¦ÙW\acccä"Qyyy---²OöÉ>2?üpè¶ÔÔÔ¢¢¢oûÛG9ÓìÛ°aÃ¤IÂJJJ"kêêê***²²²222ÂÂ®]»N§ódì²OöÈ>Ù ûdì²OöÈ>dì²OöÈ>Ù ûd+·Ý~WÂçIÑØØh®0M¿ivæ/ýþþqÿ°ººÚûà"Ê¾²²²ôôô¬¬¬eËüñÇæPöÉ>ÃìûÞ÷¾·víÚÃwvv>üðÃùieì8Ì¾o¾yÇåÝ»wÏ?ßÊ>Ùq¹¹¹å#Gé¥æPöÉ>ÃìKJJêíí,04²Oö@f_bbbßìKNN6²Oö@f_nnî#G"ËÇ»äKÌ¡ìÙ7kÖ¬Ý»wG÷ïß?öls(ûdÄaö=öØcú§ÚÞÞ~ôèÑïÿû?þ¸9²â0ûZZZ,X^VVÖÑÑaeìa`É7?ÿ3yÓ|&/²@öÉ>Ù'ûdìæ?þñ­·ßûr÷½÷:tÈû`vã	S¦$Ìã2:77úJ^0l³oáÂåËc'MÈ>¸³ïðáÃfOöÉ>çìëèèX´hQBBÙ²â9ûn¼ñÆÙ'ûdÄyömÚ´éÿýÖ²Oö@|gßÿüÖ²OöìCöÉ>È>Ù²Ù7Ü³ïõ×__?Xî[È>dßðÈ¾ðØÈùÃe¸¤_õµ;¿µÂóä«3f½òÊK¦OqõöÛoË>dßéfßØïïÄ%ýï/7ï¿ÿþçwzé¥L²Oö²OöÉ>@öÉ>ÙÀ¼È>ÙÀEáæëo6æ²¹ã®qÉ9ð%È>Ù'ûNfþÁßÉ½.ïks'|dìÄsöÕÕÕÝrË-YYYãÆ«ªª:pà9²OöÙ7uêÔõë×:t¨½½íÚµ÷Üs9²OöÙ××Á³³³Í¡ì²8Ï¾ÖÖÖI&CÙ'ûdq?þñ¿ûÝïCÙ'ûdñû÷ï¿í¶Ûº»»Í¡ì²¸Í¾PwÝuWKK	²Oö·Ù·k×®oë[uuufOöÉ>Ù@Üfß¦M¦MÖÔÔdêdì³ìûzúÕ÷e5Æå²Ì/E³¯¨¨(á³Ì¡ì²aàÙÿóÌ=w.yà¾û:d®²²Oö²OöÉ>@öÉ>ÙÀ°lÉÏÖØØh®²alî7üzæÜÿþÆ1.sNþálÈ>Ù'ûÛìkii¹ã;Æ±téÒûöCÙ'ûdq3gÎü»¿û»úÎw¾3þ|s(ûdì ³¯¯´´4s(ûdì n³ïÄ¿ÿýïÿþïÿþ®»î2²OöÉ>â6ûÊÊÊ222ÒÓÓ·nÝjeìÄmö===>øàÔ©SÍ¡ì²xÎ¾àÀÉÉÉæPöÉ>Ù@g_kkkaa¡9²OöÙwÝu×ýô§?íîî>tèÐÿù?öØcæPöÉ>Ù@f_ccã¢E222òóó¿ûÝï?~ÜÊ>Ù'ûUTøL^dì>Ù×ÜÜªëûßÙÙ÷òË/÷Í»uëÖUUUjÙ0,³oÑ¢E;wîì+V¬xî¹çbdßøÃÏ³É¾s¼µcÇåì;É3mÑå¢¢¢Ù³ggddÌ1#¡gûâ3û"zkkko¸áÙÏÙ)¿ôôtÙÙwÕUWmß¾½§§gÇ.ñ[·n½öÚkÓÓÓKKKe@üdßé²OöÈ>ÙÈ>Ù'ûÙ'ûd ûdìdì²@öÉ>Ù'ûdì²²Oö²OöÉ>@öÉ>ÙÈ>Ù ûdì²OöÈ>Ù ûd ûdìdìì²²@öÉ>Ù'ûdì²²Oö²OöÉ>@öÉ>ÙÈ>Ù ûdì²OöÈ>Ù ûd ûdìdìì²²@öÉ>Ù'ûdì²@öÉ>@ö¦¦¦+W?>))©  `õêÕ---² ®²¯¡¡!777á³òòòvïÞ-ûâ'ûÊÊÊB!ÔÖÖ=z´¦¦fîÜ¹aMEEììËÈÈÔÔÔ]ÃÌÌLÙ?ÙÒìëe@üdß%K"'ykjjä@Üf_NNN¿t3¦®®NöÄOöÍÍÍ«V­0aBrrr~~þ+víÚua¾Ùpá²oÉ>ó¹Ñ²Oöìû(ûNõTßèÑ£¯¹æ¿ù¿9~ü¸ìÛìzäGdÀ°Ï¾=öÕUóæÍ«­­íêêª©©5kÖM7ÝôñÇ?ñÄ!.¿ürÙ0ì³oþüùý>m÷îÝaÍÒ¥KÛÛÛÃBrr²ìöÙ©¥¥%ºfß¾¿íëîîq_þòC!Ýzë­õõõÇûðÃÃrXsåWnÜ¸1,L8QöûìõÕWGÕïeaÍK/½Y^»v­ìöÙ¼ýöÛ·ÝvÛ¸qãóóó¿ùÍonÛ¶-¬=zteeegg§ììB²àe·kÀEÞ® ®²ÏÛ5Ë>à¢È>o×,û"û¼]³ì.ìóvÍ²¸(²ÏÛ5Ë>à¢È¾O¼]³ì.ìB²àÂeß[o½U^^Ó§Oùåe@ßÏþó#GFÿªïÿÿÎÏ>û¬ìì<yr(¤W^y%/¼ðBX¸â+d@üd_rrr(¤È[ôE²ïàÁa!55UöÄOöå+_	ôÓþ4­­­÷ßX6mììíµ×Nú¾¯¾úªìì>øà;ï¼óòË/OII0aBEEÅ;ï¼sa¾Ùp²/ú'QíííåååóæÍñÍÍÍaMZZìöÙ7gÎ®¼òÊÁ9Tc^^^t¸mÛ¶'&%%M:uóæÍ²àf_uuõ©/11±¸¸ø¥^Äa_~ùåpÝ¾O?ýôÓ?þxYYÙÀì]¸÷<8ì»íö;÷Àgµ¶¶*eöõV¬ßIÞ³±hÑ¢;wö=`~~~GGGXØ¿aaáÀìûùÏ¾í<8ì¿øÛà³Â/8åÁðÎ¾ðåËggg1¢ßÓ~gÑåäää'N¤¤$'y&û*++Ou¶÷d_zzzooo$ûBÊ>¡É¾1cÆBú³?û³¶¶¶Hä.#òéÓ²`h²oôèÑ¡8pn+ºùÔSOuuu=ùäK,C,´k×®óÕÕÕ[¶lCÛ¶mKII¹å[»»»/ð7 û.PöÅxÇfÙ ûdÀ°Ê¾¡%û.555­rüøñIII«W¯niiq¹¹¹ýNïæååíÞ½[öÄOöB*))©­­=zôhMMÍÜ¹sÃÙ?Ù©©©)º&,5² ~²/;;û¤ÙÖË>øÉ¾%KDNòÖÔÔ8É+û¸Í¾úúú~/é3fL]]ììW­Z5aÂäääüüü+VÛè_ìB²à¼gß¿ÿû¿ßtÓMÕÕÕWá¼yójkke@<d_zzzh£9sæô]aå%ÛßþVöûì«ªªaTXX¸eË¾ëßzë­ñãÇM>ø ìöÙ7aÂF¯¿þúÀM¯½öZØtÅWÈ>a!><pÓ¡CÂ¦´´4Ù0ì³/òá»wï¸iÏ=aSVVìöÙù(¿ø¿¸é¯þê¯Â¦o¾YöûìñÅC5*_ww÷±cÇvîÜùè£aÓºuëdÀ°Ï¾àÁL8ûï¿ÿÄ² ²/xóÍ7+++/¿üòÔÔÔ¤¤¤;î¸ã¤/ïÃ8ûì²@öÉ>@öÉ>ÙÈ>Ù'ûÙ'ûd ûdì²Oö1Ö¯_?÷w¹mÁCCdìc¸÷Þ¾âË¿9w¼1cÎÓ=dìãÜgß3ÓnüïoÜ9ËÄq²Ù'û ûd²dìCöìÈ>²Ùì²Ùì²OöÉ>dì²Oö ûd²Ï ûd²dìCöìÈ>²Ù²Oö!û²Oö!û²OöÉ>Ùì²OöÉ>dìCöìÈ>²Ù²Oö!û@öÉ>dÈ>Ùì@öÉ>Ùì@öÉ>Ù'ûd²OöÉ>Ù'û²Ù²Oö!û@öÉ>dÈ>ÙìÙ'û ûd²Ù'ûd²Ù'ûdìÈ>Ù'ûdì@öÉ>dÈ>ÙìÙ'û ûd²dìCöìÈ>dìÈ>dì²Oö ûdì²Ù'û ûd²dìCöìÈ>²Ù²Oö!û²Oö!û²OöÉ>Ùì;?ÂïÎ>d²â3ûÖ­[WUUuª­!û^õÕwÏ³É¾ùËÞeø»í¶Ûeg§&0|ÕÕÕ)d_,+V¬xî¹çbdßÖ­[Ï³É¾Ûn¿³áoéÒ¥Î¾Â±cÏÓ=¾>þøcåì¥¨¨höìÙ3fÌØ¹s§¼8Éñ½½½µµµ7ÜpìCö@<g_¤üÒÓÓe²â3û®ºêªíÛ·÷ôôìØ±cáÂ²Ùñ[·n½öÚkÓÓÓKKKe²â3ûbÈ>²Ù²Oö!û²Oö!û²OöÉ>Ùì²OöÉ>dìCöìÈ>²Ù²Oö!û@öÉ>dÈ>Ùì@öÉ>Ùì@öÉ>Ù'ûd²OöÉ>Ù'û²Ù²Oö!û@öÉ>dÈ>ÙìÙ'û ûd²Ù'ûdìÈ>Ù'ûdì@öÉ>Ù'ûd²Oö!û@öÉ>dÈ>ÙìÙ'û ûd²Ù'ûd²Ù'ûdß°öÑ`íÛ·o¸gßñãÇÏòÛ@öÉ>Ù7<üð?ÌHIË3nÑ)©555Ã:ûxâäÑ£3ÆÄ%)9YùÈ>Ù'ûÓcIÆ5ëòîÄåÚÜÕÕÕÃ:ûÂ·pÝu	ËâÊÏjÙ'ûdìÈ>Ù'ûdì@öÉ>dì@öÉ>dì@öÉ>dì@öÉ>dì@öÉ>Ù'ûd²OöÉ>Ù'ûdì²Oö¹È>Ù'ûdì@öÉ>Ù'ûd²Oö!ûd²Oö!ûd²Oö!ûd²Oö!ûd²OöÉ>Ù'û²OöÉ>Ùwï¿ÿþúÁzûí·=Ù'û²oxø¯-mâÄÉq9j  ûd²OöÓn¼1aáÂÁM`ø×Ã²Ù'ûdìÈ>Ù'ûdìCöÉ>Ù ûd²OöÉ>Ù'ûdì²@öÉ>Ù'ûdìdì²OöÉ>ÙÈ>Ù'ûdì²²OöÉ>Ù'ûdìCöÉ>Ù ûd²OöÉ>Ù'û²OöÈ>Ùì²@öÉ>Ù'ûdìdì²OöÉ>ÙÈ>Ù'ûdì²²OöOûöí»ùòo.[6ËÒ»ïnll²Oö¡Çÿöo÷ø<ó	Ù'û."ë×¯OÍÏO9spÅpuÙ'ûdßúR^^Âõ×æ!<eJ|²Oö]1yò «eòdÙ'ûdßÐg_EÅ`foáBÙ²OöÉ>Ù'ûdìdì²OöÉ>ÙÈ>Ù'ûdì²²OöÉ>Ù'ûdÈ>Ù'ûdì²Oöì²OöÉ>Ù'ûdÈ>Ù'ûdì²OöìÈ>Ù'ûdìÙ'û²OöÉ>²OöÉ>Ù'ûdìdì²OöÉ>ÙÈ>Ù'ûdì²dì²OöÉ>Ù'û@öÉ>Ù'ûdì²dß±mÛ¶'&%%M:uóæÍ²OöÉ>Ù'ûdÙW^^þôÓOwvv>þøãeee²OöÉ>Ù'ûdÙßÑÑöïß_XXØok(ÂY³fÍ;ÒÒ²rqyÙ¤+çqv¦M6"1qÔèÑ¸+«_õÕ©£²Fâ4rÔ×¾öµ¢¢¢1ÉÉ¦Ä%-1é<Ý3OSøöG&%nG%ñÿ"7~üÈÔÔÁM`bJpZFÆ¨´´AÌ^ö0ùñ:-kÖ¬QÈ¾XO8zzz.Ø×=pàÀGuüøq÷­³×ÜÜ<¸ùWaÐ?Áûö«ã ÐÚÚ:ä8¬oü;zôè '0üëaÏæÐ0ù&.ÒìKOOïííd_H@?3øÌ¾âââ¶¶¶°ÐÚÚZPPàgÙWYYùÔSOuuu=ùäK,ñ3Ïì«®®.((HLL,,,Ü²e@|f²Ùì±ç®¸¸8%%å+_ùÊ~ô#28ííí&L0¶nÝ:26U?käÈæä,°Ë ûâÖGtõÕWÿú×¿îèèØ´iÓ¾ô¥Í73uàÀ[o½UµÚý×Í1Ã¥ßüæ7÷Üsy8¬Ç2È¾xöØcýË¿üKtø£ýhíÚµ¦åüêW¿2eÊ-[üª#GÜxãuuu&ð,Í5ëÃ?4~Àz,ìs%%%ÑáÎ;Ã/`ÓrFÒÒÒ6mÚôÉ§§ÛÌÆ Üï½Ï?ÿ¼	<Kï¿ÿþ9sÌÃÙ<`=AöÅ¹ñãÇ>|8:ìèèÈÉÉ1-g¤¾¾þîß~U¹uëÖýñÿ±	<k×®ýÉO~bÎæë±²/Î¥¦¦öööFÇOJJ2-¼ûUq"Ò×ÕÕeÏ^QQÑ¾ûÌÃ9yÀº+ìOÉÉÉ===³/ i@ôOúLàÙûÏÿüÏiÓ¦Ù²XòòòúäíììôÎ²ïÂþI	<ÿðÿ°fÍó û@öË¢ÃúúúyóæÙwa¦ë¤ÌÌ üÑýÑ3Ï<cdÈ>byâ'ú>ãòÿø=öi&px¹é¦Þyçó û@öKkkëu×]÷æo:tè7¿ùMqqñ=Lj1ÃËøñãÍìÙÇçøÙÏ~VXXïþéLj1ÃNJJJôÑÈ>È>d²Ù ûÈ>d²Ùì@ö ûd²Ùì@ö ûpjmmÍÌÌLHõÈýå/YZZ·lÙ²;wþïcþS§óµª««çÌcÎdp¡=ztÁ±»íùçO ##£¶¶öL³ïô÷@öçÌ¿ýÛ¿M2eÔ¨Q±k¬¸¸8løá÷ìÙsìØ±o¾9¬)//?ãdìà±ïg]c©©©aëï½]³sçÎ°&++k`ÌE·oß^ZZ>fÌÊÊÊ¶¶¶è¦¨ÈþõõõÙÙÙ¡#û5âÆC¦¤¤Ì3gïÞ½o¼ñÆ5×6þü¦¦¦èÎ1ìÝW¿úÕÍ7òyOÂ­K.õÕW»»»æc¿ìÖ·ðªªªNÍÍÍ999WæææFc.²fäÈÑ­×_¤A#/^Ù3öqÀIºm ë®».ZTÙÙÙ<ðÀîÝ»cd_iiiCCCûC=¹ÊI¿ÐªU«ÂpîÜ¹M*))	Ã+WöÝù¾ûî;xðà/¼._¾<#ny:Ç@ö§AOOÏ?ÿó?ýë_>ÙöÖ[o*û¢çXÛÚÚÂpÄ'ýB&LÃÈ°¶¶6ÇßwçæææO>éId¸gÏ0ìêêê¨ØÇ@ö§QÞ°aÃìÙ³#§ÑÁ½½½·F!é"ÃHÛ%%%õÝ9gßáñãÇ*öqÀießÄÃÖ>ø ºæÀaMjjê©²ïTï·5??¿ï³ta!Nºsaìã ûÓÊ¾Õ«W­Ó§Oï½÷;ÖÜÜüÀ5S¦L9ÓìKLL·mooÃªªª0,))éû7yáËiöÅ>²8­ìÛ¿ÿ%ïu¸#FøÙÏ~v¦ÙyÀèþ566F> $*//¯¥¥åL³/öqÀie_ÐÐÐp÷Ýw_zé¥999¥¥¥6m:éÕcgß&MZRRYSWWWQQvíÚu:7pã8È>d²Ù ûÈ>d²Ùì@ö ûd²Ùìàû¿>ï¯ÿ!DIEND®B`


2 Sintomo * n. sintomi * Esito dopo


Test del chi-quadrato	
Esito dopo	Valore	df	Significatività asintotica (bilaterale)	
1	Chi-quadrato di Pearson	4,950b	8	,763	
	Rapporto di verosimiglianza	6,161	8	,629	
	Associazione lineare per lineare	2,116	1	,146	
	N di casi validi	11			
2	Chi-quadrato di Pearson	6,799c	4	,147	
	Rapporto di verosimiglianza	7,484	4	,112	
	Associazione lineare per lineare	4,064	1	,044	
	N di casi validi	11			
Totale	Chi-quadrato di Pearson	11,236a	10	,339	
	Rapporto di verosimiglianza	14,462	10	,153	
	Associazione lineare per lineare	6,503	1	,011	
	N di casi validi	22			

a. 16 celle (88,9%) hanno un conteggio previsto inferiore a 5. Il conteggio previsto minimo è ,05.	
b. 15 celle (100,0%) hanno un conteggio previsto inferiore a 5. Il conteggio previsto minimo è ,09.	
c. 10 celle (100,0%) hanno un conteggio previsto inferiore a 5. Il conteggio previsto minimo è ,27.	


Misure simmetriche	
Esito dopo	Valore	Errore standard asintoticoa	
1	Intervallo per intervallo	R di Pearson	,460	,116	
	Ordinale per ordinale	Correlazione di Spearman	,609	,147	
	N di casi validi	11		
2	Intervallo per intervallo	R di Pearson	,637	,196	
	Ordinale per ordinale	Correlazione di Spearman	,529	,260	
	N di casi validi	11		
Totale	Intervallo per intervallo	R di Pearson	,556	,092	
	Ordinale per ordinale	Correlazione di Spearman	,631	,124	
	N di casi validi	22		

Misure simmetriche	
Esito dopo	T approssimatob	Significatività approssimata	
1	Intervallo per intervallo	R di Pearson	1,554	,155c	
	Ordinale per ordinale	Correlazione di Spearman	2,301	,047c	
	N di casi validi			
2	Intervallo per intervallo	R di Pearson	2,482	,035c	
	Ordinale per ordinale	Correlazione di Spearman	1,872	,094c	
	N di casi validi			
Totale	Intervallo per intervallo	R di Pearson	2,995	,007c	
	Ordinale per ordinale	Correlazione di Spearman	3,640	,002c	
	N di casi validi			

a. Non viene assunta l'ipotesi nulla.	
b. Viene utilizzato l'errore standard asintotico presumendo l'ipotesi nulla.	
c. Basato sull'approssimazione normale.	

È>Ùì@ö ûÈ>dÐÇeNî?6~Ó¦M%%%½xÎùdöíÛu>Ü@öGÙ×­v>dß¡CfÏ( ûÎ£ÆRöýçþç	,ûÙô£ìknn^¶lYaaaZZZqqñC=ÔÖÖÖécOø4ammmEEE,ËÊÊ*//¯¯¯?Ù?ñÝï~wÒ¤I©©©ãÇî¹ç:Ý¥nßóg7nüÃ;ùdÛýáJh¾GTö²èGÙ²©S­òC³¯±±177·ãÆ¼¼¼®Çß¼yó A»uz­ûãÄ·$''wüðÿüçú±ÝgßWîÕ¯Ï2d@¯e_7ÿµW^y¥½½íÚµáúðáÃ»&c§BZ¶lYXÎ1£á¥¥¥a¹téÒ®w`îÜ¹á¦ë¯¿þÍ÷]wÝuÕýqâvïÞ®åzzN»dì6mZ¸^TT´bÅuëÖµ¶¶°:RHÃ°¬ªª/«««ÃrØ°a]ï@ü	¹ÄË¯Û·oïx¨îß³¶¶6¾¬©©	Ë#G~èÇâo±È>@öQË¾nv¨««8qb".¸àõë×hö¥¤¤å¡CâËp%þjl×ãÇ÷looï¸gâPÝ'¾g§[Ã|èÇÊ>@ö²ïÄ¶lÙ²jÕªø1âC³¯   ã3máJXv=ò¨Q£ÂMo¼ñF|Æ.q¨îßóµ×^/ÃAÂò¢.êéøÃ@²/þÙO>ÙÞÞâ/ÍÍíú±IIIázcccKKKX.Y²$,KKK;þòåË»ÿÖ[o7Í3'ìöæoÆ>/qØîßsúôé;wîÜ³gÏ7ÜÐñgûNý>È>@öý%ûºyÅóæoî´Å]«hôèÑñeyyyXÖ××Ç$!??¿©©©ë¨ªª'c§_ËßÚýqâ[âa¸þÓþôT>Vö²È¾ÖÖÖx T]ZZÚ#î¿ÿþ÷Þ¯k­[·®¸¸8ìSZZßRSSSQQ®ìØ±ãd÷áûßÿþøñãão¼÷Ýï~·Slusø7n0aBøð©S§ë|ê÷Aö²à|oVsdì²@ö ûÈ>"_ûÚ×Ú"_þòñ_ø´È>dì²OöÈ>Ù ûdì²OöÈ>d²¢w:ßÖ­[ËÊÊ222²³³+++MÙÌ¾ÔÔÔ,Y²ÄdÁì+++«««kii¹ï¾ûÂ2²"õõõñesssX8ÐdÁìkoo/;ßb2È>`öu¿Î»/êêêiÓ¦¥¥¥M8qãÆ²dÑÌ¾)S¦<ñÄÞ°aC~~~×ìæg¶BDýë¿þë¬¹îÅËmï4U8üò¿<'Ù'9O³/®½½=Ä_HÀ®Ù÷ãÿøM¨?ú£?J47cÑßöÖ%<Ð*?8 ûÐÚÚ=pàÀþçö"/ýJøOÿ»··.è8ßíÖ®];|øpÙìD<û<.û²hfßØ±c_|ñÅöööuëÖ]ýõ²Ù'ûfömÝºuòäÉsæÌillÈ>ÙýýTÊ¿±iÓ¦sx½|RÈ>Ù²ïlöì²dÐWÙ'û@öçFssó²eËÓÒÒzè¡¶¶¶®1¿¾uëÖ²²²ìììÊÊÊð±âû×ÖÖVTTÄb±¬¬¬òòòúúúN¸~ýú	&¤¦¦ìÞ½û?üá¸qãÒÓÓgÍÕÐÐ &eìÙô²gºmåÊ'Ë¾Pi÷dÉ	³¯±±177·ãÆ¼¼¼N17hÐ Ä­_~yÎÄrîÜ¹²OöÉ>@/ËÊÊß°¯¼òJûÚµkÃõÄ×vÍ¾²²²ºººûî»/,c±Ø	ûlÙ²ea9cÆ÷åÒ¥K;î|×]w8p þ//Ë'x"wIöÉ>Ù²èeÓ¦Mß°EEE+V¬X·n]kkëoOÕ]²/ñZmsssX8ðÂ1,«ªªâËêêê°6lXÇãoqèÐ¡ør×®]aÙÖÖæo»É>Ù²8#êêê&Nxõ.X¿~ýÉ²¯½½=¾<vìX7!éâËxÛ%''wÜùèÑ£G9Ù?ês$ûdÈ> ×lÙ²eÕªU³gÏß¼#F8ëæÖÏö+aYXXxÂ»YÊ>Ù'û@ö½&þÌÜO>ÙÞÞâ/ÍÍíiö%%%Å_·mii	Ë%KeiiiÇí[¾|¹ì²dpÎÜ|óÍ~wÅ=Í¾Ñ£GÇåååaY__ÿMüüü¦¦&Ù'ûdÈ>àimmàB·¥¥¥1âþûïï½÷zëÖ­+..G(--o©©©©¨¨ÈÉÉÉÌÌWvìØq*'ûdìÙì û²d²Oöì@öÉ>dì@öìÈ>²Ù²Ù'û@ö ûdÈ>dìÙì ûÞrÓÍ·ø0©C2ëëëÍÙ²èÃ¦^ý©¬îþÛèøOnÚ´É¬ û~555×]w]NNÎÐ¡C,Y²ÿ~3²dÁì8qâ5k<ØÒÒ²råÊE¡ì ûf_GÅbf(ûdÈ> âÙ·oß¾ââb3²dñìäG¾ô¥/¡ì û(gßÞ½oºé¦ööv3²dÙìµwë­·655 ì ûÈfß;n¿ýöÓ²dÙìûÁ~0eÊ£²dåì1bD§?Ýf²Oöìúùùð¿Éîoò"û@ö ûdÈ>dìÙì û³æáG¹áæ»¿Üvç4+dÈ> rÕU&LpÍ5Ýäåð³!ûdÈ> Oeß9/îæ+.È>@ÿÊ¾¦¦¦Á·ÜrË=ÌPöÉ>@³ïk®ù¿ùïðÁgÍe²Oöì"½óÎ;éééf(ûdÈ> ²Ùwüøñ·Þzë«_ýê­·Þj²Oöì"óæÍËÌÌÌÈÈøñl²Oöì"ÁÑ£Gï½÷Þ'¡ì û(g_°ÿþ3²dñìÛ·o_QQÊ>Ù²`öM<ùÛßþvûÁÿøÿøOÿôOÍPöÉ>@pÅ´isÑÔ©Ýää¼øâñýëëë¯¿þúÌÌÌ/éKG1CÙ'û@öÀ«¯¾ºæÃ<õÔSìÙì û²dp>ð"/²dì~áÚË¯É¡c»¹äÉJüJÈ>@tÍÿºòÁÜO­Î_ÐÍeRÞÈ¾o²Oöì¢/¼ðÙ'û@öÑÏ¾o¼ÑÃì ûgß+¯¼rûí·X²dñì+//ÿÙÏ~æaAöÉ>@³ïõ×_=öoNüdìÙD8û.½ïOöÉ>Ù²röÕÕÕM4éNüdìÙD5ûÊËË|Ê>Ù²`öàÄïaAöÉ>@Ê¾3ÆÞsE7K².È>@ßöØÿtÑgv¹ç®»<hVÈ>È>Ù²Ù'û@ö ûdÈ>dìÙì û²Ù'û ûd²dì@öì@öÉ>È>Ù²Ù'û@ö ûdÈ>dìÙìÈ>ÙìÙ'û ûLÙ²Ù'û@ö ûdÈ>dìÙì û²d²Oö!ûd²dì@öìÙ²Ù'û@ö ûdÈ>dìÙì û²Ù'û²Ù'û ûd²dÈ>È>Ù²Ù'û@ö ûdÈ>dìÙìÈ>ÙìÈ>ÙìÙ'û û²d²Oöì@öÉ>È¾ÓôÒK/M2%==êÔ©Û¶mÈ>Ù@4³ï²Ë.[¿~ý¡C~øáñãÇË>dì ÙÐÖÖÅº¿õ­omºóÎ;=ûLúÊW¾2fâå½xY¸diüÈS®¼òÒqãzëò±ÿå_þ¥oÍöµ×^SÈ¾ø÷ÿ÷;î¸£kö½úê«ÍQ>ø`¯g©ÒS·Ýv[ZÙ½Y÷>Ý+;¿1tØ%ñ#§eg3§·.i#F|ýë_ï[³çw²¯³ð½ñéOúÀ^äÅ¼^äå,»óÎ;3ýmoæ|y[^Áø3°xqo]2Ç_³fÏôíìÛ³gOeeå®]»NxRÈ>Ùììþùç§O¾wïÞe²Oö!ûdD!ût û²Ù'û Ù÷¡'EÙìÈ>Ù²dìCö²dÈ>²dÈ>²d²Oöì û²dìÙì ûdÈ>dìÙ'û²Ù'û@öù´!ûd²OöìÙ'ûìÙ²dìÙ²dìÙì ûdÈ>dìÙ'û@ö!ûdD'û.]:lØ°äääÂÂÂåË755É>È>ÙÊ¾ººº¼¼¼¿sçNÙ²Ù'û :Ù7oÞ¼pj)--­®®>tèPUUÕ3ÂÙ²Ù'û :ÙN--ázØ%û@ö!ûdD'ûb±Ø	³/l û²¢óçÏ¿È[UUåE^È>ÙÍ¾ÚÚÚÜÜÜN¿Ò]SS#û@ö!ûdD'ûÆÆÆeË><%%¥  à;îØ±cÇ:)Ê>dìCöÉ>8gÙw6O²Ù'û²ÎjöÅ_ÌM!Ù²Ù'û@öÉ>È>Ù!ûÎÕIQö!ûd²OöÁ¹É¾=Õ7dÈqãÆýùÿù#GdÈ>dìÈf_ÂC=$û@ö!ûdôùìÛµk×e]6sæÌêêê¶¶¶ªªªéÓ§_õÕo¿ýö_ýÕ_³Î¨Q£dÈ>dì>³fÍêôÇÙvîÜ¶ÜrË----áJJJìÙìÐç³/555Z[öìÙÿÙ¾öövÙ²Ù'û "Ù÷±,Zn¸áÚÚÚÃ¿ñÆázØ2fÌõë×+#G û²ú|ö=ûì³îôkaËSO=¿¾råJÙ²Ù'û Ïg_ðâ/ÞtÓMCMJJ*((øÌg>óòË/íC©¬¬lmm û²¢gó¤(û²Ù'ûàÜd·kÙìÐ¯³ÏÛ5ìCöÉ>Töy»fÈ>Ùý"û¼]3È>dì~Þ®d²OöA¿È>o×²Ù'û _d·kÙìÐ/²ï×Þ®d²OöA?É¾³yRÈ>ÙìpÎ²ï^(//ÏÏÏOJJË©S§>ýôÓ²d²OöA¤²ï;ßùÎ A?Õ÷ëÿÿÎ=öìÙìì?~|8µ<óÌ3ì[»vm¸ré¥Ê>È>ÙÑÉ¾pj¿E_üsàÀp%--MöìCöÉ>Nöüã§oûÛñìÛ·oßÝwß®L2EöìCöÉ>NömØ°áïÛ÷ì³ÏÊ>È>ÙÑÉ¾àµ×^ûìg?;jÔ¨ÔÔÔáÃWTTlÙ²åe²Oö!ûdìKüH_BKKKyyùÌ3eÈ>dì(g_cccØ.û@ö!ûdôùì+))Ð­1cÆÈ>È>Ù>û6mÚt²àKJJ=zôSO=%û@ö!ûdôùìûí!º¼ÈFO²Ù'û²ÎMöíÛ·oñâÅ±XlàÀö û²¢'µWöìCöÉ>NöeggSË¾ðæææcÇé¢ìCöÉ>dìsC	§ýû÷¢ìCöÉ>dìs³gÏ§;vÈ>È>ÙQÎ¾_~955õºë®«¯¯ooo û²¢Ý¼c³ìÙì ûdÈ>dì>gù¤(û²Ù'ûàe_CCÃÒ¥K¸|ùò¦¦&Ù²Ù'û RÙWWW×éåÝüüü;wÊ>È>ÙÑÉ¾yóæSKiiiuuõ¡CªªªfÌ¶TTTÈ>È>ÙÑÉ¾ÌÌÌpjihhHl	×Ã¬¬,Ù²Ù'û :ÙÅNa»ìÙìì?~üEÞªª*/òìCöÉ>löÕÖÖææævúìììÙ²Ù'û :Ù466.[¶løðá)))wÜqÇú½²Ù'û²ÎeöÍ¢ìCöÉ>dì³?ùÉO®¾úêM6uÜ3gÎ¬®® û²¢µµµá¤RRRÒqX]tÑ/ùKÙ²Ù'û Ïgß%KÂ¥¨¨è¥^ê¸ý^6lX¸éÞï û²ú|ö><Qþù®7mØ°!Üté¥Ê>È>Ù>ûÂåÝwßízÓÁÃMééé²d²OöAÏ¾øçØ¹sg×víÚnÊÉÉ û²ú|öÅÿÇ¿øÅ®7ýÉüI¸éÚk¯ û²ú|ö=ùäá2xðàP~µµµíííÞ¾ûªU«ÂÆpÓêÕ«eÈ>dì>Á½÷Þ;à$î¾ûîãÇË>È>ÙQÈ¾`ãÆ£FJKKKNN.,,`Á	½WöìCöÉ>èÃÙwöO²Ù'û²dÈ>ÙìdÈ> ûdÈ> ûdÈ>dìÙ'û@ö!ûdÈ>Ù²Ù'û@öÉ>È>Ù²Oö!ûd²OöìóiCöÉ>dìÙ²Oö!ûÙ²dÈ>Ù²dÈ>Ù²Ù'û@öÉ>È>Ù²OöìCöÉ>²d²OöìÈ>Ùì û@öÉ>dìÙ²Oö!ûÙ²dÈ>Ù²dÈ>Ù²Ù'û@öÉ>È>Ù²OöìCöÉ>²d²OöìÈ>Ùì û@öÉ>dÏÈ>²ÙÈ> û@öÉ>È>Ù²ï,illÌÏÏÈ>Ùìåìúé§G²sìCöÉ>dìdßõ×_¿ûön²ïñÇþøú×¿_7¬·.ùEÅk×®¾?éõ¼lüw¾ó~5ÃEõzö=ßÏ|þóïÅ/Âpÿ¿®êo3,++ëÝìc9=ëÅìK;ö¾ûî½ýÅrñr]É¬34Û­[·*d_»uòìQøÞðÈ#äéß<<õÒ%6æðþ^Òë3Ì¸pøút·¾øÅ/özö½×Ïü&çÿï^ü:ì3ìÝì»pxQüÈ½ülß¸qáa'vÚËïâÿäÍíËªÜ/>C³=|ø°ò@öõ ûÎÐ¼kÖ¬ÉýäÂ^<ÝÿÉM6õ«/¦^aÖÅEýí5/òo/Pax÷©W>û©ÕùzåÊ¯ /_ ûdì²Oö¡ìÙ'ûdì²ÏeÈ>Ù'ûdì²OöìëÙIQöÉ>Ù'Ydì ûdìEöÉ>Ù²OöÉ>Ù'YÌPöÉ>dì²OöI3²Ù'ûdì²ÏeÈ>Ù'ûdìf(û@öÉ>Ù'ûdì²dì²OöÉ>Ù'û@öÉ>Ù'û$ì²dì²O²È>Ù'û@öÉ>Ù'û$ì²dì²O²¡ìÈ>Ù'ûdì,f(ûd²OöÉ>Ù'ûdÊ>²OöÉ>Ù'ûÌPöì²OöÉ>Ù'ûdÈ>Ù'ûdì²Oöì²OöIÙ'ûdÈ>Ù'ûdd²Oöì²OöI3²dì²O²¡ìÈ>Ù'ûdì,f(ûd²OöÉ>Ù'ûdÊ>²OöÉ>Ù'ûÌPöì²OöÉ>Ù'ûdÈ>Ù'ûdì²Oöì²OöIÙ'ûdÈ>Ù'ûdd²Oöì²OöI3²dì²O²¡ìÈ>Ù'ûdì,f(ûô²OöÉ>Ù'ûdÊ>²OöÉ>Ù'ûÌPöì²OöÉ>Ù'ûdÈ>Ù'ûdì²Oöì²OöIÙ'ûdÈ>Ù'ûdd²Oöì²OöI3²dì²O²¡ìÈ>Ù'ûdì,f(û@öÉ>Ù'ûdì3CÙ²OöÉ>Ù'ûdÊ>²OöÉ>Ù'ûdìÙ'ûdì²OöÉ>²OöÉ>É"ûdìÙ'ûdì,²OöÉ>²OöÉ>Éb²Oöì²OöI3²Ù'ûdìÅeÈ>Ù'ûdìf(û@öÉ>Ù'ûdì3CÙ²OöÉ>Ù'ûdì ûdì²OöÉ>Ù²OöÉ>Ù'Ydì ûdìEöÉ>Ù²OöÉ>Ù'YÌPöÉ>²OöÉ>Éb²Oö!ûdì²O²¡ìÙ'ûdì²ÏeÈ>Ù'ûdìf(û@öÉ>Ù'ûdì²dì²OöÉ>Ù'û@öÉ>Ù'û$ì²dì²O²È>Ù'û@öÉ>Ù'û$Ê>Ù²OöÉ>Ù'YÌPöÉ>dì²OöÉ>3 ûdì²Oö¡ìÙ'ûdì²ÏeÈ>Ù'ûdì²Oöì²OöÉ>Ù'ûdÈ>Ù'ûdd²Oöì²OöIÙ'ûdÈ>Ù'ûdd1CÙ'û²OöÉ>Ù'YÌPöÉ>dì²OöÉ>3 ûdì²Oö¡ìÙ'ûdì²ÏeÈ>Ù'ûdì²Oöì²OöIÙ'ûdÈ>Ù'ûdd²Oöì²OöIÙ'ûdÈ>Ù'ûdd1CÙ'û²OöÉ>Ù'YÌPöÉ>dì²OöÉ>3 ûdì²Oö¡ìÙ'ûdì²OöÉ>²OöÉ>Ù'ûdìÙ'ûdì,²OöÉ>²OöÉ>É"ûdìÙ'ûdì,²OöÉ>²OöÉ>Éb²Oö!ûdì²O²¡ìÈ>Ù'ûdìf(û@öÉ>Ù'ûdì3CÙ²OöÉ>Ù'ûdì ûdì²OöÉ>Ù²OöÉ>Ù'Ydì ûdìEöÉ>Ù²OöÉ>Ù'YÌPöÉ>²OöÉ>Éb²Oö!ûdì²O²¡ìÈ>Ù'ûdìf(û@öÉ>Ù'ûdì3CÙ²OöÉ>Ù'ûdì ûdì²OöÉ>Ù²OöÉ>Ù'Ydì ûdìEöÉ>Ù²OöÉ>Ù'YÌPöÉ>²OöÉ>Éb²Oö!ûdì²O²¡ìÈ>Ù'ûdìf(û@öÉ>Ù'ûdì3CÙ²OöÉ>Ù'ûdì ûdì²OöÉ>Ù²OöÉ>Ù'Ydì ûdìEöÉ>Ù7û^~ùå#G&''O8qóæÍ²OöÉ>É"ûdìhf_yyùßÿýß·¶¶~å+_7oì²O²È>Ù'û ÙWPPðÎ;ï+÷î-**êtk(ÂéÓ§Ï<¦L2(959'¯·.áhøÄ'fö'½>ÃÁ)igèÓÞ;vì Ô^arzÆÌ~fÄÓ³ÌðüaRÖÐÌÜãG<xÈÞºLJ;á°yùI©±ä!½rÉNJsf»bÅåìû­ãÇ+GMNN>ÿtccã/zÏ=úá×SïÎpß¾ýp¿èUû÷ïïo<räo3<xð`üÈáºpâ=tèÐºÃ ûÎ¬cÇÅ³/$ Ï@4³oôèÑÍÍÍñÿ),,,ôföUVVþÝßý][[Û×¾öµùóçûD3û6mÚTXXTTTôÒK/ùD3ûÈ>dìãüñÇ'NÅ~÷w·­­Í@>Õ«W÷Ã?¥Õ;4hÐ 3é©çnüøñC0aÂä4¬]»¶¸¸8ÌpæÌ?ÿùÏd_¤üã?þã¬Y³víÚµÿþ?ø?øýßÿ39mÿõ_ÿ5mÚ4Ù÷ÑýèG?Z´h9ôHMMÍïýÞï555=zt÷îÝûÜç~úÓKlÜ¸qêÔ©¯¿þú¯~õ«;vTTT$þ û¢à²Ë.«­­_åk&§ç½÷Þ»êª«Â©WötÓ§Oã7Ì¡GæÎûßÿýßåÞ½çÌc,=2sæÌïÿûå-[V®, û¢éÍ7ß¼ðÂÍáôÜyçO<ñÄ¯ß±Ò4>W_µ¤¤Äzª  àí·ßN,Cú;C=[ZZËÖÖÖqãÆÈ¾Ú½wyyù_þå_ÅiX½zõç>÷¹ÿùÍÊ+ÿéþÉzjÃø¸wïÞ£G¾õÖ[_øÂþã?þÃXz$uüùæYYYÆ²/jV¬XqÉ%L<¹ªªÊ4z*þ#³ìûF±gÏs8>úè Aâ¿óøãHOòÜ¶m[bùôÓOûÕ"õöÛoïß3Sø¾ß~MË¾àõ×_2e9Õ«WÏ?¿¡¡áÈ#;wî¼á¾õ­oKG¿Ù³gïØ±ãðáÃ7n¼òÊ+SSSd_dUWWüã7Sø>Ù×+þáþaÅæp.½ôÒ¿ãùô<öØc£FJKK9sæ~ô£X,f& û"«­­-<ÞC¾OÂdNÏïüÎï<úè£æpräÈÄòðáÃa±|7oö+ û"%üíöíÛËmÛ¶]qÅÆòCÐNÛÕW_½eËs8S¦LimmM,ëêê&Ol,=2þüÏþÅ_üÅâÅd_tüõ_ÿõÍ7ßüÖ[o=z´¾¾~æÌÏ=÷±È¾seØ°aÞ ÷ô|óß¬¬¬¬®®þÕ¯~f¸`Á?Û×Sanñ·h~÷Ýw7lØ0vìØýìgÆ²/:?þÕ¯~uÌ1©©©&MzöÙgÍDöCáëÐ_<m?üðèÑ£Â¿ñoÈi­<|øðo¼ñ'?ùì@ö ûd²Ùì@ö ûÈ>dì@ö ûÈ>d²Ùì>äå_;wn^^^,»æk6lØp²=î¹²²²ÜÜÜ¤¤¤üüünß¾ý·ßêï;qÓ¦M%%%& û³góæÍ¡á|Ðc=ÖuÏ'xb@ÕÕÕ=Í¾SßÙô²²²P`.Ü»wïV­ZÅÅÅ]÷=zt¸éØµk×áÃ«ªª®½öÚ°¥¼¼¼Ç²@ögYH·Ý»wÇAÒuÏ´´´pÓ¶mÛ[¶oß¶ïsñë[·nY]YYÙÜÜ¸)!¾mmmEEE,ËÊÊY__ß©×¯_?aÂÔÔÔpoøÃ7.==Ö¬Y»9²ø­S¡±&MÔõ¦yóæn¹åg¶½½½ó·zìÖ±ð,YrÂìkllÌÍÍí¸1///sñ-JÜzùåÇ4nîÜ¹ñ=»?²ø¡FjiÍ5]omjj<yr¢¨b±Ø=÷Ü³sçÎn²¯¬¬¬®®®¥¥å¾ûîH×=eËå3ÞWZZK.í¸ó]wÝuàÀµk×Æ/ËøfeeÊqÀoÔÔÔNZ´hÑÉö9zôè7¿ùÍo¼1ñd[zzú/¼p²ìK¼ÆÚÜÜ<aö><,«ªªâËêêê°6lXÇÃõCÅ»víË¶¶¶êþ8È>à×[·n:thüY´#G|èþï¾ûîºuë>õ©OÅ_r=Yö%^>vìX×[GKII	Ëtñe¼í;î³ã2q';ªûã û ¿åW²³³C!­Zµêøñã'Û-þúïk¯½Ø²ÿþ°%--ídÙ÷ßZPPÐñYºp%,O¸s7Ëîì~í­·ÞºøâCýÙýY÷._¾<ì6uêÔmÛ¶>|¸±±ñî	[&LÐÓì¿S`8BKKKX.Y²$,KKK;þL^øçzÝÙýÚç?ÿù'ÒuÏ½÷^tÑEv8pà¿ýÛ¿õ4ûâoxÏ¿úúú¬¬¬ÍÏÏojjêiöudôkÉ)f_PWWwÛm·]|ñÅIII¹¹¹eee?øÁNX`ÝgßºuëÓÒÒJKKã[jjj***rrr233Ã;vJçu]vsd²ÙìÈ>d²Ùì@ö û²Ùì@öpîü?Ð»?rIEND®B`


È>d²bgÞà£Ë6m>úi|H8'[·n;wîèÑ£#Èµ×^»aÃÏ@ö²/á´·ÚàfßæÍ»MÅ£>ê©È> ²ï¬jµÁÍ¾²²²ð¯ßqÇ÷î=xðàªU«Â°¸¸ØS@g_ssó²eËSSSCýÜÿýêvßã¾LXSSS^^D233,XPWW×Û?ñÜsÏ]yå)))ãÇþùç»=¤>Ýóg7nôî]ÜÛûxóºë®ËÎÎÞ½wtÏ¶¶¶°>ÜS@g_È¦nm´råÊÏÌ¾ÆÆÆ®+GÝÐÐÐóø7o>|xl·óÎ;¯ëCêû8Ñ5III]ïþî»ï~æOüºöË/¿Ö*õTdÙ×Geffå7ß|³££cíÚµa9??¿g2vË¦eËá3>QZZK.íùæÎ6Í3ç·¸ñÆ»ªïãD÷,++vïÞ½>Ë-·ô÷1ô&Üëâ/÷Z³f§ û8Ï¾©S§å¢¢¢+V¬[·®µµµÛ!Ã°²²2:¬ªªÃ¼¼¼ úòë;ºªïãD÷¬©©«««Ã0ÚgÞ÷D^íGÿ¯ÃÊEy²ìëcÚÚÚ'ÆÂhäÈë×¯ÿÌìKNNÃöööè0,D¯Æö<~tÏ®ÆÕ÷q¢vÛ^÷ýÌìÛ¶mÛ¨Q£ÂÅwvvz²ÿìÚ²eËªU«fÍv3fÌgf_AAA×WÚÂBö<òØ±cÃ¦÷Þ/:¾b;TßÇîùÎ;ïDá axÁô÷1tóæofeeÃÿåcÇy²8'²/úÙO<ÑÑÑâ/,çääô¼oô³î[ZZÂpÉ%aXZZÚõuË/ïyüÛo¿=l=vØí·¿ýmôýy±ÃöèÓ¦MÛ¹sç=æÍ×õ½'þºÚ·oß^öüîw¿ëéÈ> Þ²¯+7ß|s·õ+V¬è%%%ÑáÂ°®®.ú» 1¹¹¹MMM=@eee×GýZntkßÇ®ilù¿üåÜ·7ßøÆ7NîÃ«d0´³¯µµõ¾ûîU:fÌo~óôQÏì[·n]qqqØ§´´4º¦ººº¼¼<;;;###,Ô××÷ö^xáñãÇG?xï¹çëY'ºçÆ'Lî>eÊ°ÜõÈ'þb.ºè"ÙÈ>³±YÍ ûdì²Ùì@ö'Ù÷£ý¨±±Ñ Î³ï;ßùÎûï¿ïË û²OöÈ>Ù ûdì²OöÈ>Ù ûÈ>¿çØ¶m[YYYzzzVVVEEEss³Aö@f_JJJBK,13È>Ãì+++«­­mii¹÷ÞÃ0dÄaöÕÕÕEÍÍÍa8lØ03ì8Ì¾èðèÑ£Ñ5fÙq¯³î	ª®ëÛQeÈ>â3ûV¯^ÝÇoìæg¶Ý~øá7Þ8ð·ùåå¯½öù?kýæ7¿ì¥Ùwçw>öØcdßo¼ñ[³Ûm·ÝpÉ%	×^;À·ôt'É³ÙÁe²ïScÆ¹þúë322¦NºcÇy¡è®»îú¸Ã/à[Æ¨QNÀÉ¾¨£GVUU]uÕU²²çì_zzºìdìâ3û.»ì²mÛ¶9räÝwß=¶ìdìâ3ûÞxã+¯¼2==½¬¬¬±±Qö²Oöë?ªOø26mÚ4úôA|È¾ÓIö²OöÉ¾î3Ù'ûdìc¨"²OöÈ>Gssó²eËSSSï¿ÿþCõ¹èò¶mÛÊÊÊÒÓÓ³²²***Âcºý«òòòH$¹`Áºººn¸~ýú	&¤¤¤L>÷îÝ/¿üò¸qãÒÒÒn¸á5)ûdìã4qÖ­ÛV®[öJëºgô¯[õÌ¾ÆÆÆ®+GÝ-æÛúùÏ>Dgl8wî'ûdìã4ËÌÌiõæovtt¬]»6,ççç÷eeeµµµ---÷ÞoF"ãöÙ²eËÂpÆ(--Ã¥KvÝù+_ùÊÁ£ÿb°xñâ0|üñÇÃrxH²OöÉ>ÙÇi6uêÔVEEE+V¬X·n]kkë§?ªd_ìZmsss6ì¸Â1+++£Ãªªª0ÌËËëºsôC3ÚÛÛ£Ã]»vá¡Cüm7Ù'ûdgDmmíÄcXG¹~ýúÞ²¯££#:<zôh!é¢ÃhÛ%%%uÝùÈ#]½ý£¾F²OöÈ>N-[¶¬ZµjÖ¬Y!³Æs"ÖÇÖ®¯ö0,,,<îÎeì²Ó&úÊÜO<ÑÑÑâ/,çääô7û£×m[ZZÂpÉ%aXZZÚõ½Ë/²OöÈ>ÍÍ7ßÜíWqW¬XÑßì+)),XuuuÑßÉÍÍmjj²OöÈ>Mkkë÷Ýº-55uÌ1ßüæ7?úè£þfßºuëÃJKK£kª««ËËË³³³322ÂBýtì²@ö²OöÈ>@öÉ>@öÉ>@öÉ>@öÉ>@öÉ>@öÉ>@öÈ>ÙÈ>Ù ûd ûdìdìì²²²Óå¦oOø,)#2êêêÌ²@ö1M¹æúÌùû¸ÿM6+dìãÊ¾êêêo¼1;;Ô¨QK,9pà9²@öÙ7qâÄ5kÖ´µµµ´´¬rÑ¢EæPöÉ>ÙGf_WD"æPöÉ>ÙGgßþýûÍ¡ì²8Ï¾~ø0²OöÈ>â9ûöîÝÓM7uttCÙ'ûdq¡ön¿ýö¦¦&(ûdì#n³¯¾¾þÏÿüÏ«««Íì²¸Í¾_|qòäÉ¦NöÉ>ÙG<gß1cºýé6s(ûdìcXø¥Ïþ¼iþ&/²@ö²OöÈ>@öÉ>ÙÈ>Ù û0=üð¼oîûöå»îjkk3WÈ>ÙÇ6ùê«&Løø9ÖûmÄèÑÇýãlÈ>Ù ûRÙ7vßOHq±ìCöÈ>Î­ìkjjºå[Fqë­·îÙ³ÇÊ>Ù ûÃì»öÚkÿþïÿþà'þê¯þên0²OöÈ>â0ûºúàÒÒÒÌ¡ì²¸Í¾cÇíÛ·ïþán¿ývs(ûdì#n³oþüùéééo¼ñ9²@ö·Ù9räî8q¢9²@öÏÙ8p 99ÙÊ>Ù ûóìÛ¿QQ9²@öÙ7iÒ¤ýìgmmmý×ýàCÙ'ûdCÀL:òÒK/2¥Ûìì×^-º]]Ý9s222xàÎÎNs(ûdìcxûí·×|§zÊD!ûd ûdìdìì²²²²²²²²Oö²@öÈ>ÙÈ>Ù ûÙ'ûd ûdìdìdìd_Oë×¯OHHì@<g_ûøñãe ûdçÙ÷íoûïþîïzË¾uëÖý7ÀÙmÞ¼yi#G:IÍ*++²ïSõõõ'N<|øpoÙ÷Ö[oí8»ÝqÇé99Ng³(dß§nºé¦^xáãGæ"/à"¯¼@g_Âì@|f_×þë¹Rö²Oö²@öÉ>`fßqÉ>@öÉ>@öÈ>ÙÈ>ÙÈ>Ù ûÙ'ûd ûdìdìdìdìdìdìdìd3 ûd ûd$ ûd ûdìdìì²²²²²²²¾lìì²@ö²OöÈ>@öÉ>ÙÈ>Ù ûÙ'ûÙ'ûÙ'ûÙ'ûÙ'ûÙ'ûÙ ûd ûdìì²²@ö²OöÈ>@öÉ>@öÉ>@öÉ>@öÉ>@öÉ>@öÉ>@öÈ>ÙÈ>Ù ûd ûdìdìì²²²²²²²²Oö²@öÈ>ÙÈ>Ù ûÙ'ûd ûdìdìdìdìdìdìdìdìì²@ö²OöÈ>@öÉ>ÙÈ>Ù ûÙ'ûÙ'ûÙ'ûÙ'ûÙ'ûÙ'ûÙ ûd ûdììë¥Kæåå%%%._¾¼©©Iö²Oöqµµµ£GNøc¹¹¹;wî<£UUUM:555uâÄ7nìÀÙóçÏWZZ­½½½²²rÆaMyyùImòäÉ?þøáÃ7lØÚQö²OögKöeddÈkhh­	ËaMffæI³££#Ä_HÀÙ÷äOn8»Í3gP²/-q<½ýöÛÊ¡Hä¸ÙÖÜ[[[³²²öØcõÌ¾wÞy§àìVQQ1(Ù>jäÙ¬­­My0´³oáÂÑ¼§~7fíÚµùùù.ò.òºÈ-ÙWSSÓíW:²²²ª««Oå°á?ÒÒÒd ûdp¶d_ÐØØ¸lÙ²üüüäää;ï¼³¾¾þäuùå¿öÚkëÖ­3gìdìÎ¢ì;¶mÛ6iÒ¤ôôôÙ³gìÀ g_ôbnlá¸NûÃìì²8Ù7(d ûd0hÙ×ÛK#F7nÜ÷¿ÿýÎÎNÙÈ>ÙÄmöÅÜÿý²²òÙ·k×®Ë.»læÌUUUª¬¬6mÚ5×»ßýî?üaÈ¾±cÇÊ>@öÉ>`Ègß7ÜÐí³íÜ¹3¬¹õÖ[[ZZÂBrr²ìdì|ö¥¤¤¶kjj­Ù³gOô½²²ìûÜç>ÚnÞ¼y555~ï½÷ÂrXsé¥®_¿>,ñÅ²²òÙ÷ì³ÏwÞyÝ~#¬yê©§¢Ë+W®ìÀÏ¾àµ×^»é¦FXPPð¥/iëÖ­aý#***Z[[e ûdÙ7`d ûd0hÙçãdpNgkdìâ*û|ìÎìóqÍ²8'²ÏÇ5È>àÈ>× ûs"û|ìÎìûkÀ9Fö²Oö¯¾úêrssÃpÊ)O?ý´ìdìâ*ûþã?þcøðá±wõýáÿó£>*ûÙ'ûøÉ¾ñãÇÈægbÙ·víÚ°pÉ%È>@öÉ> ~²/999D^ô#ú¢ÙwðàÁ°*ûÙ'ûøÉ¾+®¸"DÞÏ~ö³höíß¿ÿ«_ýjX<y²ìdìâ'û6lØpÜÏíöÙge ûd?Ù¼óÎ;·ÝvÛØ±cSRRòóóËËË·lÙr&®ìdì-ûboéiiiY°`ÁÌ3e ûdÏÙ×ØØÖ¤¥¥É>@öÉ>`ÈgßôéÓúté¥Ê>@öÉ>`Ègß¦Mz¾ÄÄÄ§zJö²OöC>û>=D¼gìdì-ûöïß¿xñâH$2lØ°n/ûÉ>@öÉ> ~²¯¢¢¢·«½²²ìËÊÊ÷­o«¹¹ùèÑ£gôáÊ>@öÉ>`Ð²oÄ!û80Wö²Oö³fÍÙW__/ûdÏÙ·uëÖo¼±®®®££CöÈ> >³¯OlìììÀÊ¾$ûÙ'ûÁÌ¾¥Kæåå%%%._¾¼©©Iö²Oöqµµµ£Gîvy777wçÎ²²ì?~è¼ÒÒÒªªªöööÊÊÊ3f5ååå²²ìËÈÈ×ÐÐ[ÃÌÌLÙÈ>ÙÄOöE"ãf_X/ûÙ'ûøÉ¾F/òVVVºÈ û¸Í¾n¿ÒU]]-ûÙ'ûøÉ¾ ±±qÙ²eùùùÉÉÉwÞyçú½²²Ìì0²²ìë­·®¹æM6u]3gÎ¬ªªì@<d_MMMzzzBBÂôéÓ»®Ã°ò.øÍo~#ûÙ'û!K,	yWTTôúë¯w]ÿê«¯æååM÷Üsìdì|öåçç¶ûÅ/~ÑsÓÂ¦K.¹Dö²OöC>ûCÛøá=7µµµMiii²²òÙýã;wîì¹i×®]aSvv¶ìdì|öEÿÇßüÍßôÜô·û·aÓu×]'ûÙ'û!O<ñDh»óÎ;/_MMMGGÇáÃwìØ±jÕª°2lZ½zµìdì|ö÷ÜsOB/¾úÕ¯;vLö²OöñÁÆ+**ÆTXXxË-·÷×e ûd0³oÉ>@öÉ>@öÈ>ÙÈ>ÙÈ>Ù ûÙ'ûd ûdìdßÉyýõ×'O6eÊíÛ·Ë>@öÉ> >³ï²Ë.[¿~ûC=4~üxÙÈ>ÙÄgöÅ:t(ôÌ¾ÇìEàLúÞ÷¾WrÅûÜÄ?ýéOãcgÏ=(Ù8IÍz^ÅÙ÷±ÿú¯ÿºóÎ;fß¯ýë3iÕªU	%%	!ö÷Â/ÄÇ.Z´h°^ís<µ··+d_wÍÍÍögvðàAyaào´I>Y"ÅÅ6mr×E^àÊ¾=öTTTìÚµë¸?Ñ@öÉ>ÙÄCöýâ¿6mÚÞ½ûiä²OöÉ> ²¯¨¨(¡Ù²OöÉ> >³ï39£ì²ìNìdìdìdìdìÙ'ûd ûdÈ>Ù'ûÙ'û@öÉ>ÙÈ>Ù²OöÉ>@öÉ>²Oö²Ïdììdììììì ûdìdìÙ'ûd ûdÈ>Ù'ûÙ'û@öÉ>ÙÈ>Ù²OöÉ>@ö²OöÉ>@ö²OöÈ>@öÉ>@öÉ>@öÉ>@öÉ>²Oö²Oöì²²dìì ûdìdìÙ'ûd ûÙ'ûd ûÙ'ûd ûd ûd ûd ûdÈ>Ù'ûÙ'û@öÉ>ÙÈ>Ù²OöÉ>@öÉ>²Oö²Oöì²ì²ì²²²dìì ûdìdìÙ'ûd ûdÈ>Ù'ûÙ'û@öÉ>ÙÈ>Ù²OöÉ>@ö²OöÉ>@ö²OöÈ>@öÉ>@öÉ>²Oö²Oöì²²dìì ûdìdìÙ'ûd ûdÈ>Ù'ûÙÈ>Ù'ûÙÈ>Ù ûÙ'ûÙ'û@öÉ>ÙÈ>Ù²OöÉ>@öÉ>²Oö²Oöì²²dìì ûdìd ûdìd ûdìdìdìÙ'ûd ûÎ ÆÆÆÜÜ²OöÉ> ³ïé§.))IHH ûdìâ9ûæÌ³cÇ>²ïGyîÌX¹reöè¼¿]XT¼fÍçâÂÝwß=(s8æ²qO=õÔs&é]ôýïÿÔÿõeóåy8nÊÕ±Ç0kÖ¬AÉ¾´HäÌ$ØÍ·Ý5jÔÀßJJÎÜùä7ÞPÈ¾«÷ì«­­í83,Y¶ðÛÙßÙ>À·ÈeòÒK/uÄÁÃóóÏÜãôÀÎ«cÇïóó2¿þìÀ?Ã«ë÷Â`½Ú7ß®º*aÚ´òò¾9òÌÍagg§ò@öõ#ûÎÜõ»îº+Ñ?üÇ½|5þñtakPæ0óÂ"¶]0&DØÀ?»¸=u¯¾:aöls²OöÉ>Ù'ûdì3 ûdìCöÉ>ÙgAöÉ>Ùì²Oö!ûÆO#Ù'ûdì²Ïì²Ù'ûd9Ù'ûd²OöÉ>Ùì²Oö!ûdìÈ>Ù'ûdì²Oöì²OöÉ>Ù'ûdÈ>Ù'ûdìEöì²OöÉ>Ù'YdÈ>Ù'ûdì²Oöì²OöÉ>Ù'ûÌ!È>Ù'û²OöC²Oö!ûdìÈ>Ù'ûd²OöÉ>Ùì²OöÉ>Ù'ûdÈ>Ù'ûdì²Oöì²OöÉ>Ù'YdÈ>Ù'ûdìEöì²OöÉ>Ù'ûÌ!È>Ù'ûdì²Ïì²Ù'ûd9Ù'ûd²OöÉ>Ùì²Oö!ûdìs>AöÉ>Ù'ûdì²dì²OöÉ>Ù'û@öÉ>Ù'ûdì,²dì²OöÉ>É"û@öÉ>Ù'ûdìædì²OöÉ>ÙgAöÉ>Ùì²Oöì²Ù'ûdìCöÉ>Ù'û²Oö9 ûdì²OöÉ>Ù²OöÉ>Ù'ûdì ûdì²OöIÙ²OöÉ>Ù'ûdd ûdì²OöÉ>s²OöÉ>Ù'ûdì3 ûdìCöÉ>Ù'û@öÉ>Ùì²Oö!ûdì²OöÉ>Ù²OöÉ>Ù'ûdì ûdì²OöÉ>Ù²OöÉ>Ù'ûdd ûdì²OöIÙ²OöÉ>Ù'ûdì3 ûdì²OöÉ>s²OöÉ>dì²dìÈ>Ù'ûd²OöÉ>Ù'ûdì ûdì²OöÉ>Ù²OöÉ>Ù'ûdì ûdì²OöIÙ²OöÉ>Ù'ûdd ûdì²OöÉ>s²OöÉ>Ù'ûdì3 ûdìCöÉ>Ù'û@öÉ>Ùì²Oö!ûdì²OöÉ>Ù²OöÉ>Ù'ûdì ûdì²OöÉ>Ù²OöÉ>Ù'ûdd ûdì²OöIÙ²OöÉ>Ù'ûdì3 ûdìs²OöC²Oö!ûdì ûdìCöÉ>Ù'û²OöÉ>Ù'ûdìÙw¦lÝºõâ/NJJ8qâæÍeì²OöÉ>sñ,øçþçÖÖÖïïóçÏ²OöÉ>Ù'ûÌ!Ägö|ðÁaaïÞ½EEEÝ¶"6mÚÌ3cÌ1ç¥e&eàÛð¤?ýÓ?kÏKN=sOsÐå_><)é¼#ø6<1ñ´|/dä9jàIié]¿§¤ü7ß£òò§¦ÆÙ®X±By û>|ìØ±°päÈ¤¤¤ü§;;;ßöìçÓ`Íáþýû3^Cú¡­­mPÿ|/Fíííæâ<ûÒÓÓ=Í¾¾Bñ%%%ÍÍÍa!ü÷Vaa¡¯@|f_EEÅ?ýÓ?:tèG?úÑÂâ3û6mÚTXXXTTôúë¯ûÄgö ûÈ>ÙÇÙbõêÕ]ÿ¸ýxÿ±áÃþzþùçÇ?bÄ	&lØ°Áµk×99sæ»ï¾kBú¥¥¥%??¿ÛÊÇ¬¤¤$%%å+®øñl@öÅ_ÿú×S§N§îW^Y´hyèêêê¿üË¿ljj:räÈîÝ»ï¾ûî_þò¦¥_6nÜ8eÊ_ýêW¿ÿýïëëëËËËMË	:pàÀ¼yóºßÿýË/¿ü¥^úà^|ñÅóÏ?óæÍædß÷ÑG]õÕáG¯ì;uÓ¦Mï½÷ÌC¿Ì;÷ÿ÷cÃ½÷Î=Û´ôËÌ3_xáØpË-+W®4-'"ÌÛ	^ýõn'À|ðßÿýßcÃÿøÇ¦d_<¸ë®»üñ?|r±Òl·ß~úôéæ¡¿~÷»ßÅ!ýþ:ÿüó[ZZbÃÖÖÖqãÆöâ/ö<ÖÕÕÅ;vìÿylº@öm«W¯¾ûî»ÿïë!ûNÍÊ+ÿõ_ÿÕ<ô×¾þõ¯ïÝ»÷È#ûöíûÖ·¾õÒK/~D"CfffQSSsÜ`^^Þ~~ðÁ999¦dßK_ì§ì;EcÆÙ³gy8	<òÈðáÃ£¿óüÄô×¾ðíÛ·ÇO?ý´_-ê÷¤?>¦¦¦=z46ìììLJJ2K ûªØ[úz;ëÑ/¿úÕ¯&OlNÂêÕ«.ÐÐ~²îÜ¹sÞ¼yÿöoÿfZúå?ÿó?gÍU__øðá7^uÕU)))¦åT²/99ùÈ#]³/ YÙ7TÅÞÒ'ûNùY±by8	×ß©ªªºâ+LK=úè£cÇi2sæÌW^y%SÉ¾ÜÜÜ®y[[[~Â ûÒ9î¸ÌÌÉù¿øGyÄ<#FtvvÆkLË©Ø¼y³_é8Åì5kVmmmlXSSzÚ,ìÛ³ýrÍ5×lÙ²Å<É'·¶¶Æágí¤ILK¿,°ë+¦?øÁ/^lZNåøÃþ°ëõüä'>ø YÙ'ûøX^^È=9O>ùdEEEUUÕïÿû0·Ür÷öõW·èG4øá6l¸üòËÿçþÇ´Ê	pÿþýá??6nÜØÖÖöÊ+¯ìÚµË,ì|,%%¥ë'hÐ/=ôPø±þ×_Á:9¡óóó³³³¿øÅ/¾õÖ[&äÔO?ÿùÏ¢OËþô§¦d²Ù ûÈ>d²Ùì@ö ûd²Ùì@ö ûÈ>Ùgàl¶uëÖ¹sç=:{íµ6lèmÏç¾¬¬,'''11177÷;îØ±cÇ§ßê8qÓ¦MÓ§O7ó²87oðÇôÑ>þøã	=dddTUUõ7ûN|Odpz»ã;öîÝðàÁU«VaqqqÏ=KJJÂ¦ûî»o×®]®¬¬¼îºëÂôû¤ ûd0ÀBºeggïÞ½;:lkkAÜsÏÔÔÔ°iûöí±5;vìkÂÝÆÛ¶m!+ÓÓÓ³²²***cb¢û×ÔÔG"ÌÌÌÐuuuÝqýúõ&LHII>zx´/¿üò¸qãÒÒÒn¸áØÎÙ|*äTh¬+¯¼²ç¦ùóçM·Þzë³Ï>ÛÑÑÑý[½GöJëZxK,9nö566æäät]9zôèXÌE×><¶õóÿ|´A£æÎÝ³ïã ûÿéâ/µ´fÍ[&M+ªH$òµ¯mçÎd_YYYmmmKKË½÷Þ½KÏ=eËá3>QZZK.íºóW¾ò®]»6:xqFßny"Ç@ö«®®.**´hÑ¢Þö9räÈO>ùÅ/~1öb[ZZÚ«¯¾Ú[öÅ®±677á°aÃùùùaXYYVUUa^^^×Ãrt¸k×®0<tèP×CõdðmÛ¶5*ú*Zggçgîÿá®[·îúë¯^rí-ûb=ÚskìhÉÉÉa.:¶]RRR×CqvÆd×CõdëÞ|óÍ¬¬¬PH«V­:vìXo»E¯ÿ¾óÎ;±5kRSSË¾?:ô¾µ   ë«ta!»sÃ¾ìsÚ¾û.¼ðÂGßýîwûÞsùòåa·)S¦lß¾ýðáÃ_ûÚ×Â	&ô7û¢ÐÒÒK,	ÃÒÒÒ®ïÉÿ³¯ïã ûàöo|#áxzî¹wïÞ.¸ ÛnÃûùÏÞßì~`ì3ÿêêê233»677·©©©¿Ù×÷qpN»è¢N0ûÚÚÚ/ùË^xabbbNNNYYÙ/¾xÜë;ûÖ­[W\ZZZ]S]]]^^êëëO¤ózû8²Ùì@öÈ>d²Ùì@ö ûÈ>Ùì@ö û<ÿKÞ"%IEND®B`


3 Sintomo * n. sintomi * Esito dopo


Test del chi-quadrato	
Esito dopo	Valore	df	Significatività asintotica (bilaterale)	Sign. esatta (bilaterale)	
1	Chi-quadrato di Pearson	3,000b	1	,083		
	Correzione di continuitàc	,188	1	,665		
	Rapporto di verosimiglianza	3,819	1	,051		
	Test esatto di Fisher				,333	
	Associazione lineare per lineare	2,000	1	,157		
	N di casi validi	3				
2	Chi-quadrato di Pearson	.d				
	N di casi validi	8				
Totale	Chi-quadrato di Pearson	11,000a	2	,004		
	Rapporto di verosimiglianza	6,702	2	,035		
	Associazione lineare per lineare	9,290	1	,002		
	N di casi validi	11				

Test del chi-quadrato	
Esito dopo	Sign. esatta (unilaterale)	
1	Chi-quadrato di Pearson		
	Correzione di continuitàc		
	Rapporto di verosimiglianza		
	Test esatto di Fisher	,333	
	Associazione lineare per lineare		
	N di casi validi		
2	Chi-quadrato di Pearson		
	N di casi validi		
Totale	Chi-quadrato di Pearson		
	Rapporto di verosimiglianza		
	Associazione lineare per lineare		
	N di casi validi		

a. 5 celle (83,3%) hanno un conteggio previsto inferiore a 5. Il conteggio previsto minimo è ,09.	
b. 4 celle (100,0%) hanno un conteggio previsto inferiore a 5. Il conteggio previsto minimo è ,33.	
c. Calcolato solo per una tabella 2x2	
d. Non viene calcolata alcuna statistica perché n. sintomi è una costante.	


Misure simmetriche	
Esito dopo	Valore	Errore standard asintoticoa	
1	Intervallo per intervallo	R di Pearson	-1,000	,000	
	Ordinale per ordinale	Correlazione di Spearman	-1,000	,000c	
	N di casi validi	3		
2	Intervallo per intervallo	R di Pearson	.d		
	N di casi validi	8		
Totale	Intervallo per intervallo	R di Pearson	-,964	,037	
	Ordinale per ordinale	Correlazione di Spearman	-,638	,234	
	N di casi validi	11		

Misure simmetriche	
Esito dopo	T approssimatob	Significatività approssimata	
1	Intervallo per intervallo	R di Pearson	-94906265,624	,000c	
	Ordinale per ordinale	Correlazione di Spearman			
	N di casi validi			
2	Intervallo per intervallo	R di Pearson			
	N di casi validi			
Totale	Intervallo per intervallo	R di Pearson	-10,854	,000c	
	Ordinale per ordinale	Correlazione di Spearman	-2,487	,035c	
	N di casi validi			

a. Non viene assunta l'ipotesi nulla.	
b. Viene utilizzato l'errore standard asintotico presumendo l'ipotesi nulla.	
c. Basato sull'approssimazione normale.	
d. Non viene calcolata alcuna statistica perché n. sintomi è una costante.	

È>d²Ùìbìo£ëãm£Ë3gÎ<wé¤ÆÆÆSá²àÊ¾ãØj§Bö:thÎ9§H²àj¬xÊ¾W^yeÒ¤IìN£ìkjj*//ÏÍÍMII?~üêÕ«[ZZºÜ¶Ç	kkkKJJ233322ëëëöG<ûì³S¦LINN8qâsÏ=×å.õrèO?ýôyç½yç#í¶½¿ÀBó=ðÀ²Ài!º´ÑªU«H$++«óÊ#FìÞ½»ûñ7nÜ8hÐ Ýº¼ÆÖûq¢k;ßüÍ7ß<æmÏ¾¯ýëá^ýãù-C@ö·ìë¥¢okxíµ×Z[[×®]GÝ=»RyyyÎ5k÷gÂtéÒ¥ÝïÀüùóÃ¦yóæ½÷Ë/¿¼ó¡z?NtÏÂÂÂ°éý÷ßaºpáÂ/þí&@üdßôéÓÃr^^Þ+Ö­[×ÜÜÜcu)¤aZUUVWWé¨Q£ºßèr_·oßÞùP½'ºgmmmtZSS¦cÇ=æmûø.ÙÈ> Þ²¯êêê&OÜFÃß°aÃ1³/)))L:èÕØîÇîÙÚÚÚyÏCõ~è]¶ó¶²ìëÙ¦Mn½õÖèg3æÙÓù¶°¦¹¹¹Ý<nÜ¸°éí·ßN£¯Øuª÷ãD÷|ã7¢Óp09rä½ÿáÉ@<d_ô5³Gy¤µµ5Ä_XÎÊÊê~Û°DöíÛ¦eeeaZPPÐù÷ê/_Þýø-æÎvï½÷¢¿×qØÞÝsÆ;wîÜ»wïWùwûú~d ûÓ%ûz¹âyõÕWwY¿bÅîUiô½ ²³³÷ìÙÓýTUUE±ËÛr£[?NtM4L;ÿú×¿öå¶²ìûBö577ßrË-¡êRRRÆsóÍ7òÉ'Ý«hÝºuãÇûD×ÔÔÔ6,===,ìØ±ãh÷áüãÄ£¼÷ì³Ïv­^Ýó¥^4iR¸ù´iÓÂrç#÷ý>È>@öêÍê<²@öÈ>Ù ûÈ>dñkÖ¬D"~¢q?ùÉOÞyç?QÙ ûdì²OöÈ>Ù ûdì²OöÈ>dì£OçÙºukaaaZZÚÐ¡CKKKdÄaö%''è¤¬¬ÌAö@f_aaa]]Ý¾ûnºé¦0ÍÌÌtfÙW__655éÀdÄaöµ¶¶F§íííÑ5Î²â0ûz_§Ü¢²²rêÔ©©©©Ó¦MÛ¶m[çM[¶l;vlbbâäÉ7nÜ(û@öÃÙ7aÂ6:tè¾ûî8qbçMÅÅÅ÷ÞossówÞYTTÔ=û~úé­@yâ'æÌÿælãXã÷¿ÿ½GKLx÷ÝwOJöI@NÑìëÐÒÒÒå-H999yyyÝ³oóæÍïqäî»ïrÖ§÷^Fâù7Þx£GKLØ¿¿ìCöõàÏþó%K:¯IJJ:räHXhkkKLLtâÞï~÷»¬oü^FêüÃ_-@¬f_SSÓ7¿ùÍ.ÿWÖÞÞÍ¾²d!ûØÎ¾½÷îÚµ«Ëúüüüè÷Ì466æææÊ>ìb8ûþù3f444tßZðîiiiY³fÍdÈ>Cö1yyy¿Oð_÷ò³ÜÜÜ°Oee¥ìÙgÈ¾Óâ©ºÏoÈO3gÎ<wÙw,[¶ìûÈ>²Ó<ûNPÉ>Ù×¯V¯^-û@ö²Füg__È>²ØÕÔÔT^^2~üøÕ«W·´´t¹èòÖ­[ÓÒÒZZZ¿ã/î_[[[RRQ\_ß¥7lØ0iÒ¤äää3g¾ÿþû/¼ðÂyç:öìÝ»w«IÙ'ûÙ'û8ÎBué¶U«V-ûB¥uÞ³¬¬¬ÇìD"YYYW1¢KÌ4¨cë¢³c:þ|Ù'ûd ûdÇYFFFH«×^­µµuíÚµayôèÑGË¾ÂÂÂºººûöÝtÓMaÚñ¥V]ú¬¼¼<LgÍµû3aºtéÒÎ;_ýõû÷ïþÁâÅÃôáËá.É>Ù'ûÙ'û8Î¦OÒ*//oÅëÖ­knnþü©º[öumjjÓöØg!Ã´ªª*:­®®ÓQ£FuÞ9åCE§ÑÍmiiñÝn²Oö²OöqBÔÕÕM<¹ãëðáÃ7lØp´ìkmmNÛÛÛé³¤¤¤0IFÛ®ãÛM£;·µµu~úé§GûCýdìdìã¸Ù´iÓ­·Þ:gÎYcÆéKõ²5''§ó«a!L;¾æªËÎ½Leìì7ÑWæyäÖÖÖa9++ëËf_BBBôºí¾ûÂ´¬¬,L:ÿnßòåËeìì4W_u·â®X±âËf_~~~tZ\¦õõõÑwtÈÎÎÞ³gì²²¦¹¹ù[n	Ý2fÌo¾ùO>ù²Ù·nÝºñãÇ#D×ÔÔÔ6,===,ìØ±£/'ûdìdìdìdìdìdìdìdìdìÙ'ûd ûdÈ>Cö²²@ö²Oö²Oö²Oö²Oö²Oö²Oö²Oö²Oö²Oöq¼õ¢Ç<$½¾¾Þ¹Bö²OöÃ¦]tiÆÊ'`1ñÎ²²Ó(ûöìÙ³páÂáÃ§§§_sÍ5÷îueìdì#³ïâ/¾ë®»öæ?üáìÙ³CÙ'ûÙ'ûÃìëìÀ©©©Î¡ììÄmö9rä>¸ûî»-ZäÊ>ÙÈ>ÙGÜf_QQQzzzZZÚæÍCÙ'ûÙ'ûÛìÚÚÚV®yòdçPöÉ>@öÉ>â9û>ú())É9²²8Ï¾ÆÆÆ¼¼<çPöÉ>@öÉ>â0ûÎ?ÿüÇ¬µµõàÁ?úÑ~úÓ:²Oö²OöÙW__?oÞ¼ôôôÛn»íÓO?ueìdì#,øÖ±¿7Õwò"ûÙ'ûÙ'ûÙ'ûÙ'ûÙ'ûÙ'ûÙ'ûè7÷ýßÿ]qõÕ½oÿÏÿ<xÐ¹Bö²OöÃ¦þ×4iÀÅ÷2Ñãçö!ûd ûd1sçX¼¸9~¼ìCö²OöqfßË/¿<`À'PöÉ>@öÉ>â<û®¼òJÙ'ûd ûdq¯½öÚw¾óÙ'ûd ûdqÅÅÅûÛßdììÄsö½õÖ[sæÌùç¿ì²²8Î¾ë®»îøì²²xÎ¾ººº)S¦üë_öÉ>ÙÈ>ÙG¼f_qqñ/reìdì#³ïOüOöÉ>@öÉ>bÅ×§O~ÖY#§Mëe6ìÕW_È>@öÉ>bØë¯¿þ»cyüñÇ(d ûd ûd ûd ûd ûd ûd ûd ûd ûd ûd ûd ûdÈ>Ù'ûÙ'û@ö²ì²²²²²²²²²²²²²d¶ì ûdìd ûdìdìdìdìdìdìdìdìdìdìdìdìdìÙgÈ>@öÉ>²Oö²²@ö²Oö²Oö²Oö²Oö²Oö²Oö²Oö²Oö²Oö²Oö²Oö§WöE"ìììîëCÕèDöì3dÃÙ÷äOæççw¯ºàÁ,++;ÚeÈ>ÙKÙ7oÞ¼íÛ·÷K,yè¡zÉ¾ûï¿ÿI ¬rèôkÝ1³oÑ¢E-1áW^QÈ¾nw«§ì3fÌ¥^>úô^í¯ö^íâ3û¢ÚÛÛ«««/¼ðBÙ²Ï@<g_´üÒÒÒdÈ>Cöñ&LØºuk[[Ûo¾9wî²Ï@¼e_tºyóæ)S¦¤¥¥F"Ù²Ï@Ìg_Ë-;æ>²dìùì[½zµìÙgÈ> þ³¯/dÈ>Ù ûÙ'ûÙ'ûÙ'ûÙ'ûÙ'ûÙ'ûÙ'ûÙ'ûÙ'ûÙ'ûÙ'ûÙ'ûÙ'ûÙ'û@ö²²dììììììììììììììì ûÙÈ>?Q²@ö²Oö²Oö²Oö²Oö²Oö²Oö²Oö²Oö²Oö²Oö²Oö²Oö²Oö²Oöì3d ûÙ'ûd ûd ûd ûd ûd ûd ûd ûd ûd ûd ûd ûd ûdÈ>Ù'ûÙ'û@ö²ì²²²²²²²²²²²²²dìì ûÙôsöíÞ½éÒ¥£FJLLÌÍÍ]¾|ù=d ûdWÙWWW7bÄ_½sçNÙÈ>ÙÄOöÎ+((¨®®>tèPUUÕ¬Y³ÂÙÈ>ÙÄOö¥§§ÈÛ½wÇ°ÖdddÈ>@öÉ> ~²/33³Çìëe ûd?Ù·`ÁèEÞªª*yÙ'û¸Í¾ÚÚÚ¬¬¬.oé:thMMìdìâ'ûH$R^^>zôè¤¤¤%KìØ±£ÿMdÈ>ÙpÂ³ïT û@öÉ>Ñ¹=ììì@,dß©Föì'6ûöRß!CÎ;ï¼ÿüç~ú©ìdìâ6û:¬^½Zö²Oö1»ví0aÂe]V]]ÝÒÒRUU5cÆ.ºèÃ?üå/²oÜ¸q²²ùì=v/gÛ¹sgXsÍ5×ìÛ·/,$%%É>@öÉ> æ³/999´Ý=:ÖìÝ»7ú»­­­²²ì;çsBÛ]qÅµµµ~ûí·ÃrXsÖYgmØ°!,;Vö²Oö1ë×¯<xp·q5?þxtyÕªU²²ùì^õÕ«®ºê3ÎHHHÈÉÉùÖ·¾µeË°~È!¥¥¥ÍÍÍ²²ì;È>²àÄfkdìNëìóqÍì@kdìNìóqÍìÀi>®²8-²ÏÇ5²Oö§Eöù¸f@öÉ>à´È¾ø¸f@öÉ>à4É¾SìÙ'ûNxö½üòËÅÅÅÙÙÙ			a:mÚ´'|Rö²OöqO=õÔ A:~«ïÿÿûÛßÊ>@öÉ> ~²oâÄ!ò~úéì[»vmX8óÌ3e ûd?Ù"/úÑìÛ¿XHIIì@üdß¹ç"ï±Çf_ccã²eËÂÂÔ©Se ûd?Ù÷Ì3Ïôø¹ë×¯ì@üd_ðÆo{íµãÆKNN=ztIIÉ¦MúùßDöì'6û:~¥¯Ã¾û/»ì2ÙÈ>ÙÄsöE"°&55Uö²Oö13gÎÐ«³Î:ëß;r¨Æìììîë·lÙ2vìØÄÄÄÉ'oÜ¸Qöì3dÐÙWQQq´àKHHÈÏÏüñÇÿÃ>ùäá¶]^>*..¾÷Þï¼óÎ¢¢¢îÙºðbÁ/¾ø8Ç²iÓ&5kÖ¿d°;föÝtÓM-1¡±±QyÙ÷ù!º]äýOÌ7oûöí=0''çÀa¡¡¡!//¯ö=õÔS[ÎÈË3zi£G%;ÛCå¶ÛnvñµÂîÙwýõ×´Äð§<íìÿï²xñâÌÌÌvyÙï?IÉî+9ÚÚÚ]äá]°x±ÑÛ()ùJO¿êà"¯á"/p2³¯´´ôhWoö¥¥¥µ··G³/$ ì²Oö²è×ì:thxÿÁ~ÐÔÔÍ²ãÝWæçç?"úúbnn®ì²Oö²è×ì2dHxÿè£oôø²â=÷ÜÓÒÒ²fÍÈ>Ù'ûd!û~Í¾9sæ'ñ;v¸ìN+**rssòòò*++eì²Ï@¿fß-[/¿üòúúúÖÖÖq/-[vÌdì²OöØìëå×½zµì²Oö²ÿìëÙ'ûdì'6ûN²OöÉ>Ù'ûNxöíÞ½éÒ¥£FJLLÌÍÍ]¾|ù=d²OöÉ>ÙÄUöÕÕÕ1¢ËåÝììì;wÊ>dì²ì+**OâÕÕÕªªª5kVXSRR"û²OöÉ> ~²/===<ïÞ½»cMXk222d²OöÉ>ÙÄOöefföa½ìCöÉ>Ù'ûøÉ¾D/òVUU¹Èì²OöqµµµYYY]ÞÒ1tèÐÙì²OöñA$)//=ztRRRNNÎ%KïWôÊ>Ù'ûd!ûS"ûN²OöÉ>Ù'ûNTöýå/¹è¢***:¯ÓË.»¬ººZö!ûdì@<d_mmmZZZxú9sfçõaV9òÝwßÈ>Ù'ûdóÙWVV»óòò*++;¯ùåG6­Rö!ûdì@ÌgßèÑ£Ãs÷óÏ?ßÓ3Ï<6yæ²Ù'ûdìb>ûÂs÷ÇÜÓÁÃ¦ÔÔTÙì²Oö1Ñ/çØ¹sg÷M»ví&û²OöÉ> æ³/úU?þñ»oºýöÛÃ¦K.¹Dö!ûdì@Ìgß#<»Ê¯¶¶¶µµõðáÃÛ·o¿õÖ[ÃÊ°éÁÈ>Ù'ûdóÙ¬rÀQ,[¶ìÈ#²Ù'ûdìâ!û^z©´´tÜ¸q)))¹¹¹.ìñí½²Ù'ûdìb8ûN²OöÉ>Ù'ûd²OöÉ>ÙÈ>Ùì²Oö²Oö!ûdììÈ>Ù'ûd ûd²OöÉ>ÙÈ>Ù'ûì²²Oö²OöÉ>@öÉ>Ù'ûd!ûÙ'ûdì²@ö!ûdììÈ>Ù'ûd ûd²OöÉ>ÙÈ>Ùì²Oö²Oö!ûdìì²Ï²Oö²OöÉ>Ù'ûdìdì²Oö²~¢²OöÉ>Ù û²OöÉ>@öÉ>dì²²Ù'ûdìdìCöÉ>Ù'ûÙ'û²OöÉ>@öÉ>ÙgÈ>Ù'ûÙ'ûdì²Oö²OöÉ>Ù'ûÙÈ>dì²@ö!ûdììÈ>Ù'ûd ûd²OöÉ>ÙÈ>Ùì²Oö²Oö!ûdìì²Ï²Oö²OöÉ>Ù'ûdìdì²Oö²È>Ù'ûdìCöÉ>Ù'ûÙ'û²OöÉ>@öÉ>dì²²Ù'ûdìdìCöÉ>Ù'ûÙ'ûd!ûdìdì²OöÉ>ÙÈ>Ù'ûdì3d û²OöÉ>Ùì²Oö²Oö!ûdììÈ>Ù'ûd ûd²OöÉ>ÙÈ>Ùì²Oö²OöÉ>CöÉ>ÙÈ>Ù'ûdìÓv²²OöÉ>Ù'ûd û²OöÉ>Ùì²Oö²Oö!ûdììÈ>Ù'ûd ûd²OöÉ>ÙÈ>Ùì²Oö²OöÉ>CöÉ>ÙÈ>Ù'ûdì3d ûdì²OöÉ> ³oË-cÇMLL<yòÆ;oU7 Ù'ûdì3dÃÙW\|ï½÷677ßyçEEE7=øàeee½¼Ú·~ýú×²¯/Ùù¯x¨ÜqÇÃ.¾VØ3ûÊËË=ZbBMMò@ö.''çÀa¡¡¡!//¯ó¦%K<ôÐC½dßæÍ#ÄÙ×ìË9ÒCå®»îÊ¼d°;föÝxã-1áÃ?TÈ¾Ï%%%9r$,´µµ%&&vÞ4fÌK/½4==úôéÛ·ow×E^y]ä5b8ûÒÒÒÚÛÛ£Ù°ûakuuõ^(ûdììb8ûòóóÂBcccnnnûòu(ûdììb8ûJKKï¹ç5kÖ,X° ó¦	&lÝºµ­­íÍ7ß;w®ì²Oö²áì«¨¨ÈÍÍMHHÈËË«¬¬ì¨ðÏÍ7O2%--­°°0È>Ù'ûd!ûÎ¾-[¶ìûÈ>Ù'ûdìùì[½zµì²Oö²ÿìëÙ'ûdì²Ù'ûdìdìCöÉ>Ù'ûÙ'û²OöÉ>@öÉ>dì²²Ù'ûdìdìÂNöÉ>ÙÈ>Ù'ûÙ'ûd ûdì²Ïì²OöÉ>Ù û²OöÉ>@öÉ>dì²²Ù'ûdìdìCöÉ>Ù'ûÙ'û²OöÉ>@öÉ>ÙgÈ>Ù'ûÙ'ûdì²Oö²OöÉ>Ù'ûÙÈ>?QÙ'ûdìÈ>Ù'ûd ûd²OöÉ>ÙÈ>Ùì²Oö²Oö!ûdììÈ>Ù'ûd ûdì3dìì²OöÉ>Ù'ûÙ'ûdììd²OöÉ>Ù û²OöÉ>@öÉ>dì²²Ù'ûdìdìCöÉ>Ù'ûÙ'û²OöÉ>@öÉ>ÙgÈ>Ù'ûÙ'ûdì²Oö²OöÉ>Ù'ûÙÈ>dì²@ö!ûdììÈ>Ù'ûd ûd²OöÉ>ÙÈ>Ùì²Oö²Oö!ûdìì²Ï²Oö²OöÉ>Ù'ûdìdì²Oö²È>Ù'ûdìCöÉ>Ù'ûÙ'û²OöÉ>@öÉ>dì²²Ù'ûdìdìCöÉ>Ù'ûÙ'ûd!ûdìdì²Oöi;ÙÈ>Ù'ûdì²È>Ù'ûdìCöÉ>Ù'ûÙ'û²OöÉ>@öÉ>dì²²Ù'ûdìdìCöÉ>Ù'ûÙ'ûd!ûdìdì²Oö²²OöÉ>Ù'ûd û²OöÉ>Ùì²Oö²Oö!ûdììÈ>Ù'ûd ûd²OöÉ>ÙÈ>Ùì²Oö²OöÉ>CöÉ>ÙÈ>Ù'ûdì3d ûdì²OöÈ>dì²@ö!ûdììÈ>Ù'ûd ûd²OöÉ>ÙÈ>Ùì²Oö²Oö!ûdìì²Ï²Oö²OöÉ>Ù'ûÙÈ>Ù'ûdì²Ù'ûdìÈ>Ù'ûd ûN-[¶;611qòäÉ7nìã&Ù'ûdì1ÅÅÅ÷ÞossówÞYTTÔÇM²OöÉ>Ù'ûb,ûrrr8òòòú¸)E8cÆË	ÉÉ1z©©i*S§N8lÑËvöÙg´Ä+V(dßç9ÚÚÚû¸ØòÑG½Ã±<xÐC%D"ÇäqÄdö¥¥¥µ··GÛ.t^7cÙßÔÔsssû¸	Ë¾ÒÒÒî¹§¥¥eÍ5,èã&b,û***rssòòò*++ÿu/8Ú&b2ûz´lÙ2?*øÏ¾Õ«WûQÄö ûl7n>zZZÚ¹çß÷9!pbÿNÿ¢A9'ì£?lÙ²å+®xë­·ZZZ*++Ï>ûìûï¿ßiþñâ/~÷»ßuÙGøÆ7¾D:¦7o>çsè3fÌxûí·@öq´´´$'';Ð^ýõ3g:ìãä¨©©4ióý`ÕªU¿ùÍo@öqrÜqÇÞÕýcÌ1÷îuÙÇIðÎ;ï´··;p¢½õÖ[S§NuÙÇIpøðák¯½¶±±Ñ©~ðë_ÿzÅÎ ûèoíííßÿþ÷ßxã§úÇ÷¾÷½xÀydýê>(--Ý¶mSýæ¢.Ú´ióÈ>úOeeå×¾öµ§úÓ¨Q£:d&ìã?~ün8Ñ[ZZ@öÈ>d²Ùì@ö ûÈ>Ùì@ö ûÈ>d²Ù ûÀIôè£^|ñÅiiiIIIùùù?ûÙÏ9ÒãÏ=÷öu×]·ûöÏÿËÿL_þÄ3g:ó²è?¿úÕ¯tsûí·wßóáî¾gzzzuuõÍ¾¾ï	ìÂÂÂ_üâMMMzì±ÇBeeeuß3???lºå[víÚuøðáªªªK.¹$¬)..þÒGÈ>Ù,ííí¡ÿBwÞyÝwHII	¶mÛÖ±fûöíaÍ°aÃºÇëÖ­!+ÓÒÒZZZÊ²cSèþµµµ%%%¡ACGÖ××wiÄ6L4)99yæÌï¿ÿþ/¼îdjjêìÙ³wïÞÝ±s/Ç@öÿú)XÙÙÙ!×ºïPTT¶&$$Í5ë×¯ommíú_~·ìÖ¹ðÊÊÊzÌ¾H$Õyå#:b.ºfÐ A[/¸àhFÍ??ºgïÇ@öÿ4zôèh*>¼²²²ûöì9ÿüó;*33ónØ¹sg/ÙWXXXWW·oß¾nº)zîåååa:kÖ¬Ý)((Ó¥KvÞùúë¯ß¿ÿÚµk£ÓÅiô×322úrdðO¡ÏBNT:ÚÛlÛÚÚôÑ+¯¼²ãÅ¶ÔÔÔ_~ùhÙ×qµ©©)LØcöE³ªª*:­®®ÓQ£FuÞ9åCE§»víÓÎêý8È>àsÑ÷çö¾ÛÇ¼nÝºK/½4zÉõhÙ×q!¸½½½ûÖ£%%%iHºè4ÚvwÅÙyúé§v?TïÇ@öÁiíª«®:ã3þþ÷¿G§ï½÷^çË¦;6lzã7:Ö|ôÑGaMJJÊÑ²ï/kNNNçWéÂBæææö¸s/ÓÞìÓÚÂCÍ;7ßÞ½,X¦EEEÝ÷|yØ4mÚ´mÛ¶>|8ÜpÃaÍ¤I¾lö%$$D¯ÛîÛ·/LËÊÊÂ´   óïä?îËf_ïÇ@öÁi­®®.==½ó»_VSSÓÏ#GvyîÀxâ/ÑìøÌ¿úúúÎÍÎÎÞ³gÏÍ¾ÞìÓÝ¶mÛæÌ©¨¨¨ó÷­uoÄoûÛ_ýêW²²²ÿô§?õX`½gßºuëÆRPP]B³¤¤$ghÐ°°cÇ¾t^÷i/Ç@ö û²Ùì@ö ûÈ>d²@ö ûÈ>dýèÿÁØxý)IEND®B`


þª²Cö¢ZíÔf_mmmø¯_sÍ5¸ã;ÂáèÑ£ýUdóìëììhQeee^^^¨Ûo¿½«««×Û÷ËÍÍÍõõõ%%%EEEuuuÛ·oïë?ñäO^pÁ?~üSO=Õë]êç<É._¾|Ü¸q¹¹¹É7O?s_oÛÏ8§N:xðà]»v%ïyèÐ¡p8¹¿*ìb!zµÑ-·ÜòÙH$JKKÓ¯:thæù×®];`ÀÔÝþ.õä5999éo¾uëÖ|Û]û¹ç×*õW@²¯***_~ùå%KËåååÉØ+-Z§MÖþ555ápáÂïÀWn5kÖÎ¸üòËÓOÕÿy÷¬­­7íÚµ+ùúìÜ¹s?ìûÐðV#GoõÛßþÖ_@ö1Ï¾.º(>|ø7Þ¸lÙ²özÛãf_HÃpØÐÐ<lllÃË|_K½üÚÔÔ~ªþÏ¼gsssòpÛ¶má0Ú¾í|µ/-ü¯W^íµþ²Iöõs'¦ÂhÈ!+V¬øÀìËÍÍÝÝÝÉÃp!ùjlæù÷ìééI¿gêTý'yÏ^·¦¾¯·ýÀìÛ¸qãYg®¹îºëÞï=OÙÄ?û^|ñÅ;î¸cÆáÎ#FøÀì«¨¨HÿJ[¸+++3Ï<jÔ¨pÓ«¯¾<L~Å.uªþÏ¼ç+¯¼<'	gözyùåÃÃÿäcÇùKÈ>à´È¾ä×Ì.]ÚÓÓâ/--Í|ÛäïºK$ûöíóçÏ555éßW·xñâÌóõ«_7Í93ÜmçÎÉïÏK¶ÿó$ïYUUÕÚÚºgÏ+¯¼2ýûNüH÷æosÎ9á?øÁüõd·ìëçÏ/éK½®¿ñÆ3³oÌ1ÉÃºººp¸ûöäÏ¤íÞ½;óhhhHÿõÈ©ËMÞÚÿy×$Ã4uùÏþó¼m_n½õÖïWÈ> ÚÙwðàÁÛn»-T]^^Þ#¾ùÍo>|83û-[6zôèpä5Û¶m«¯¯<xpaaa¸°cÇ¾ÞU«V?>ù÷|òÉ^ÕÏy÷zõ	ÂO2%?ó¿)úÔ§d û>Íêqdì²@ö ûÈ>bwßw"ðÇóìûÞ÷¾÷úë¯ûcÈ>Ù'ûdì²@öÉ>Ù'ûdìÈ>dì²OöÈ>Ù ûdì§eöuww/Z´hÈ!cÇ]»v­ìgöÝyçßþö·>¼lÙ²P~Ù·|ùòQöôWüÿ?YT>f£òô²sçÎ(eß¸qã6oÞÜ×­!û^zé¥Q6sé!ÿÝafQYøÊÓËÛo¿¥ìËËË»óÎ;'L°eË/òñóÅßïóyÔ,B³^äýX8ðî»ïîêêZ½zõç>÷9ÙÈ>3ñÌ¾ä×'ß÷Ý¼¼<ÙÈ>3ñÌ¾ë¯¿þW¿úUh¾gyfêÔ©²f&ûâû÷ï;wnaaá¤Iþú×¿Ê>@öìgöõOö²ÏÌdìf&ûdì33Ù'ûdÉ>Ù ûÌdì²ÏLöÉ>Ù ûÌdìì33Ù'ûd ûÌLöÉ>ÙÈ>3²@öì²ÏÌdìf&ûdì3²OöÈ>3Ù'ûdì3²Oö²ÏÌdìì33Ù'û|ÎdÉ>Ù ûÌLöÉ>Ùgf²OöÈ>3²@öÉ>Ù'ûdì²@öÉ>Ù'ûÙgf²OöÉ>@öì²ÏÌdìf&ûdì33Ù'ûdÉ>Ù ûÌdì²ÏLöÉ>Ù ûÌdìì33Ù'ûd ûÌLöÉ>Ùgf²OöÈ>3²@öì²ÏÌdìf²OöÉ>Ùg&ûdìf²OöÉ>@öì²f&ûdì33Ù'ûdÉ>Ù ûÌLöÉ>Ùgf²OöÈ>3Ù'ûdì3²OöÈ>3Ù'ûd ûÌLöÉ>ÙÈ>3ñË¾PuYid ûÌLöÅ3ûî¿ÿþùóç÷óÕ¾-[¶tDÙ¬ßôyÔ,B³QyzéêêRö-X°àè'û~øáµQVuo«Ï£fZøÊÓKÿ/~â²oÄ^ziaaáE]ÔÔÔäE^À¼fæEÞx¾ÈtôèÑÆÆÆ/¼Pö²ÏÌd_³/Y~²f&ûâçwÞÆßÿý­[·Î9Sö²ÏÌd_<³ï¥^ºàjkkìdÉ¾xf_ÿd ûÌLöÉ>Ùgf²OöÈ>3²@öì²ÏLöÉ>Ù ûÌdì²ÏLöÉ>ÙÈ>3²Oö²ÏÌdìì33Ù'ûdÉ>Ù ûÌLöÉ>Ùgf²OöÈ>3Ù'ûdì3²OöÈ>3Ù'ûd ûÌLöÉ>ÙÈ>3²Ïç@öì²ÏÌdìf&ûdì33Ù'ûdì²@öÉ>Ù'ûdì²f&ûdìdÉ>Ù ûÌLöÉ>Ùgf²OöÈ>3²@öì²ÏLöÉ>Ù ûÌdì²ÏLöÉ>ÙÈ>3²Oö²ÏÌdìf&ûdì33Ù'ûdÉ>Ù ûÌLöÉ>Ùg&ûdìf²OöÉ>Ùg&ûdìdÉ>Ù'ûÙgf²OöÈ>3²@öì²ÏÌdìf&ûdì3²OöÈ>3Ù'ûdì3²Oö²ÏÌdìì33Ù×ì[±bEVVìdÉ¾8g_ww÷øñãe ûÌLöÅ<û¾ûÝïþð?ì+û~øáµQVuo«Ï£fZøÊÓKÿ_ûÄeß;&NxäÈ¾²oË-Q6ëá75ÐÂÇlT^ººº¢W]uÕªU«þþyð"¯y7Æ/òfý+ÙÈ>3±ýÞ¾Tÿùj ûÌLöÉ>Ùgf²/Ùwf&ûdì33Ù'ûdÉ>Ù ûÌLöÉ>Ùg&ûdìf²OöÉ>Ùg&ûdìdÉ>Ù'ûÙgf²OöÉ>@öì²ÏÌdìf&ûdì33Ù'ûdì²@öÉ>Ù'ûdìUöµ··/pØ°a999/Þ½·ìf²/VÙ×ÒÒ2tèÐ¬UVVÖÚÚ*ûdìOöÍ=;t^MMMcccwwwCCÃ´iÓÂ5õõõ²@öÉ¾ød_aaa¼öööÔ5ár¸¦¨¨HöÈ>3Ùì+))9nöëeì3ñÉ¾9sæ$_ämhhð"/ì3±Í¾æææÒÒÒ^?ÒQ\¼mÛ6Ù ûÌd_|²/H$-*//ÏÍÍ­¨¨X°`Á;>wWö²ÏÌdß©Ì¾Fö²ÏÌdßÉÎ¾ä¹©Ç%ûdì²f&û¢§ìdÉ¾S©ïÌ3Ï7nÜüã÷ÞOöÈ>3ÙÛìK¹ýöÛeì3Ï¾¶¶¶óÎ;oúôé]]]UUU_|ñ[o½õÓþ4dß¨Q£dì3Ï¾Ë.»¬×?ÎÖÚÚ®¹úê«÷íÛ.äææÊ>Ùg&û"m·÷îÔ5öìI~o_OOìf²/&Ù÷Ï|&´ÝW^ÙÜÜ|äÈW_5×î¹+V¬F)ûdì|ö=ñÄìõcáG4yù[n²ÏLöE>ûçþª«®:ë¬³²³³+**¾üå/oØ°!ægÎ7ïàÁ²@öÉ¾8dßI#ûÙgf²ïe_× ûÌdßi~]3ì3±Ê>¿®@öÉ¾Ó"ûüºfÙg&ûNìóëdì;-²Ï¯kf²ï´È>¿®@öÉ¾Ó"ûþæ×5È>3ÙwdßI#ûÙgf²ïTfßºuëêêêÊÊÊ²³³Ãá)Sì1Ù ûÌd_¬²ïñÇ0`@ê»úþö¿¿Àù¾ûî²ÏLöÅ'ûÆ"oùòå©ì[²dI¸0vìXÙ ûÌd_|²/777D^òWô%³ïÀáB^^ìf²/>ÙwþùçÈäGÙ·wïÞn¸!<y²ìf²/>Ù·råÊãþÞ¾'xBöÈ>3Ùì^yå¯|å+£F4hPyyyý/¾øq¼»²f&ûNYö¥¾¥/eß¾uuuÓ§O²ÏLöÅ9ûD¸&??_öÈ>3Ùùì«®®Îê×¹çûo¶±±ñ¢.ÊËË8qâêÕ«e ûÌLöâì[³fM_Á=fÌGôß8íäÉ|ðÁ#G¬²¬¬,3û6lØ° Êf.Ýãó¨Y>f£òôrðàÁý"ïÿ]OOO¿Ì_²ïñÇßeÕ÷·ù<j¡Ù¨<½$7ûöîÝÝu×qÆ½¾ì÷ï0jqqq8Û<àE^À¼fæEÞSü"oÊ¼yóúzµ÷ÿrÚ%KË>@öìû¤d_qqq(¼oë[Gý¨Þ³Ceþ,°ìdÉ¾SgyfÈ¾ýû÷$ïÐ§?ýéç¾§§gÙ²e³fÍì33Ù÷IÉ¾3fìÛ±cÇGòmÜ¸qÒ¤I3gÎÌü¶DÙÈ>3§,û6lØ0hÐ Ë/¿|ûöí===ë»+ûÙgf²ïe_?¿±YöÈ>3Ù'ûd ûÌLöE*ûN&ÙÈ>3§2ûÚÛÛ.lØ°ÊÊÊÅïÞ½[öÈ>3Ù«ìkii:th¯wËÊÊZ[[eì3ñÉ¾Ù³gÎ«©©illìîînhh6mZ¸¦¾¾^öÈ>3Ùì+,,×ÞÞº&×É>Ùg&ûâ%%%ÇÍ¾p½ìf²/>Ù7gÎä¼^äf²/¶Ù×ÜÜÚëG:·mÛ&ûdìOöDbÑ¢Eååå¹¹¹,ø¨þ^ÙÈ>3 ì;id ûÌLöìÛ¼yóÅ_¼fÍô+ÃáôéÓeì3qÈ¾æææ¬¬¬êêêôëÃa¸òì³Ï~ã7dì3Ï¾ùóç¼>|øúõëÓ¯_·nÝ°aÃÂM7ß|³ìf²/òÙW^^Úîé§Î¼iåÊá¦±cÇÊ>Ùg&û"ÙÙÙ¡íÞyçÌ:nÊÏÏ²ÏLöE>ûÿ8GkkkæMmmmá¦ÁË>Ùg&û"Éã;ßùNæMwÝuW¸iêÔ©²@öÉ¾ÈgßÒ¥KCÛ80_sssOOÏ#Gî¸ãpe¸éþûï²ÏLöE>ûo¾9«7ÜpÃ±cÇdì3qÈ¾`õêÕóæÍ5jT^^^NNNeeåÜ¹sûã½²@öÉ¾gßI&ûÙgf²OöÈ>3²@öì²ÏÌdìf²OöÉ>Ùg&ûdìf²OöÉ>@öì²f&ûdìdÉ>Ù ûÌLöÉ>Ùgf²OöÈ>3²@öÉ>Ù'ûdì²@öÉ>Ù'ûÙgf²OöÉ>@öì>g²ÏÌdìf&ûdì33Ù'ûdÉ>Ù ûÌdì²ÏLöÉ>Ù ûÌdìì33Ù'ûd ûÌLöÉ>Ùgf²OöÈ>3²@öì²ÏÌdßI²~ýúÉ'çççO2eÓ¦M²f&ûâçwÞ+º»»ùË_?^ö²ÏÌd_<³/¥«««¤¤$3û~÷»ß=¿Z¾îÛÿo£Eh'íùaêo^óyÔ,B³OGDKKK$³ïgY°`Afö555¼°ßÇY´vÒ®|d¯GÛ,B³#âÝwß^öuvv~ñ_<pà@t_äýÏïø81Ö¼Èkf^ä=ÙöìÙ3oÞ¼¶¶¶ÌdÉ>31É¾§~ºªªª£ãøÏ¿²ÏÌdÉ¾dßðáÃ³ÒÈ>3f&ûbþ¼¾Úgf²ÏÌdìf&ûÌLöÉ>Ùgf²ÏÌdìf&ûÌLöÉ>Ùg&ûdÉ>Ù'ûÌdì3²OöÉ>3Ù'ûÌdìf&ûdì²ÏÌdÉ>Ù'ûÌLöì²ÏÌdÉ>Ù'ûÌLöì²ÏÌdÉ>Ù'ûÌdì33Ù'ûdìf²OöÉ>Ùg&ûdì²ÏÌdì3²Oöì33Ù'ûdÉ>3²Oöì33Ù'ûdÉ>3²Oöì33Ù'ûdìf&ûdì3²ÏLöÉ>Ù'ûÌdì3²Oöìf²OöÉ>3f&ûdì33Ùgf²OöÉ>3f&ûdì33Ùgf²OöÉ>3f&ûdì3²ÏÌdìf²OöÉ>Ù'ûdìf²OöÉ>3²ÏLöÉ>Ùgf²ÏÌdìf&ûÌLöÉ>Ùgf²ÏÌdìf&ûÌLöÉ>Ùgf²ÏÌdìf²Oöì²ÏLöÉ>3Ù'ûdì3²ÏLöÉ>Ùgf²OöÉ>Ù'ûÌLöì²ÏÌdÉ>Ù'ûÌLöì²ÏÌdÉ>Ù'ûÌLöì²ÏLöÉ>3²OöÉ>Ùg&ûdìf²OöÉ>Ù'ûÌLöÉ>3Ù'ûdÉ>3²Oöì33Ù«ìK$eee²ÏÌdÉ¾8gßc=6fÌ¬¬¬¾²oíÚµ¯EÁm«ÚEk'íùaÆ]m³-|Ì¾QÊ¾Y³f555õ«V­Ú·®ØáãÄ,Z;iÏ5¿óÍ¢´ð1»5":::¢÷½ýdyÍÌ¼fæEÞøüHì33Ùgf²OöÉ>3f&ûdì33Ùgf²OöÉ>3f&û¢Aöì33Ù'ûdÉ>3²Oöì33Ù'ûdÉ>3²OöÉ>Ùg&ûdìf²OöÉ>Ù'û|É>Ùg&ûdì33Ùgf²OöÉ>3f&ûdì33Ùgf²OöÉ>3f&ûdì33Ùgf²OöÉ>3f&ûdì3²ÏLöÉ>Ù'ûÌdì3²Oöìf²OöÉ>3f&ûdì33Ùgf²OöÉ>3f&ûdì33Ùgf²OöÉ>3f&ûdì3²ÏÌdìf²OöÉ>Ù'ûdìf²OöÉ>3²ÏLöÉ>Ùgf²ÏÌdìf&ûÌLöÉ>Ùgf²ÏÌdìf&ûÌLöÉ>Ùgf²ÏÌdìf²Oöì²ÏLöÉ>3Ù'ûdì3²ÏLöÉ>Ùgf²OöÉ>Ù'ûÌLöì²ÏÌdÉ>Ù'ûÌLöì²ÏÌdÉ>Ù'ûÌLöì²ÏLöÉ>3²OöÉ>Ùg&ûdìf²OöÉ>Ù'ûÌLöÉ>3Ù'ûdÉ>3²Oöì33Ù'ûdÉ>3²Oöì33Ù'ûdÉ>3²OöÉ>Ùgf²OöÉ>3Ù'ûÌdì²ÏLöÉ>3Ù'ûdÉ>Ùg&ûdì33Ùgf²OöÉ>3f&ûâ6l9rdNNÎÄ×®]+ûÌLöìgöÕÕÕýìg?;xðà~ô£Ù³gË>3f&ûâo¿ýv¸ÐÑÑ1|øð^·"¬ªªýÛÆ~ó3ÐNÚóÃøÅÿåÑ6ÐÂÇìô¥¥ìËÍÍ=vìX¸ðþûïçääüX~µ¯  àèÑ£Éì	èO Ù7fÌÎÎÎpaïÞ½þâóæÍ»çºººî¾ûî9sæøgö­Y³¦²²2;;øðáë×¯÷'Ïì@ö û²8!o¼ñF]]]AAÁèÑ£óßx@~6ýWð û ªºººjkköÙCµ´´|þóÿõ¯ía2ýéOºöÚk=È>ªî¹ç±ÇK655î¹ SUUÕ«¯¾êq@öAT]~ùåDÂãôoË-ÕÕÕdDXEEÅÖ­[gÌ?jÔ¨xÀcdºå[|ï/²¢-//ïÂ/üÃþÐÕÕõúë¯Ï5ëÙgõ°½1bÏ=dDØÒ¿·oóæÍUUU Ý_þòÉ'mÅÅÅo¿ývêððáÃ%%% Ý/~ño¼Ñãìh4iÒ[o½:	8xð`îë_ÿú½÷Þëq@öA´Ýzë­éß¦ýÂ/Ì9ÓÃ¤»øâ_|ñE²¢­­­íüóÏß²eË»ï¾ûúë¯O>Ýt½6ÌozBöAlÞ¼yêÔ©;vìÒ¥K= @/áù¡««Ëãì@ö ûÈ>dì@ö ûÈ>d²ÙìÈ>d²Ùì8=ôÐ%;fÌïÿûÇ;î=zê©ÚÚÚÒÒÒììì²²²k®¹¦©©éOÿp"ÿÅ5kÖTWWäÙpòüüç?ÏÊp×]weÞóÁÌ¼gaaaccãÍ¾¿'ìøhÔÖÖýä'?éìììîî~äGBfÞsÌ1á¦Ûn»­­­íÈ#S§N×ÔÕÕè§QÙÈ>SåèÑ£¡ÿB7.óyyyá¦M6¥®ijj×<83æ7nÜ²²   ¸¸xÞ¼y¡,S7¥$ïßÜÜ__RR4täöíÛ5â+&L0hÐ êêê]»v=÷ÜsáÌÏÏ¿ì²ËÚÛÛSwîç<²àïB?%«¬¬,äZæfÏnÍÎÎ¾úê«xâÞOÙ*-½ðæÏÜìK$¥¥¥éW:4sÉkºõ³ýl²A®¸âä=û?ìø»òòòd*2dýúõwØ½÷¤IREURRrÓM7µ¶¶öµµµ---ûöíûÆ7¾|Ì-Ó¦Mkÿp¸páÂô;_ýõX²dIòðºë®Éo7,**:óÈ>¿ëèèr*¤R_?fûþûï?ôÐC_øÂR_lËÏÏ_·n]_Ùzµ³³3qÆÇÍ¾dq644$Ãá°aÃÒïH$Âåîîîäa[[[8ìêêJ?Uÿçÿ´wïÞäÏçö·wÞygÙ²e^ziò%×¾²/õBðÑ£G3oM-777¤K&Û.'''ýÎ¡8Óßï½ÌSõÙÖ®ºêª³Î:ëµ×^KîÜ¹3ýeÓt#G7½òÊ+©köïß®ÉËËë+ûþå©³ï[+**Ò¿J.ÃÊÊÊãÞ¹ÃþÏ ûÓÚÜ¹sCÍ93ß=æÌgÏyÏÅ¦L²iÓ¦#G$nº)aÂÙÙÙÉ×m÷íÛçÏkjjÒ¿'/üç>löõÙÖZZZÓúuðàÁÛ¶mË¼gGGÇÙgÝëçpÏ8ãßÿþ÷6û¿0õ;ÿ¶oß^TT~Ú²²²Ý»wØìëÿ<²8ÝmÚ´iÆùùù¡fÏþï­e6â×¾öµsÎ9';;»´´´¶¶öüãq¬ÿì[¶lÙèÑ£óòòjjj×Ð¬¯¯Å4±cÇt^æa?çÈ>Ùì@ö ûÈ>d²Ù ûÈ>d²ÙÀGç,ºB«7«IEND®B`


4 Sintomo * n. sintomi * Esito dopo


Test del chi-quadrato	
Esito dopo	Valore	
1	Chi-quadrato di Pearson	.a	
	N di casi validi	1	
Totale	Chi-quadrato di Pearson	.a	
	N di casi validi	1	

a. Non viene calcolata alcuna statistica perché 4 Sintomo e n. sintomi sono costanti.	


Misure simmetriche	
Esito dopo	Valore	
1	Intervallo per intervallo	R di Pearson	.a	
	N di casi validi	1	
Totale	Intervallo per intervallo	R di Pearson	.a	
	N di casi validi	1	

a. Non viene calcolata alcuna statistica perché 4 Sintomo e n. sintomi sono costanti.	

äÞÞÞ³áì33;²ïClµ³!û¯ºêª³$@ÍLöEIÙ÷Ì3Ï,^¼xúôé²ÏÌdCÙ×××·~ýúX,sÑEÕ××ùØq¿MØÑÑQSSSXXXPPPUUuðàÁ3ý<òÈ¥^:cÆE=úè£cÒ×I<rÛ¶m.ÌÎÎN|xòÏô±3æ»ë®»dÉ>3;²/dÓ6ºí¶ÛÞ3ûºººï3gNgggêõ§M6ú°1ßcø:¢ÑhòÿùÏ~Ï8û.¿üòð¬Þ>k~ÊÐÌdÙ4Pâ×^xá¡¡¡ï½7Ï7/5ÇÒúõëÃÍ+WvþcáæºuëRÀµ×^N]sÍ5¯ücW_uò¥&¾Nâ«W¯§^õÕpn®Y³æý>ÜÄff²ÏÌ2'û>ûÙÏãx<¾qãÆÆÆÆþþþq«hL!47ÛÚÚ7ÛÛÛÃÍ¹sç¦>Ä7äF_~Ý»woò¥&¾Nâûöí7KKKßóc'ù[,²ÏÌdeZöMðÑùç¿ûö÷Ì¾ìììpspp0q3$^M½~âCCCÉ½ÔÄ×I<rÌÙð!ïù±²ÏÌdÉ¾ñ·sçÎ;î¸#ñ&^xáf_IIIòwÚÂA¸ÅR¯<þüpjÿþýïØ^jâë$¹gÏÄÍppó.x¿Ïá_üäÉ>3ËìK|ÏlëÖ­CCC!þÂqQQQêÇF"pÜÕÕuìØ±psíÚµáfEEEòÏÕmØ°!õú_ÿú×Ã©/~ñáa¯¼òJâçóF/;ñu|ùáÃ=zÝu×%ÿlßäì33ÙgfçJöMðçW¾ò1÷oÜ¸1µÊÊÊ7«ªªÂÍ&~dtÅÅÅÝÝÝ©O ­­-c~-7qvâë$îIéèñþô§É|¬ì33Ùgf²ï]Ù×ßßûí·ªËÉÉ¹ðÂ¿ûÝï¾ùæ©UÔØØxÑEÇTTT$îÙ·o_MMÍìÙ³óóóÃÁ¡CÎôüñE%ÞxïG[;v,^¼8|øÒ¥KÃqò'ÿdÉ>3³³½YÌLöÉ>33Ùgf&ûÌÌdÉ>3333ffff&ûÌÌÌÌ,ó³¯¡¡¡««ËÿQ333³Ï¾ÿøÇýë_ý5333ffff²OöÉ>Ùgfff&ûdìffff²OöÉ>Ùgfff&ûdì33333Ùgfff&ûdìffff²OöÉ>Ùgfff&ûdìffff]]]ÅÅÅ©÷·¶¶F£ÑòòòææfÙgfffÆÙ÷àeeóÄªªª¶lÙÒßß¿iÓ¦ÊÊÊÔìÛ¶mÛóS»?ìÜµý?ñÄs»ò½òÊ+é×ÍÞ½ÇÍ¾ãÇx<üã_ÚÝñ¿ºÏÿ=c|qëÑW¦|RJ§yßyZãe_vvöéÓ§ÃÁððp4=^äýïÏðe¤úòoy÷g_^^ÞÈÈH"ûBÊ>@öeföõõõÞÞÞX,&ûÙÙW[[»yóæêêjÙÈ¾LË¾ÄÍ¦¦¦X,DâñxKKìd_ÚgßÕÕÕ½çcd ûÒ>ûêëëe û2?û&3ÙÈ>Ù'ûÙ'ûd ûdìdììì²²Oö²OöÉ>@öÉ>ÙÈ>Ù'ûÙ'ûd ûdìdììì²²Oö²OöÉ>@öÉ>ÙÈ>Ù'ûÙ'ûd ûdìdìóÈ>ÙÈ>Ù'ûÙ'ûd ûdìdìì²²Oö²OöÉ>@öÉ>Ù'ûÙ'ûd ûdìdìì²²Oö²OöÉ>@öÉ>ÙÈ>Ù ûd ûdìdìì²²Oö²OöÉ>@öÉ>ÙÈ>Ù'ûÙ'ûdìdìì²²Oö²OöÉ>@öÉ>ÙÈ>Ù'ûÙ'ûd ûdìì²²Oö²OöÉ>@öÉ>ÙÈ¾³4ûZ[[KKK£Ñhyyysssò©¦¦¦K/½477wÉ%Ï=÷ìd_g_UUÕ-[úûû7mÚTYY|ª¤¤äùçë­·ZZZâñ¸ìd_g_h»ãÇ1m·`Á^xáÔ©S»wïÇ©Ù×ØØøÜÔî¿6îõe¤úÂÝnÊ÷òË/§Söegg>:G£ÑäSÍÍÍálVVV¸ÿgIÍ¾¯MíîÜñ/k ÕÕ÷õ¼6å;qâD:e_^^ÞÈÈH"ûBä%úÌg>óØc<yrÛ¶mÿüç½Èx7_ä-++ëëë½½½±X,ùT~~þ©S§ÂA(ÙÜÜÈ¾4Î¾ÚÚÚÍ7444TWW'ª¨¨xöÙgÃ©Çìsûìd_g_SSS,D"ñx¼¥¥åÏ2ëç¹ÿþP3gÎÿÇ²övÍuuuÞ®õõõ²ìdìì²²Oö²OöÉ>@öÉ>@öÉ>ÙÈ>Ù'ûÙ'ûd ûdìdìì²²Oö²OöÉ>@öÉ>@öÉ>ÙÈ>Ù'ûÙ'ûd ûdìdìì²²Oö²OöùÊdìdìì²²Oö²OöÉ>@öÉ>ÙÈ>Ù'ûÙ'ûd ûdìì²²Oö²OöÉ>@öÉ>ÙÈ>Ù'ûÙ'ûd ûdìdì²²Oö²OöÉ>@öÉ>ÙÈ>Ù'ûÙ'ûd ûdìdìì²Oö²OöÉ>@öÉ>ÙÈ>Ù'ûÙ'ûd ûdìdìì²²@öÉ>@öÉ>ÙÈ>Ù'ûÙ'ûd ûdìdßëÖ­;wn4Åb6lèîîìË¨ì;pàÀ9s²Þ½âââÃ«µ¶¶|,//onnN>588¸~ýúóÏ?ÁcNÉ>@öýÛ³¯²²2t^EEE(³¶¶¶+Wjjj>ÀÕªªª¶lÙÒßß¿iÓ¦påäSwÞyç÷¿ÿý7ß|³±±1ìdßf_~~~¼ÎÎÎä×|Ã=àj%%%Ç===ñx<ùÔÂwïÞ=Á¼÷Ýw_ÓÔnã¶û²R-¿ëpÓoâ×Z?ì+,,7ûÂýàjÙÙÙ§OÃÃÃÑh4ùTNNÎwÞ9kÖ¬Å¿øâ©Ù·gÏcS»5÷ú²R]û;6åü÷f_uuuâEÞ¶¶¶ýEÞ¼¼¼DöL>5úô;v ùå^ä¼È;¥/òvttùY³fíÛ·ï¬¬¬¯¯/ôööÆb±q_ÿë­·rrrd û¦úººÖ¯_?oÞ¼ìììg7ÝtÓ¡C>Ø¥jkk7oÞ<00ÐÐÐP]]|êæoþå/ï©§Z¶lìd_¿]sSSS,D"ñx¼¥¥åÏ2ëçùúë¯¯Y³&??ÿ²Ë.é¥d û¦"û/æ»ëYÖÕÕy»f@öe~öÕ××Ë>@öeÔ¼þq6@ö¥Ùw¦oõÍ9sáÂ?ýéOO:%ûÙ±Ù7ºÉ¼D+ûÙw¶gß#G.¹äU«Vµ··´µµ-_¾ü+®øÛßþö³ý,dßüùóe ûÒ>û®¼òÊ1ÿ8ÛáÃÃ=7ÜpÃ±cÇÂÁlCö²/-³oÆ¡íº»»Gï9zôhâgûd û2$û>ùÉO¶»îºë:::N<¹ÿþpî¹øâ·oßJKKe ûÒ>ûzè¡éÓ§ù5pÏ<8¾í¶Ûd û2áûöÙë¯¿þcûX$)))ùêW¿ÚÚÚî9sfmmm¿ìd·kìóvÍ²Þ®Yö²ÏÛ5Ë>@öy»fÙÈ>o×,ûÙçíep®f·k8'²ÏÛ5+ÙçíÎìúé§«ªª#H¸¹téÒ|Pö²/£²ïw¿ûÝ´iÓFªoôï¾ûnÙÈ¾ÌÉ¾EÈÛ¶mÛhöÝï½á`Á²ÙÙÙ!òoÑÈ¾7Þx#äääÈ>@öeNöêSwÿý÷'²¯···®®.,Y²Dö²/s²ïá÷ûzè!ÙÈ¾úMÞ=ö|ík_?þ3æÍWSS³sçN¿ÉÈ¾Ê¾ÑéÝ±cÇªªªV­Z%ûÙÉÙ×ÕÕîÉÍÍìKûì[±bEÖ»øâe ûÒ>ûÎ|H¤¬¬ììËäy?É>@öý³¯··÷Æo,,,<ï¼óÆ|ÛOö²/s²¯¶¶öL¯öÊ>@öeNöÍ5+Þ÷¾÷½¾¾¾/òdföÍ93dßë¯¿îgû29û®ºêª­­­3fÌ¸úê«<844$û2ùJììKÿì;K&ûÙ÷oÏ¾ÎÎÎuëÖÍ;7Æb±6twwË>@öeTö8p`Î9c^Þ-..>|ø°ìd_æd_eeeè¼öööÁÁÁ¶¶¶+Wjjjd û2'ûòóóCäuvv&¿æî)((ìËì+,,7ûÂý²ÕÕÕyÛÚÚ¼È±Ù×ÑÑQTT4æW:fÍµoß>ÙÈ¾z®®®õë×Ï7/;;»¤¤ä¦nú¢Wö²ÏÛ5Ë>@öýÛ½÷WÔÔ|g¸¹jÕªöövÙÈ¾LÈ¾¼¼¼¬¬¬+V$ßn;/¸à_~Yö²/í³oíÚµ!ïâñxKKKòýO?ýôÜ¹sÃ©[o½Uö²/í³oÞ¼y¡íxâÔS?üp8µ`ÁÙÈ¾´Ï¾H$ÚîÄ©§þþ÷¿S¹¹¹²iãðáÃ©§9NÍ=[ö²/í³/ñOqüà?H=õÃþ0Z¶lìd_ÚgßÖ­[CÛM>=_GGÇÐÐÐÉ'÷îÝÇw;Ã©_ÿú×²ð¾·ÞzkÖVWWwúôiÙÈ¾LÈ¾°;vÔÖÖÎ??'''Æb±5kÖûë½²igÏd ûdìdìì²²Oö²Oö²OöM°ÖÖÖÒÒÒh4Z^^ÞÜÜúíÛ·geeÉ>@ö¥wöUUUmÙ²¥¿¿Ó¦McÎ.Z´Hö²/í³¯¤¤äøñãá §§'9û£ýè'?ùÉ²ï7¿ùÍS»õ[ÿìËHµìWÿçÉ)ßÁÓ)û²³³ÿªÛððp4M>uèÐ¡òòò'O)û^zé¥S»ÿöì1_Ö@ªëîï=1åJ§ìËËËId_HÀäS×_ýã?þÎö"/àEÞt·¬¬¬¯¯/ôööÆb±w=×wOö²/³¯¶¶vóæÍÕÕÕã?ißíd_ºg_SSS,D"ñx¼¥¥eÜÎìËÌ·k®««óvÍìËüì«¯¯ìËüìÌd ûdìdìì²²Oö²Oö²OöÉ>@öÉ>ÙÈ>Ù'ûÙ'ûd ûdìdìì²²Oö²Oö²OöÉ>@öÉ>ÙÈ>Ù'ûÙ'ûd ûdìdìì²²ÏW6 ûd ûdìdìì²²Oö²OöÉ>@öÉ>ÙÈ>Ù'ûÙ'ûdìdìì²²Oö²OöÉ>@öÉ>ÙÈ>Ù'ûÙ'ûd ûdìì²²Oö²OöÉ>@öÉ>ÙÈ>Ù'ûÙ'ûd ûdìdì²²Oö²OöÉ>@öÉ>ÙÈ>Ù'ûÙ'ûd ûdìdìì²Oö²OöÉ>@öÉ>ÙÈ>Ù'ûÙ'ûd ûdìdßG·ÖÖÖÒÒÒh4Z^^ÞÜÜ|ª¥¥eÉ%¹¹¹K.Ýµkìd_g_UUÕ-[úûû7mÚTYY|êK.Ù¾ûààà/~ñEÉ>@ö¥qö?~<ôôôÄãñq300PXXwÝu×#S»u÷üo_Ö@ªÏýÇÁG¦|éÙÙÙ§OÃÃÃÑhtÜÇ<õÔS7ÝtSjö8p`hj·©å¸/k ÕÿïÐ/äS:e_^^ÞÈÈH"ûB¦> ¯¯ïË_þòo¼áE^À¼iü"oYYY»pÐÛÛÅÆ=zôhmmí#Gü&/ ûÒ;ûBÕmÞ¼y`` ¡¡¡ºº:ùÔO<±|ùòoàÈ¾´Ï¾¦¦¦X,DâñxKKË?eÖ;Ï3Ü4ÙÈ¾L»æºº:o×È¾ÌÏ¾úúzÙÈ¾ÌÏ¾ÉLö²OöÉ>@öÉ>ÙÈ>Ù'ûÙ'ûd ûd ûdìdìì²²Oö²OöÉ>@öÉ>ÙÈ>Ù'ûÙ'ûd ûd ûdìdìì²²Oö²OöÉ>@öÉ>ÙÈ>Ù'ûÙ'û|e²Oö²OöÉ>@öÉ>ÙÈ>Ù'ûÙ'ûd ûdìdìì²²@öÉ>@öÉ>ÙÈ>Ù'ûÙ'ûd ûdìdìì²²Oö²OöÈ>ÙÈ>Ù'ûÙ'ûd ûdìdìì²²Oö²OöÉ>@öÉ>Ù'ûÙ'ûd ûdìdìì²²Oö²OöÉ>@öÉ>ÙÈ>Ù ûd ûdìdìì²²Oö²OöÉ>@ötkmm---F£åååÍÍÍ<%ûÙfÙWUUµeËþþþM6UVVNòìd_e_IIÉñãÇÃAOOO<ä©°PË/_5µûô¹ÁwïcÑÿ±jÊr(²/;;ûôéÓá`xx8Nò¥ÙwûòòòFFFm:o§ÌÌÌÌ,Í²¯¬¬¬¯¯/ôööÆb±I2333³4Ë¾ÚÚÚÍ7444TWWOò¥Yö555Åb±H$Ç[ZZþù,³²ÎtÊÌÌÌÌÒ2ûÆ]]]ÿUffffõõõþWe~öì33333Ùgf65¾Ó¦Mó913ÙgfáûÃþð­oËçÁÌdYoùòåû÷ï÷y03ÙgfÉñÅW¬Xáó`f²ÏÌ,ÃwÛm·ýêW¿òy03Ùgfá»ðÂ=êó`f²ÏÌ,÷¿üeÉ%>f&ûÌÌ2|?ÿùÏ7nÜèó`f²ÏÌ,Ã÷íoû®»îòy03Ùgfá»â+vîÜéó`f²ÏÌ,Ã7wîÜ®®.3ff¾3fø<ì33333Ùgffff²ÏÌÌÌÌdÉ>3333ffff²ÏÌÌÌÌdÉ>3333ffff&ûÌÌÌÌLöì33333Ùgffff²ÏÌÌÌLöì33333Ùgffff²ÏÌÌÌÌdÉ>3³¼ÎÎÎ¬¬3þ¥÷è£®^½º¨¨(ãßØ»wïÿÿ»òÌÔÔÔ´bÅp3ffÍ®¾úê	ÒíîÉJY~~~ûûÍ¾É?ÒÌLöÈ»ûî»GcnÜS·ß~û#GN<ÙÖÖ¶lÙ²pOUUÕûþ[Uöì33ûHÖÝÝ]XXxÁLd999áÔ®]»FïÙ»wo¸göìÙ©18~þùçW¯^7kÖ¬ÚÚÚ¾¾¾ÑSc³£££¦¦&<ÐÓÛ·o_¼xñ3V¬Xñê«¯þþ÷¿_¸pannîW^ÙÙÙ9úà	®cf&ûÌÌÞÒª±±qì«¬¬§"È7ÜðÐCý»2%ûB¥%ÞÚµkÇÍ¾®®®¢¢¢ä;çÌ3s¦M6zöÓþt¢A»öÚkø:ff²ÏÌÎõmÝº5Ò5kÞðå×îîîË.»l´¨o¹åÃO«W¯>pàÀ±cÇ¾óï$>$õaë×¯7W®ùUTTëÖ­K~ðÍ7ßüÆoÜï½7Þxc¸øqÃÉÌLöÙ9½×^íãÿxQQQOOÏÛïõSwÃÃÃ÷Ýwß¾ô¥Ño¶åææ>ýôÓgÊ¾Ñ×XûúúÂÍóÎ;oÜì7o^¸ÙÖÖ¸ÙÞÞnÎ;7ùÁ]]]áxpp0qóÈ#áæÀÀ@ò¥&¾ì3³sz7ÜpCh£î¹gÜ ;ÓN8ÑØØø/|!ñë²oôàÔ³£WËÎÎ7CÒ%n&Ú.&?8gòÍS§N¥^jâëÉ>3;·ÿ;ÃRYZZîß³gÏè=¯¿þz¸'''çLÙú¶¤¤$ù»tá ÜÅbã>x_ÇÌLöìTömØ°!Ü¿téÒ]»v<y²««ë[n	÷,^¼øýf_$I¼nìØ±psíÚµáfEEEòÏä?îýfßÄ×13ffo)ª×ÓÓ3ú/£;ï¼ó~ûÛß¾ßìK¼àèþ<x0ñ¯®¸¸¸»»ûýfßÄ×13ffÊ¾°|óßüÄ'>DV¯^ýäOû±g_ccãE]SQQ¸gß¾555³gÏÎÏÏLç¥Þà:ff²ÏÌÌÌÌdì33333Ùgffff²ÏÌÌÌÌdÉ>3333ffff&ûÌÌÌÌLöì3333ffff&ûÌÌÌÌLöì33333Ùgffff²ÏÌÌÌÌþý?/;©ÂyIEND®B`


CROSSTABS
  /TABLES=@1Sintomo @2Sintomo @3Sintomo @4Sintomo BY n.sintomi BY Esitodopo
  /SHOWDIM=2
  /FORMAT=AVALUE TABLES
  /STATISTICS=CHISQ CORR
  /CELLS=COUNT
  /COUNT ROUND CELL.


Tabelle di contingenza


Note	
Output creato	30-NOV-2020 12:44:54	
Commenti		
Input	Dataset attivo	Dataset1	
	Filtro	<nessuno>	
	Peso	<nessuno>	
	Suddividi file	<nessuno>	
	N di righe nel file di dati di lavoro	54	
Gestione valori mancanti	Definizione di mancante	I valori mancanti definiti dall'utente vengono trattati come mancanti.	
	Casi utilizzati	Le statistiche per ciascuna tabella sono basate su tutti i casi con dati validi nell'intervallo o negli intervalli specificati per tutte le variabili in ciascuna tabella.	
Sintassi	CROSSTABS
  /TABLES=@1Sintomo @2Sintomo @3Sintomo @4Sintomo BY n.sintomi BY Esitodopo
  /SHOWDIM=2
  /FORMAT=AVALUE TABLES
  /STATISTICS=CHISQ CORR
  /CELLS=COUNT
  /COUNT ROUND CELL.	
Risorse	Tempo processore	00:00:00,00	
	Tempo trascorso	00:00:00,00	
	Dimensioni richieste	3	
	Celle disponibili	449353	


Riepilogo elaborazione casi	
	Casi	
	Valido	Mancante	Totale	
	N	Percentuale	N	Percentuale	N	
1 Sintomo * n. sintomi * Esito dopo	54	100,0%	0	0,0%	54	
2 Sintomo * n. sintomi * Esito dopo	22	40,7%	32	59,3%	54	
3 Sintomo * n. sintomi * Esito dopo	11	20,4%	43	79,6%	54	
4 Sintomo * n. sintomi * Esito dopo	1	1,9%	53	98,1%	54	

Riepilogo elaborazione casi	
	Casi	
	Totale	
	Percentuale	
1 Sintomo * n. sintomi * Esito dopo	100,0%	
2 Sintomo * n. sintomi * Esito dopo	100,0%	
3 Sintomo * n. sintomi * Esito dopo	100,0%	
4 Sintomo * n. sintomi * Esito dopo	100,0%	


1 Sintomo * n. sintomi * Esito dopo


Tabella di contingenza	
Esito dopo:   Totale  	
Conteggio  	
	n. sintomi	Totale	
	0	1	2	3	4		
1 Sintomo	0	21	0	0	0	0	21	
	1	0	4	8	10	1	23	
	3	0	0	1	0	0	1	
	4	0	3	1	0	0	4	
	7	0	3	0	0	0	3	
	9	0	0	1	0	0	1	
	11	0	1	0	0	0	1	
Totale	21	11	11	10	1	54	


Test del chi-quadrato	
Esito dopo	Valore	df	Significatività asintotica (bilaterale)	
1	Chi-quadrato di Pearson	26,341b	20	,155	
	Rapporto di verosimiglianza	26,369	20	,154	
	Associazione lineare per lineare	,523	1	,470	
	N di casi validi	19			
2	Chi-quadrato di Pearson	62,500c	12	,000	
	Rapporto di verosimiglianza	65,665	12	,000	
	Associazione lineare per lineare	,817	1	,366	
	N di casi validi	35			
Totale	Chi-quadrato di Pearson	84,628a	24	,000	
	Rapporto di verosimiglianza	93,063	24	,000	
	Associazione lineare per lineare	1,565	1	,211	
	N di casi validi	54			

a. 33 celle (94,3%) hanno un conteggio previsto inferiore a 5. Il conteggio previsto minimo è ,02.	
b. 30 celle (100,0%) hanno un conteggio previsto inferiore a 5. Il conteggio previsto minimo è ,05.	
c. 18 celle (90,0%) hanno un conteggio previsto inferiore a 5. Il conteggio previsto minimo è ,09.	


Misure simmetriche	
Esito dopo	Valore	Errore standard asintoticoa	
1	Intervallo per intervallo	R di Pearson	,170	,150	
	Ordinale per ordinale	Correlazione di Spearman	,519	,218	
	N di casi validi	19		
2	Intervallo per intervallo	R di Pearson	,155	,076	
	Ordinale per ordinale	Correlazione di Spearman	,780	,101	
	N di casi validi	35		
Totale	Intervallo per intervallo	R di Pearson	,172	,079	
	Ordinale per ordinale	Correlazione di Spearman	,689	,102	
	N di casi validi	54		

Misure simmetriche	
Esito dopo	T approssimatob	Significatività approssimata	
1	Intervallo per intervallo	R di Pearson	,713	,485c	
	Ordinale per ordinale	Correlazione di Spearman	2,506	,023c	
	N di casi validi			
2	Intervallo per intervallo	R di Pearson	,902	,374c	
	Ordinale per ordinale	Correlazione di Spearman	7,154	,000c	
	N di casi validi			
Totale	Intervallo per intervallo	R di Pearson	1,258	,214c	
	Ordinale per ordinale	Correlazione di Spearman	6,861	,000c	
	N di casi validi			

a. Non viene assunta l'ipotesi nulla.	
b. Viene utilizzato l'errore standard asintotico presumendo l'ipotesi nulla.	
c. Basato sull'approssimazione normale.	


2 Sintomo * n. sintomi * Esito dopo


Tabella di contingenza	
Esito dopo:   Totale  	
Conteggio  	
	n. sintomi	Totale	
	2	3	4		
2 Sintomo	3	1	0	0	1	
	4	2	0	0	2	
	7	4	0	0	4	
	8	1	2	0	3	
	9	3	7	1	11	
	10	0	1	0	1	
Totale	11	10	1	22	


Test del chi-quadrato	
Esito dopo	Valore	df	Significatività asintotica (bilaterale)	
1	Chi-quadrato di Pearson	4,950b	8	,763	
	Rapporto di verosimiglianza	6,161	8	,629	
	Associazione lineare per lineare	2,116	1	,146	
	N di casi validi	11			
2	Chi-quadrato di Pearson	6,799c	4	,147	
	Rapporto di verosimiglianza	7,484	4	,112	
	Associazione lineare per lineare	4,064	1	,044	
	N di casi validi	11			
Totale	Chi-quadrato di Pearson	11,236a	10	,339	
	Rapporto di verosimiglianza	14,462	10	,153	
	Associazione lineare per lineare	6,503	1	,011	
	N di casi validi	22			

a. 16 celle (88,9%) hanno un conteggio previsto inferiore a 5. Il conteggio previsto minimo è ,05.	
b. 15 celle (100,0%) hanno un conteggio previsto inferiore a 5. Il conteggio previsto minimo è ,09.	
c. 10 celle (100,0%) hanno un conteggio previsto inferiore a 5. Il conteggio previsto minimo è ,27.	


Misure simmetriche	
Esito dopo	Valore	Errore standard asintoticoa	
1	Intervallo per intervallo	R di Pearson	,460	,116	
	Ordinale per ordinale	Correlazione di Spearman	,609	,147	
	N di casi validi	11		
2	Intervallo per intervallo	R di Pearson	,637	,196	
	Ordinale per ordinale	Correlazione di Spearman	,529	,260	
	N di casi validi	11		
Totale	Intervallo per intervallo	R di Pearson	,556	,092	
	Ordinale per ordinale	Correlazione di Spearman	,631	,124	
	N di casi validi	22		

Misure simmetriche	
Esito dopo	T approssimatob	Significatività approssimata	
1	Intervallo per intervallo	R di Pearson	1,554	,155c	
	Ordinale per ordinale	Correlazione di Spearman	2,301	,047c	
	N di casi validi			
2	Intervallo per intervallo	R di Pearson	2,482	,035c	
	Ordinale per ordinale	Correlazione di Spearman	1,872	,094c	
	N di casi validi			
Totale	Intervallo per intervallo	R di Pearson	2,995	,007c	
	Ordinale per ordinale	Correlazione di Spearman	3,640	,002c	
	N di casi validi			

a. Non viene assunta l'ipotesi nulla.	
b. Viene utilizzato l'errore standard asintotico presumendo l'ipotesi nulla.	
c. Basato sull'approssimazione normale.	


3 Sintomo * n. sintomi * Esito dopo


Tabella di contingenza	
Esito dopo:   Totale  	
Conteggio  	
	n. sintomi	Totale	
	3	4		
3 Sintomo	2	0	1	1	
	6	2	0	2	
	7	8	0	8	
Totale	10	1	11	


Test del chi-quadrato	
Esito dopo	Valore	df	Significatività asintotica (bilaterale)	Sign. esatta (bilaterale)	
1	Chi-quadrato di Pearson	3,000b	1	,083		
	Correzione di continuitàc	,188	1	,665		
	Rapporto di verosimiglianza	3,819	1	,051		
	Test esatto di Fisher				,333	
	Associazione lineare per lineare	2,000	1	,157		
	N di casi validi	3				
2	Chi-quadrato di Pearson	.d				
	N di casi validi	8				
Totale	Chi-quadrato di Pearson	11,000a	2	,004		
	Rapporto di verosimiglianza	6,702	2	,035		
	Associazione lineare per lineare	9,290	1	,002		
	N di casi validi	11				

Test del chi-quadrato	
Esito dopo	Sign. esatta (unilaterale)	
1	Chi-quadrato di Pearson		
	Correzione di continuitàc		
	Rapporto di verosimiglianza		
	Test esatto di Fisher	,333	
	Associazione lineare per lineare		
	N di casi validi		
2	Chi-quadrato di Pearson		
	N di casi validi		
Totale	Chi-quadrato di Pearson		
	Rapporto di verosimiglianza		
	Associazione lineare per lineare		
	N di casi validi		

a. 5 celle (83,3%) hanno un conteggio previsto inferiore a 5. Il conteggio previsto minimo è ,09.	
b. 4 celle (100,0%) hanno un conteggio previsto inferiore a 5. Il conteggio previsto minimo è ,33.	
c. Calcolato solo per una tabella 2x2	
d. Non viene calcolata alcuna statistica perché n. sintomi è una costante.	


Misure simmetriche	
Esito dopo	Valore	Errore standard asintoticoa	
1	Intervallo per intervallo	R di Pearson	-1,000	,000	
	Ordinale per ordinale	Correlazione di Spearman	-1,000	,000c	
	N di casi validi	3		
2	Intervallo per intervallo	R di Pearson	.d		
	N di casi validi	8		
Totale	Intervallo per intervallo	R di Pearson	-,964	,037	
	Ordinale per ordinale	Correlazione di Spearman	-,638	,234	
	N di casi validi	11		

Misure simmetriche	
Esito dopo	T approssimatob	Significatività approssimata	
1	Intervallo per intervallo	R di Pearson	-94906265,624	,000c	
	Ordinale per ordinale	Correlazione di Spearman			
	N di casi validi			
2	Intervallo per intervallo	R di Pearson			
	N di casi validi			
Totale	Intervallo per intervallo	R di Pearson	-10,854	,000c	
	Ordinale per ordinale	Correlazione di Spearman	-2,487	,035c	
	N di casi validi			

a. Non viene assunta l'ipotesi nulla.	
b. Viene utilizzato l'errore standard asintotico presumendo l'ipotesi nulla.	
c. Basato sull'approssimazione normale.	
d. Non viene calcolata alcuna statistica perché n. sintomi è una costante.	


4 Sintomo * n. sintomi * Esito dopo


Tabella di contingenza	
Esito dopo:   Totale  	
Conteggio  	
	n. sintomi	Totale	
	4		
4 Sintomo	7	1	1	
Totale	1	1	


Test del chi-quadrato	
Esito dopo	Valore	
1	Chi-quadrato di Pearson	.a	
	N di casi validi	1	
Totale	Chi-quadrato di Pearson	.a	
	N di casi validi	1	

a. Non viene calcolata alcuna statistica perché 4 Sintomo e n. sintomi sono costanti.	


Misure simmetriche	
Esito dopo	Valore	
1	Intervallo per intervallo	R di Pearson	.a	
	N di casi validi	1	
Totale	Intervallo per intervallo	R di Pearson	.a	
	N di casi validi	1	

a. Non viene calcolata alcuna statistica perché 4 Sintomo e n. sintomi sono costanti.	

CROSSTABS
  /TABLES=@1Sintomo @2Sintomo @3Sintomo @4Sintomo BY n.sintomi BY Esitodopo
  /FORMAT=AVALUE TABLES
  /STATISTICS=CHISQ CC PHI LAMBDA UC ETA CORR GAMMA D BTAU CTAU KAPPA RISK MCNEMAR CMH(1)
  /CELLS=COUNT
  /COUNT ROUND CELL.


Tabelle di contingenza


Note	
Output creato	30-NOV-2020 12:46:22	
Commenti		
Input	Dataset attivo	Dataset1	
	Filtro	<nessuno>	
	Peso	<nessuno>	
	Suddividi file	<nessuno>	
	N di righe nel file di dati di lavoro	54	
Gestione valori mancanti	Definizione di mancante	I valori mancanti definiti dall'utente vengono trattati come mancanti.	
	Casi utilizzati	Le statistiche per ciascuna tabella sono basate su tutti i casi con dati validi nell'intervallo o negli intervalli specificati per tutte le variabili in ciascuna tabella.	
Sintassi	CROSSTABS
  /TABLES=@1Sintomo @2Sintomo @3Sintomo @4Sintomo BY n.sintomi BY Esitodopo
  /FORMAT=AVALUE TABLES
  /STATISTICS=CHISQ CC PHI LAMBDA UC ETA CORR GAMMA D BTAU CTAU KAPPA RISK MCNEMAR CMH(1)
  /CELLS=COUNT
  /COUNT ROUND CELL.	
Risorse	Tempo processore	00:00:00,02	
	Tempo trascorso	00:00:00,02	
	Dimensioni richieste	3	
	Celle disponibili	449353	


Avvisi	
La tabella di test di omogeneità del rapporto odd e la tabella di stima del rapporto odd comune di Mantel-Haenszel non vengono calcolate per 1 Sintomo * n. sintomi * Esito dopo, perché (1) la variabile di gruppo non ha esattamente due valori non mancanti distinti o/e (2) la variabile di risposta non ha esattamente due valori non mancanti distinti.	
La tabella di test di omogeneità del rapporto odd e la tabella di stima del rapporto odd comune di Mantel-Haenszel non vengono calcolate per 2 Sintomo * n. sintomi * Esito dopo, perché (1) la variabile di gruppo non ha esattamente due valori non mancanti distinti o/e (2) la variabile di risposta non ha esattamente due valori non mancanti distinti.	
La tabella di test di omogeneità del rapporto odd e la tabella di stima del rapporto odd comune di Mantel-Haenszel non vengono calcolate per 3 Sintomo * n. sintomi * Esito dopo, perché (1) la variabile di gruppo non ha esattamente due valori non mancanti distinti o/e (2) la variabile di risposta non ha esattamente due valori non mancanti distinti.	


Riepilogo elaborazione casi	
	Casi	
	Valido	Mancante	Totale	
	N	Percentuale	N	Percentuale	N	
1 Sintomo * n. sintomi * Esito dopo	54	100,0%	0	0,0%	54	
2 Sintomo * n. sintomi * Esito dopo	22	40,7%	32	59,3%	54	
3 Sintomo * n. sintomi * Esito dopo	11	20,4%	43	79,6%	54	
4 Sintomo * n. sintomi * Esito dopo	1	1,9%	53	98,1%	54	

Riepilogo elaborazione casi	
	Casi	
	Totale	
	Percentuale	
1 Sintomo * n. sintomi * Esito dopo	100,0%	
2 Sintomo * n. sintomi * Esito dopo	100,0%	
3 Sintomo * n. sintomi * Esito dopo	100,0%	
4 Sintomo * n. sintomi * Esito dopo	100,0%	


1 Sintomo * n. sintomi * Esito dopo


Tabella di contingenza	
Conteggio  	
Esito dopo	n. sintomi	Totale	
	0	1	2	3	4		
1	1 Sintomo	0	4	0	0	0	0	4	
		1	0	3	5	2	1	11	
		3	0	0	1	0	0	1	
		4	0	0	1	0	0	1	
		7	0	1	0	0	0	1	
		9	0	0	1	0	0	1	
	Totale	4	4	8	2	1	19	
2	1 Sintomo	0	17	0	0	0		17	
		1	0	1	3	8		12	
		4	0	3	0	0		3	
		7	0	2	0	0		2	
		11	0	1	0	0		1	
	Totale	17	7	3	8		35	
Totale	1 Sintomo	0	21	0	0	0	0	21	
		1	0	4	8	10	1	23	
		3	0	0	1	0	0	1	
		4	0	3	1	0	0	4	
		7	0	3	0	0	0	3	
		9	0	0	1	0	0	1	
		11	0	1	0	0	0	1	
	Totale	21	11	11	10	1	54	


Test del chi-quadrato	
Esito dopo	Valore	df	Significatività asintotica (bilaterale)	
1	Chi-quadrato di Pearson	26,341c	20	,155	
	Rapporto di verosimiglianza	26,369	20	,154	
	Associazione lineare per lineare	,523	1	,470	
	Test McNemar-Bowker	.	.	.b	
	N di casi validi	19			
2	Chi-quadrato di Pearson	62,500d	12	,000	
	Rapporto di verosimiglianza	65,665	12	,000	
	Associazione lineare per lineare	,817	1	,366	
	Test McNemar-Bowker	.	.	.b	
	N di casi validi	35			
Totale	Chi-quadrato di Pearson	84,628a	24	,000	
	Rapporto di verosimiglianza	93,063	24	,000	
	Associazione lineare per lineare	1,565	1	,211	
	Test McNemar-Bowker	.	.	.b	
	N di casi validi	54			

a. 33 celle (94,3%) hanno un conteggio previsto inferiore a 5. Il conteggio previsto minimo è ,02.	
b. Calcolato solo per una tabella PxP, dove P deve essere maggiore di 1.	
c. 30 celle (100,0%) hanno un conteggio previsto inferiore a 5. Il conteggio previsto minimo è ,05.	
d. 18 celle (90,0%) hanno un conteggio previsto inferiore a 5. Il conteggio previsto minimo è ,09.	


Misure direzionali	
Esito dopo	Valore	Errore standard asintoticoa	T approssimatob	
1	Nominale per nominale	Lambda	Simmetrico	,474	,134	2,522	
			1 Sintomo dipendente	,500	,177	2,251	
			n. sintomi dipendente	,455	,150	2,605	
		Tau di Goodman e Kruskal	1 Sintomo dipendente	,482	,064		
			n. sintomi dipendente	,455	,026		
		Coefficiente di incertezza	Simmetrico	,519	,072	5,228	
			1 Sintomo dipendente	,549	,084	5,228	
			n. sintomi dipendente	,491	,095	5,228	
	Ordinale per ordinale	D di Somers	Simmetrico	,425	,199	2,061	
			1 Sintomo dipendente	,392	,189	2,061	
			n. sintomi dipendente	,464	,219	2,061	
	Nominale per intervallo	Eta	1 Sintomo dipendente	,460			
			n. sintomi dipendente	,779			
2	Nominale per nominale	Lambda	Simmetrico	,778	,071	5,330	
			1 Sintomo dipendente	,778	,098	4,830	
			n. sintomi dipendente	,778	,098	4,830	
		Tau di Goodman e Kruskal	1 Sintomo dipendente	,781	,031		
			n. sintomi dipendente	,749	,062		
		Coefficiente di incertezza	Simmetrico	,777	,051	12,428	
			1 Sintomo dipendente	,786	,061	12,428	
			n. sintomi dipendente	,769	,065	12,428	
	Ordinale per ordinale	D di Somers	Simmetrico	,603	,113	8,699	
			1 Sintomo dipendente	,590	,109	8,699	
			n. sintomi dipendente	,617	,119	8,699	
	Nominale per intervallo	Eta	1 Sintomo dipendente	,841			
			n. sintomi dipendente	,951			
Totale	Nominale per nominale	Lambda	Simmetrico	,625	,053	12,421	
			1 Sintomo dipendente	,677	,084	5,862	
			n. sintomi dipendente	,576	,086	5,414	
		Tau di Goodman e Kruskal	1 Sintomo dipendente	,642	,042		
			n. sintomi dipendente	,579	,034		
		Coefficiente di incertezza	Simmetrico	,637	,040	15,203	
			1 Sintomo dipendente	,660	,048	15,203	
			n. sintomi dipendente	,615	,052	15,203	
	Ordinale per ordinale	D di Somers	Simmetrico	,526	,103	6,132	
			1 Sintomo dipendente	,500	,098	6,132	
			n. sintomi dipendente	,556	,111	6,132	
	Nominale per intervallo	Eta	1 Sintomo dipendente	,676			
			n. sintomi dipendente	,892			

Misure direzionali	
Esito dopo	Significatività approssimata	
1	Nominale per nominale	Lambda	Simmetrico	,012	
			1 Sintomo dipendente	,024	
			n. sintomi dipendente	,009	
		Tau di Goodman e Kruskal	1 Sintomo dipendente	,002c	
			n. sintomi dipendente	,036c	
		Coefficiente di incertezza	Simmetrico	,154d	
			1 Sintomo dipendente	,154d	
			n. sintomi dipendente	,154d	
	Ordinale per ordinale	D di Somers	Simmetrico	,039	
			1 Sintomo dipendente	,039	
			n. sintomi dipendente	,039	
	Nominale per intervallo	Eta	1 Sintomo dipendente		
			n. sintomi dipendente		
2	Nominale per nominale	Lambda	Simmetrico	,000	
			1 Sintomo dipendente	,000	
			n. sintomi dipendente	,000	
		Tau di Goodman e Kruskal	1 Sintomo dipendente	,000c	
			n. sintomi dipendente	,000c	
		Coefficiente di incertezza	Simmetrico	,000d	
			1 Sintomo dipendente	,000d	
			n. sintomi dipendente	,000d	
	Ordinale per ordinale	D di Somers	Simmetrico	,000	
			1 Sintomo dipendente	,000	
			n. sintomi dipendente	,000	
	Nominale per intervallo	Eta	1 Sintomo dipendente		
			n. sintomi dipendente		
Totale	Nominale per nominale	Lambda	Simmetrico	,000	
			1 Sintomo dipendente	,000	
			n. sintomi dipendente	,000	
		Tau di Goodman e Kruskal	1 Sintomo dipendente	,000c	
			n. sintomi dipendente	,000c	
		Coefficiente di incertezza	Simmetrico	,000d	
			1 Sintomo dipendente	,000d	
			n. sintomi dipendente	,000d	
	Ordinale per ordinale	D di Somers	Simmetrico	,000	
			1 Sintomo dipendente	,000	
			n. sintomi dipendente	,000	
	Nominale per intervallo	Eta	1 Sintomo dipendente		
			n. sintomi dipendente		

a. Non viene assunta l'ipotesi nulla.	
b. Viene utilizzato l'errore standard asintotico presumendo l'ipotesi nulla.	
c. Basato sull'approssimazione chi-quadrato	
d. Probabilità chi-quadrato del rapporto di verosimiglianza.	


Misure simmetriche	
Esito dopo	Valore	Errore standard asintoticoa	
1	Nominale per nominale	Phi	1,177		
		V di Cramer	,589		
		Coefficiente di contingenza	,762		
	Ordinale per ordinale	Tau-b di Kendall	,426	,199	
		Tau-c di Kendall	,353	,171	
		Gamma	Ordine zero	,573	,242	
		Correlazione di Spearman	,519	,218	
	Intervallo per intervallo	R di Pearson	,170	,150	
	Misura di accordo	Kappa	,235	,102	
	N di casi validi	19		
2	Nominale per nominale	Phi	1,336		
		V di Cramer	,772		
		Coefficiente di contingenza	,801		
	Ordinale per ordinale	Tau-b di Kendall	,603	,113	
		Tau-c di Kendall	,522	,060	
		Gamma	Ordine zero	,645	,113	
		Correlazione di Spearman	,780	,101	
	Intervallo per intervallo	R di Pearson	,155	,076	
	Misura di accordo	Kappa	,302	,064	
	N di casi validi	35		
Totale	Nominale per nominale	Phi	1,252		
		V di Cramer	,626		
		Coefficiente di contingenza	,781		
	Ordinale per ordinale	Tau-b di Kendall	,527	,103	
		Tau-c di Kendall	,457	,075	
		Gamma	Ordine zero	,600	,110	
			Parziale di ordine 1	,631		
		Correlazione di Spearman	,689	,102	
	Intervallo per intervallo	R di Pearson	,172	,079	
	Misura di accordo	Kappa	,291	,059	
	N di casi validi	54		

Misure simmetriche	
Esito dopo	T approssimatob	Significatività approssimata	
1	Nominale per nominale	Phi		,155	
		V di Cramer		,155	
		Coefficiente di contingenza		,155	
	Ordinale per ordinale	Tau-b di Kendall	2,061	,039	
		Tau-c di Kendall	2,061	,039	
		Gamma	Ordine zero	2,061	,039	
		Correlazione di Spearman	2,506	,023c	
	Intervallo per intervallo	R di Pearson	,713	,485c	
	Misura di accordo	Kappa	2,834	,005	
	N di casi validi			
2	Nominale per nominale	Phi		,000	
		V di Cramer		,000	
		Coefficiente di contingenza		,000	
	Ordinale per ordinale	Tau-b di Kendall	8,699	,000	
		Tau-c di Kendall	8,699	,000	
		Gamma	Ordine zero	8,699	,000	
		Correlazione di Spearman	7,154	,000c	
	Intervallo per intervallo	R di Pearson	,902	,374c	
	Misura di accordo	Kappa	3,432	,001	
	N di casi validi			
Totale	Nominale per nominale	Phi		,000	
		V di Cramer		,000	
		Coefficiente di contingenza		,000	
	Ordinale per ordinale	Tau-b di Kendall	6,132	,000	
		Tau-c di Kendall	6,132	,000	
		Gamma	Ordine zero	6,132	,000	
			Parziale di ordine 1			
		Correlazione di Spearman	6,861	,000c	
	Intervallo per intervallo	R di Pearson	1,258	,214c	
	Misura di accordo	Kappa	4,510	,000	
	N di casi validi			

a. Non viene assunta l'ipotesi nulla.	
b. Viene utilizzato l'errore standard asintotico presumendo l'ipotesi nulla.	
c. Basato sull'approssimazione normale.	


Stima del rischio	
Esito dopo	Valore	
1	Rapporto odd per 1 Sintomo (0 / 1)	a	
2	Rapporto odd per 1 Sintomo (0 / 1)	a	
Totale	Rapporto odd per 1 Sintomo (0 / 1)	a	

a. Le statistiche di stima del rischio non possono essere calcolate. Vengono calcolate solo per una tabella 2*2 senza celle vuote.	


2 Sintomo * n. sintomi * Esito dopo


Tabella di contingenza	
Conteggio  	
Esito dopo	n. sintomi	Totale	
	2	3	4		
1	2 Sintomo	3	1	0	0	1	
		4	1	0	0	1	
		7	3	0	0	3	
		8	1	0	0	1	
		9	2	2	1	5	
	Totale	8	2	1	11	
2	2 Sintomo	4	1	0		1	
		7	1	0		1	
		8	0	2		2	
		9	1	5		6	
		10	0	1		1	
	Totale	3	8		11	
Totale	2 Sintomo	3	1	0	0	1	
		4	2	0	0	2	
		7	4	0	0	4	
		8	1	2	0	3	
		9	3	7	1	11	
		10	0	1	0	1	
	Totale	11	10	1	22	


Test del chi-quadrato	
Esito dopo	Valore	df	Significatività asintotica (bilaterale)	
1	Chi-quadrato di Pearson	4,950c	8	,763	
	Rapporto di verosimiglianza	6,161	8	,629	
	Associazione lineare per lineare	2,116	1	,146	
	Test McNemar-Bowker	.	.	.b	
	N di casi validi	11			
2	Chi-quadrato di Pearson	6,799d	4	,147	
	Rapporto di verosimiglianza	7,484	4	,112	
	Associazione lineare per lineare	4,064	1	,044	
	Test McNemar-Bowker	.	.	.b	
	N di casi validi	11			
Totale	Chi-quadrato di Pearson	11,236a	10	,339	
	Rapporto di verosimiglianza	14,462	10	,153	
	Associazione lineare per lineare	6,503	1	,011	
	Test McNemar-Bowker	.	.	.b	
	N di casi validi	22			

a. 16 celle (88,9%) hanno un conteggio previsto inferiore a 5. Il conteggio previsto minimo è ,05.	
b. Calcolato solo per una tabella PxP, dove P deve essere maggiore di 1.	
c. 15 celle (100,0%) hanno un conteggio previsto inferiore a 5. Il conteggio previsto minimo è ,09.	
d. 10 celle (100,0%) hanno un conteggio previsto inferiore a 5. Il conteggio previsto minimo è ,27.	


Misure direzionali	
Esito dopo	Valore	Errore standard asintoticoa	T approssimatob	
1	Nominale per nominale	Lambda	Simmetrico	,111	,234	,451	
			2 Sintomo dipendente	,167	,340	,451	
			n. sintomi dipendente	,000	,000	.e	
		Tau di Goodman e Kruskal	2 Sintomo dipendente	,214	,077		
			n. sintomi dipendente	,323	,157		
		Coefficiente di incertezza	Simmetrico	,263	,112	2,020	
			2 Sintomo dipendente	,205	,104	2,020	
			n. sintomi dipendente	,369	,138	2,020	
	Ordinale per ordinale	D di Somers	Simmetrico	,529	,124	2,708	
			2 Sintomo dipendente	,692	,147	2,708	
			n. sintomi dipendente	,429	,175	2,708	
	Nominale per intervallo	Eta	2 Sintomo dipendente	,498			
			n. sintomi dipendente	,620			
2	Nominale per nominale	Lambda	Simmetrico	,250	,258	,842	
			2 Sintomo dipendente	,000	,283	,000	
			n. sintomi dipendente	,667	,272	1,563	
		Tau di Goodman e Kruskal	2 Sintomo dipendente	,119	,097		
			n. sintomi dipendente	,618	,206		
		Coefficiente di incertezza	Simmetrico	,362	,120	2,260	
			2 Sintomo dipendente	,263	,085	2,260	
			n. sintomi dipendente	,581	,231	2,260	
	Ordinale per ordinale	D di Somers	Simmetrico	,476	,227	1,696	
			2 Sintomo dipendente	,625	,314	1,696	
			n. sintomi dipendente	,385	,192	1,696	
	Nominale per intervallo	Eta	2 Sintomo dipendente	,637			
			n. sintomi dipendente	,786			
Totale	Nominale per nominale	Lambda	Simmetrico	,318	,202	1,406	
			2 Sintomo dipendente	,091	,229	,379	
			n. sintomi dipendente	,545	,229	1,706	
		Tau di Goodman e Kruskal	2 Sintomo dipendente	,153	,075		
			n. sintomi dipendente	,415	,111		
		Coefficiente di incertezza	Simmetrico	,289	,074	3,431	
			2 Sintomo dipendente	,230	,059	3,431	
			n. sintomi dipendente	,389	,114	3,431	
	Ordinale per ordinale	D di Somers	Simmetrico	,566	,107	4,645	
			2 Sintomo dipendente	,641	,142	4,645	
			n. sintomi dipendente	,506	,094	4,645	
	Nominale per intervallo	Eta	2 Sintomo dipendente	,590			
			n. sintomi dipendente	,650			

Misure direzionali	
Esito dopo	Significatività approssimata	
1	Nominale per nominale	Lambda	Simmetrico	,652	
			2 Sintomo dipendente	,652	
			n. sintomi dipendente	.e	
		Tau di Goodman e Kruskal	2 Sintomo dipendente	,380c	
			n. sintomi dipendente	,596c	
		Coefficiente di incertezza	Simmetrico	,629d	
			2 Sintomo dipendente	,629d	
			n. sintomi dipendente	,629d	
	Ordinale per ordinale	D di Somers	Simmetrico	,007	
			2 Sintomo dipendente	,007	
			n. sintomi dipendente	,007	
	Nominale per intervallo	Eta	2 Sintomo dipendente		
			n. sintomi dipendente		
2	Nominale per nominale	Lambda	Simmetrico	,400	
			2 Sintomo dipendente	1,000	
			n. sintomi dipendente	,118	
		Tau di Goodman e Kruskal	2 Sintomo dipendente	,315c	
			n. sintomi dipendente	,186c	
		Coefficiente di incertezza	Simmetrico	,112d	
			2 Sintomo dipendente	,112d	
			n. sintomi dipendente	,112d	
	Ordinale per ordinale	D di Somers	Simmetrico	,090	
			2 Sintomo dipendente	,090	
			n. sintomi dipendente	,090	
	Nominale per intervallo	Eta	2 Sintomo dipendente		
			n. sintomi dipendente		
Totale	Nominale per nominale	Lambda	Simmetrico	,160	
			2 Sintomo dipendente	,705	
			n. sintomi dipendente	,088	
		Tau di Goodman e Kruskal	2 Sintomo dipendente	,098c	
			n. sintomi dipendente	,066c	
		Coefficiente di incertezza	Simmetrico	,153d	
			2 Sintomo dipendente	,153d	
			n. sintomi dipendente	,153d	
	Ordinale per ordinale	D di Somers	Simmetrico	,000	
			2 Sintomo dipendente	,000	
			n. sintomi dipendente	,000	
	Nominale per intervallo	Eta	2 Sintomo dipendente		
			n. sintomi dipendente		

a. Non viene assunta l'ipotesi nulla.	
b. Viene utilizzato l'errore standard asintotico presumendo l'ipotesi nulla.	
c. Basato sull'approssimazione chi-quadrato	
d. Probabilità chi-quadrato del rapporto di verosimiglianza.	
e. Non è possibile effettuare il calcolo perché l'errore standard asintotico è uguale a zero.	


Misure simmetriche	
Esito dopo	Valore	Errore standard asintoticoa	
1	Nominale per nominale	Phi	,671		
		V di Cramer	,474		
		Coefficiente di contingenza	,557		
	Ordinale per ordinale	Tau-b di Kendall	,545	,128	
		Tau-c di Kendall	,446	,165	
		Gamma	Ordine zero	1,000	,000	
		Correlazione di Spearman	,609	,147	
	Intervallo per intervallo	R di Pearson	,460	,116	
	Misura di accordo	Kappa	-,025	,019	
	N di casi validi	11		
2	Nominale per nominale	Phi	,786		
		V di Cramer	,786		
		Coefficiente di contingenza	,618		
	Ordinale per ordinale	Tau-b di Kendall	,490	,234	
		Tau-c di Kendall	,496	,292	
		Gamma	Ordine zero	,789	,247	
		Correlazione di Spearman	,529	,260	
	Intervallo per intervallo	R di Pearson	,637	,196	
	Misura di accordo	Kappa	,000	,000	
	N di casi validi	11		
Totale	Nominale per nominale	Phi	,715		
		V di Cramer	,505		
		Coefficiente di contingenza	,581		
	Ordinale per ordinale	Tau-b di Kendall	,570	,108	
		Tau-c di Kendall	,521	,112	
		Gamma	Ordine zero	,857	,105	
			Parziale di ordine 1	,892		
		Correlazione di Spearman	,631	,124	
	Intervallo per intervallo	R di Pearson	,556	,092	
	Misura di accordo	Kappa	-,025	,021	
	N di casi validi	22		

Misure simmetriche	
Esito dopo	T approssimatob	Significatività approssimata	
1	Nominale per nominale	Phi		,763	
		V di Cramer		,763	
		Coefficiente di contingenza		,763	
	Ordinale per ordinale	Tau-b di Kendall	2,708	,007	
		Tau-c di Kendall	2,708	,007	
		Gamma	Ordine zero	2,708	,007	
		Correlazione di Spearman	2,301	,047c	
	Intervallo per intervallo	R di Pearson	1,554	,155c	
	Misura di accordo	Kappa	-,590	,555	
	N di casi validi			
2	Nominale per nominale	Phi		,147	
		V di Cramer		,147	
		Coefficiente di contingenza		,147	
	Ordinale per ordinale	Tau-b di Kendall	1,696	,090	
		Tau-c di Kendall	1,696	,090	
		Gamma	Ordine zero	1,696	,090	
		Correlazione di Spearman	1,872	,094c	
	Intervallo per intervallo	R di Pearson	2,482	,035c	
	Misura di accordo	Kappa	.		
	N di casi validi			
Totale	Nominale per nominale	Phi		,339	
		V di Cramer		,339	
		Coefficiente di contingenza		,339	
	Ordinale per ordinale	Tau-b di Kendall	4,645	,000	
		Tau-c di Kendall	4,645	,000	
		Gamma	Ordine zero	4,645	,000	
			Parziale di ordine 1			
		Correlazione di Spearman	3,640	,002c	
	Intervallo per intervallo	R di Pearson	2,995	,007c	
	Misura di accordo	Kappa	-,965	,334	
	N di casi validi			

a. Non viene assunta l'ipotesi nulla.	
b. Viene utilizzato l'errore standard asintotico presumendo l'ipotesi nulla.	
c. Basato sull'approssimazione normale.	


Stima del rischio	
Esito dopo	Valore	
1	Rapporto odd per 2 Sintomo (3 / 4)	a	
2	Rapporto odd per 2 Sintomo (4 / 7)	a	
Totale	Rapporto odd per 2 Sintomo (3 / 4)	a	

a. Le statistiche di stima del rischio non possono essere calcolate. Vengono calcolate solo per una tabella 2*2 senza celle vuote.	


3 Sintomo * n. sintomi * Esito dopo


Tabella di contingenza	
Conteggio  	
Esito dopo	n. sintomi	Totale	
	3	4		
1	3 Sintomo	2	0	1	1	
		7	2	0	2	
	Totale	2	1	3	
2	3 Sintomo	6	2		2	
		7	6		6	
	Totale	8		8	
Totale	3 Sintomo	2	0	1	1	
		6	2	0	2	
		7	8	0	8	
	Totale	10	1	11	


Test del chi-quadrato	
Esito dopo	Valore	df	Significatività asintotica (bilaterale)	Sign. esatta (bilaterale)	
1	Chi-quadrato di Pearson	3,000c	1	,083		
	Correzione di continuitàd	,188	1	,665		
	Rapporto di verosimiglianza	3,819	1	,051		
	Test esatto di Fisher				,333	
	Associazione lineare per lineare	2,000	1	,157		
	N di casi validi	3				
	Test McNemar				.e	
2	Chi-quadrato di Pearson	.f				
	Test McNemar-Bowker	.	.	.b		
	N di casi validi	8				
Totale	Chi-quadrato di Pearson	11,000a	2	,004		
	Rapporto di verosimiglianza	6,702	2	,035		
	Associazione lineare per lineare	9,290	1	,002		
	Test McNemar-Bowker	.	.	.b		
	N di casi validi	11				

Test del chi-quadrato	
Esito dopo	Sign. esatta (unilaterale)	
1	Chi-quadrato di Pearson		
	Correzione di continuitàd		
	Rapporto di verosimiglianza		
	Test esatto di Fisher	,333	
	Associazione lineare per lineare		
	N di casi validi		
	Test McNemar		
2	Chi-quadrato di Pearson		
	Test McNemar-Bowker		
	N di casi validi		
Totale	Chi-quadrato di Pearson		
	Rapporto di verosimiglianza		
	Associazione lineare per lineare		
	Test McNemar-Bowker		
	N di casi validi		

a. 5 celle (83,3%) hanno un conteggio previsto inferiore a 5. Il conteggio previsto minimo è ,09.	
b. Calcolato solo per una tabella PxP, dove P deve essere maggiore di 1.	
c. 4 celle (100,0%) hanno un conteggio previsto inferiore a 5. Il conteggio previsto minimo è ,33.	
d. Calcolato solo per una tabella 2x2	
e. Entrambe le variabili devono avere valori identici di categorie.	
f. Non viene calcolata alcuna statistica perché n. sintomi è una costante.	


Misure direzionali	
Esito dopo	Valore	
1	Nominale per nominale	Lambda	Simmetrico	1,000	
			3 Sintomo dipendente	1,000	
			n. sintomi dipendente	1,000	
		Tau di Goodman e Kruskal	3 Sintomo dipendente	1,000	
			n. sintomi dipendente	1,000	
		Coefficiente di incertezza	Simmetrico	1,000	
			3 Sintomo dipendente	1,000	
			n. sintomi dipendente	1,000	
	Ordinale per ordinale	D di Somers	Simmetrico	-1,000	
			3 Sintomo dipendente	-1,000	
			n. sintomi dipendente	-1,000	
	Nominale per intervallo	Eta	3 Sintomo dipendente	1,000	
			n. sintomi dipendente	1,000	
2	Nominale per nominale	Lambda	Simmetrico	.e	
Totale	Nominale per nominale	Lambda	Simmetrico	,500	
			3 Sintomo dipendente	,333	
			n. sintomi dipendente	1,000	
		Tau di Goodman e Kruskal	3 Sintomo dipendente	,323	
			n. sintomi dipendente	1,000	
		Coefficiente di incertezza	Simmetrico	,573	
			3 Sintomo dipendente	,401	
			n. sintomi dipendente	1,000	
	Ordinale per ordinale	D di Somers	Simmetrico	-,556	
			3 Sintomo dipendente	-1,000	
			n. sintomi dipendente	-,385	
	Nominale per intervallo	Eta	3 Sintomo dipendente	,964	
			n. sintomi dipendente	1,000	

Misure direzionali	
Esito dopo	Errore standard asintoticoa	
1	Nominale per nominale	Lambda	Simmetrico	,000	
			3 Sintomo dipendente	,000	
			n. sintomi dipendente	,000	
		Tau di Goodman e Kruskal	3 Sintomo dipendente	,000	
			n. sintomi dipendente	,000	
		Coefficiente di incertezza	Simmetrico	,000	
			3 Sintomo dipendente	,000	
			n. sintomi dipendente	,000	
	Ordinale per ordinale	D di Somers	Simmetrico	,000	
			3 Sintomo dipendente	,000	
			n. sintomi dipendente	,000	
	Nominale per intervallo	Eta	3 Sintomo dipendente		
			n. sintomi dipendente		
2	Nominale per nominale	Lambda	Simmetrico		
Totale	Nominale per nominale	Lambda	Simmetrico	,306	
			3 Sintomo dipendente	,272	
			n. sintomi dipendente	,000	
		Tau di Goodman e Kruskal	3 Sintomo dipendente	,123	
			n. sintomi dipendente	,000	
		Coefficiente di incertezza	Simmetrico	,203	
			3 Sintomo dipendente	,199	
			n. sintomi dipendente	,000	
	Ordinale per ordinale	D di Somers	Simmetrico	,197	
			3 Sintomo dipendente	,000	
			n. sintomi dipendente	,272	
	Nominale per intervallo	Eta	3 Sintomo dipendente		
			n. sintomi dipendente		

Misure direzionali	
Esito dopo	T approssimatob	
1	Nominale per nominale	Lambda	Simmetrico	1,225	
			3 Sintomo dipendente	1,225	
			n. sintomi dipendente	1,225	
		Tau di Goodman e Kruskal	3 Sintomo dipendente		
			n. sintomi dipendente		
		Coefficiente di incertezza	Simmetrico	3,374	
			3 Sintomo dipendente	3,374	
			n. sintomi dipendente	3,374	
	Ordinale per ordinale	D di Somers	Simmetrico	-2,449	
			3 Sintomo dipendente	-2,449	
			n. sintomi dipendente	-2,449	
	Nominale per intervallo	Eta	3 Sintomo dipendente		
			n. sintomi dipendente		
2	Nominale per nominale	Lambda	Simmetrico		
Totale	Nominale per nominale	Lambda	Simmetrico	1,049	
			3 Sintomo dipendente	1,049	
			n. sintomi dipendente	1,049	
		Tau di Goodman e Kruskal	3 Sintomo dipendente		
			n. sintomi dipendente		
		Coefficiente di incertezza	Simmetrico	1,526	
			3 Sintomo dipendente	1,526	
			n. sintomi dipendente	1,526	
	Ordinale per ordinale	D di Somers	Simmetrico	-1,165	
			3 Sintomo dipendente	-1,165	
			n. sintomi dipendente	-1,165	
	Nominale per intervallo	Eta	3 Sintomo dipendente		
			n. sintomi dipendente		

Misure direzionali	
Esito dopo	Significatività approssimata	
1	Nominale per nominale	Lambda	Simmetrico	,221	
			3 Sintomo dipendente	,221	
			n. sintomi dipendente	,221	
		Tau di Goodman e Kruskal	3 Sintomo dipendente	,157c	
			n. sintomi dipendente	,157c	
		Coefficiente di incertezza	Simmetrico	,051d	
			3 Sintomo dipendente	,051d	
			n. sintomi dipendente	,051d	
	Ordinale per ordinale	D di Somers	Simmetrico	,014	
			3 Sintomo dipendente	,014	
			n. sintomi dipendente	,014	
	Nominale per intervallo	Eta	3 Sintomo dipendente		
			n. sintomi dipendente		
2	Nominale per nominale	Lambda	Simmetrico		
Totale	Nominale per nominale	Lambda	Simmetrico	,294	
			3 Sintomo dipendente	,294	
			n. sintomi dipendente	,294	
		Tau di Goodman e Kruskal	3 Sintomo dipendente	,040c	
			n. sintomi dipendente	,007c	
		Coefficiente di incertezza	Simmetrico	,035d	
			3 Sintomo dipendente	,035d	
			n. sintomi dipendente	,035d	
	Ordinale per ordinale	D di Somers	Simmetrico	,244	
			3 Sintomo dipendente	,244	
			n. sintomi dipendente	,244	
	Nominale per intervallo	Eta	3 Sintomo dipendente		
			n. sintomi dipendente		

a. Non viene assunta l'ipotesi nulla.	
b. Viene utilizzato l'errore standard asintotico presumendo l'ipotesi nulla.	
c. Basato sull'approssimazione chi-quadrato	
d. Probabilità chi-quadrato del rapporto di verosimiglianza.	
e. Non viene calcolata alcuna statistica perché n. sintomi è una costante.	


Misure simmetriche	
Esito dopo	Valore	Errore standard asintoticoa	
1	Nominale per nominale	Phi	-1,000		
		V di Cramer	1,000		
		Coefficiente di contingenza	,707		
	Ordinale per ordinale	Tau-b di Kendall	-1,000	,000	
		Tau-c di Kendall	-,889	,363	
		Gamma	Ordine zero	-1,000	,000	
		Correlazione di Spearman	-1,000	,000c	
	Intervallo per intervallo	R di Pearson	-1,000	,000	
	Misura di accordo	Kappa	,000	,000	
	N di casi validi	3		
2	Nominale per nominale	Phi	.d		
	Misura di accordo	Kappa	,000	,000	
	N di casi validi	8		
Totale	Nominale per nominale	Phi	1,000		
		V di Cramer	1,000		
		Coefficiente di contingenza	,707		
	Ordinale per ordinale	Tau-b di Kendall	-,620	,220	
		Tau-c di Kendall	-,331	,284	
		Gamma	Ordine zero	-1,000	,000	
			Parziale di ordine 1	-1,000		
		Correlazione di Spearman	-,638	,234	
	Intervallo per intervallo	R di Pearson	-,964	,037	
	Misura di accordo	Kappa	,000	,000	
	N di casi validi	11		

Misure simmetriche	
Esito dopo	T approssimatob	
1	Nominale per nominale	Phi		
		V di Cramer		
		Coefficiente di contingenza		
	Ordinale per ordinale	Tau-b di Kendall	-2,449	
		Tau-c di Kendall	-2,449	
		Gamma	Ordine zero	-2,449	
		Correlazione di Spearman		
	Intervallo per intervallo	R di Pearson	-94906265,624	
	Misura di accordo	Kappa	.	
	N di casi validi		
2	Nominale per nominale	Phi		
	Misura di accordo	Kappa	.	
	N di casi validi		
Totale	Nominale per nominale	Phi		
		V di Cramer		
		Coefficiente di contingenza		
	Ordinale per ordinale	Tau-b di Kendall	-1,165	
		Tau-c di Kendall	-1,165	
		Gamma	Ordine zero	-1,165	
			Parziale di ordine 1		
		Correlazione di Spearman	-2,487	
	Intervallo per intervallo	R di Pearson	-10,854	
	Misura di accordo	Kappa	.	
	N di casi validi		

Misure simmetriche	
Esito dopo	Significatività approssimata	
1	Nominale per nominale	Phi	,083	
		V di Cramer	,083	
		Coefficiente di contingenza	,083	
	Ordinale per ordinale	Tau-b di Kendall	,014	
		Tau-c di Kendall	,014	
		Gamma	Ordine zero	,014	
		Correlazione di Spearman		
	Intervallo per intervallo	R di Pearson	,000c	
	Misura di accordo	Kappa		
	N di casi validi		
2	Nominale per nominale	Phi		
	Misura di accordo	Kappa		
	N di casi validi		
Totale	Nominale per nominale	Phi	,004	
		V di Cramer	,004	
		Coefficiente di contingenza	,004	
	Ordinale per ordinale	Tau-b di Kendall	,244	
		Tau-c di Kendall	,244	
		Gamma	Ordine zero	,244	
			Parziale di ordine 1		
		Correlazione di Spearman	,035c	
	Intervallo per intervallo	R di Pearson	,000c	
	Misura di accordo	Kappa		
	N di casi validi		

a. Non viene assunta l'ipotesi nulla.	
b. Viene utilizzato l'errore standard asintotico presumendo l'ipotesi nulla.	
c. Basato sull'approssimazione normale.	
d. Non viene calcolata alcuna statistica perché n. sintomi è una costante.	


Stima del rischio	
Esito dopo	Valore	
1	Rapporto odd per 3 Sintomo (2 / 7)	a	
2	Rapporto odd per 3 Sintomo (6 / 7)	.b	
Totale	Rapporto odd per 3 Sintomo (2 / 6)	a	

a. Le statistiche di stima del rischio non possono essere calcolate. Vengono calcolate solo per una tabella 2*2 senza celle vuote.	
b. Non viene calcolata alcuna statistica perché n. sintomi è una costante.	


4 Sintomo * n. sintomi * Esito dopo


Tabella di contingenza	
Conteggio  	
Esito dopo	n. sintomi	Totale	
	4		
1	4 Sintomo	7	1	1	
	Totale	1	1	
Totale	4 Sintomo	7	1	1	
	Totale	1	1	


Test del chi-quadrato	
Esito dopo	Valore	df	Significatività asintotica (bilaterale)	
1	Chi-quadrato di Pearson	.a			
	Test McNemar-Bowker	.	.	.b	
	N di casi validi	1			
Totale	Chi-quadrato di Pearson	.a			
	Test McNemar-Bowker	.	.	.b	
	N di casi validi	1			

a. Non viene calcolata alcuna statistica perché 4 Sintomo e n. sintomi sono costanti.	
b. Calcolato solo per una tabella PxP, dove P deve essere maggiore di 1.	


Misure direzionali	
Esito dopo	Valore	
1	Nominale per nominale	Lambda	Simmetrico	.a	
Totale	Nominale per nominale	Lambda	Simmetrico	.a	

a. Non viene calcolata alcuna statistica perché 4 Sintomo e n. sintomi sono costanti.	


Misure simmetriche	
Esito dopo	Valore	Errore standard asintoticob	T approssimatoc	
1	Nominale per nominale	Phi	.a			
	Misura di accordo	Kappa	,000	,000	.	
	N di casi validi	1			
Totale	Nominale per nominale	Phi	.a			
	Misura di accordo	Kappa	,000	,000	.	
	N di casi validi	1			

a. Non viene calcolata alcuna statistica perché 4 Sintomo e n. sintomi sono costanti.	
b. Non viene assunta l'ipotesi nulla.	
c. Viene utilizzato l'errore standard asintotico presumendo l'ipotesi nulla.	


Stima del rischio	
Esito dopo	Valore	
1	Rapporto odd per 4 Sintomo (7 / .)	.a	
Totale	Rapporto odd per 4 Sintomo (7 / .)	.a	

a. Non viene calcolata alcuna statistica perché 4 Sintomo e n. sintomi sono costanti.	

CROSSTABS
  /TABLES=n.sintomi BY Esitodopo
  /FORMAT=AVALUE TABLES
  /STATISTICS=CHISQ CC PHI LAMBDA UC ETA CORR GAMMA D BTAU CTAU KAPPA RISK MCNEMAR CMH(1)
  /CELLS=COUNT
  /COUNT ROUND CELL.


Tabelle di contingenza


Note	
Output creato	30-NOV-2020 12:58:10	
Commenti		
Input	Dataset attivo	Dataset1	
	Filtro	<nessuno>	
	Peso	<nessuno>	
	Suddividi file	<nessuno>	
	N di righe nel file di dati di lavoro	54	
Gestione valori mancanti	Definizione di mancante	I valori mancanti definiti dall'utente vengono trattati come mancanti.	
	Casi utilizzati	Le statistiche per ciascuna tabella sono basate su tutti i casi con dati validi nell'intervallo o negli intervalli specificati per tutte le variabili in ciascuna tabella.	
Sintassi	CROSSTABS
  /TABLES=n.sintomi BY Esitodopo
  /FORMAT=AVALUE TABLES
  /STATISTICS=CHISQ CC PHI LAMBDA UC ETA CORR GAMMA D BTAU CTAU KAPPA RISK MCNEMAR CMH(1)
  /CELLS=COUNT
  /COUNT ROUND CELL.	
Risorse	Tempo processore	00:00:00,00	
	Tempo trascorso	00:00:00,00	
	Dimensioni richieste	2	
	Celle disponibili	524245	


Avvisi	
La tabella di test di omogeneità del rapporto odd e la tabella di stima del rapporto odd comune di Mantel-Haenszel non vengono calcolate per n. sintomi * Esito dopo, perché (1) la variabile di gruppo non ha esattamente due valori non mancanti distinti o/e (2) la variabile di risposta non ha esattamente due valori non mancanti distinti.	


Riepilogo elaborazione casi	
	Casi	
	Valido	Mancante	Totale	
	N	Percentuale	N	Percentuale	N	
n. sintomi * Esito dopo	54	100,0%	0	0,0%	54	

Riepilogo elaborazione casi	
	Casi	
	Totale	
	Percentuale	
n. sintomi * Esito dopo	100,0%	


Tavola di contingenza n. sintomi * Esito dopo	
Conteggio  	
	Esito dopo	Totale	
	1	2		
n. sintomi	0	4	17	21	
	1	4	7	11	
	2	8	3	11	
	3	2	8	10	
	4	1	0	1	
Totale	19	35	54	


Test del chi-quadrato	
	Valore	df	Significatività asintotica (bilaterale)	
Chi-quadrato di Pearson	12,056a	4	,017	
Rapporto di verosimiglianza	12,277	4	,015	
Associazione lineare per lineare	2,282	1	,131	
Test McNemar-Bowker	.	.	.b	
N di casi validi	54			

a. 5 celle (50,0%) hanno un conteggio previsto inferiore a 5. Il conteggio previsto minimo è ,35.	
b. Calcolato solo per una tabella PxP, dove P deve essere maggiore di 1.	


Misure direzionali	
	Valore	
Nominale per nominale	Lambda	Simmetrico	,192	
		n. sintomi dipendente	,121	
		Esito dopo dipendente	,316	
	Tau di Goodman e Kruskal	n. sintomi dipendente	,064	
		Esito dopo dipendente	,223	
	Coefficiente di incertezza	Simmetrico	,111	
		n. sintomi dipendente	,081	
		Esito dopo dipendente	,175	
Ordinale per ordinale	D di Somers	Simmetrico	-,195	
		n. sintomi dipendente	-,254	
		Esito dopo dipendente	-,159	
Nominale per intervallo	Eta	n. sintomi dipendente	,208	
		Esito dopo dipendente	,473	

Misure direzionali	
	Errore standard asintoticoa	
Nominale per nominale	Lambda	Simmetrico	,110	
		n. sintomi dipendente	,098	
		Esito dopo dipendente	,151	
	Tau di Goodman e Kruskal	n. sintomi dipendente	,040	
		Esito dopo dipendente	,107	
	Coefficiente di incertezza	Simmetrico	,057	
		n. sintomi dipendente	,041	
		Esito dopo dipendente	,091	
Ordinale per ordinale	D di Somers	Simmetrico	,116	
		n. sintomi dipendente	,150	
		Esito dopo dipendente	,095	
Nominale per intervallo	Eta	n. sintomi dipendente		
		Esito dopo dipendente		

Misure direzionali	
	T approssimatob	
Nominale per nominale	Lambda	Simmetrico	1,619	
		n. sintomi dipendente	1,169	
		Esito dopo dipendente	1,782	
	Tau di Goodman e Kruskal	n. sintomi dipendente		
		Esito dopo dipendente		
	Coefficiente di incertezza	Simmetrico	1,922	
		n. sintomi dipendente	1,922	
		Esito dopo dipendente	1,922	
Ordinale per ordinale	D di Somers	Simmetrico	-1,682	
		n. sintomi dipendente	-1,682	
		Esito dopo dipendente	-1,682	
Nominale per intervallo	Eta	n. sintomi dipendente		
		Esito dopo dipendente		

Misure direzionali	
	Significatività approssimata	
Nominale per nominale	Lambda	Simmetrico	,105	
		n. sintomi dipendente	,242	
		Esito dopo dipendente	,075	
	Tau di Goodman e Kruskal	n. sintomi dipendente	,009c	
		Esito dopo dipendente	,019c	
	Coefficiente di incertezza	Simmetrico	,015d	
		n. sintomi dipendente	,015d	
		Esito dopo dipendente	,015d	
Ordinale per ordinale	D di Somers	Simmetrico	,093	
		n. sintomi dipendente	,093	
		Esito dopo dipendente	,093	
Nominale per intervallo	Eta	n. sintomi dipendente		
		Esito dopo dipendente		

a. Non viene assunta l'ipotesi nulla.	
b. Viene utilizzato l'errore standard asintotico presumendo l'ipotesi nulla.	
c. Basato sull'approssimazione chi-quadrato	
d. Probabilità chi-quadrato del rapporto di verosimiglianza.	


Misure simmetriche	
	Valore	Errore standard asintoticoa	T approssimatob	
Nominale per nominale	Phi	,473			
	V di Cramer	,473			
	Coefficiente di contingenza	,427			
Ordinale per ordinale	Tau-b di Kendall	-,201	,119	-1,682	
	Tau-c di Kendall	-,232	,138	-1,682	
	Gamma	-,319	,181	-1,682	
	Correlazione di Spearman	-,219	,130	-1,622	
Intervallo per intervallo	R di Pearson	-,208	,128	-1,530	
Misura di accordo	Kappa	-,093	,059	-1,786	
N di casi validi	54			

Misure simmetriche	
	Significatività approssimata	
Nominale per nominale	Phi	,017	
	V di Cramer	,017	
	Coefficiente di contingenza	,017	
Ordinale per ordinale	Tau-b di Kendall	,093	
	Tau-c di Kendall	,093	
	Gamma	,093	
	Correlazione di Spearman	,111c	
Intervallo per intervallo	R di Pearson	,132c	
Misura di accordo	Kappa	,074	
N di casi validi		

a. Non viene assunta l'ipotesi nulla.	
b. Viene utilizzato l'errore standard asintotico presumendo l'ipotesi nulla.	
c. Basato sull'approssimazione normale.	


Stima del rischio	
	Valore	
Rapporto odd per n. sintomi (0 / 1)	a	

a. Le statistiche di stima del rischio non possono essere calcolate. Vengono calcolate solo per una tabella 2*2 senza celle vuote.	

CROSSTABS
  /TABLES=n.sintomi BY Esitodopo
  /FORMAT=AVALUE TABLES
  /STATISTICS=CHISQ CC PHI LAMBDA UC CORR
  /CELLS=COUNT
  /COUNT ROUND CELL.


Tabelle di contingenza


Note	
Output creato	30-NOV-2020 13:28:20	
Commenti		
Input	Dataset attivo	Dataset1	
	Filtro	<nessuno>	
	Peso	<nessuno>	
	Suddividi file	<nessuno>	
	N di righe nel file di dati di lavoro	54	
Gestione valori mancanti	Definizione di mancante	I valori mancanti definiti dall'utente vengono trattati come mancanti.	
	Casi utilizzati	Le statistiche per ciascuna tabella sono basate su tutti i casi con dati validi nell'intervallo o negli intervalli specificati per tutte le variabili in ciascuna tabella.	
Sintassi	CROSSTABS
  /TABLES=n.sintomi BY Esitodopo
  /FORMAT=AVALUE TABLES
  /STATISTICS=CHISQ CC PHI LAMBDA UC CORR
  /CELLS=COUNT
  /COUNT ROUND CELL.	
Risorse	Tempo processore	00:00:00,02	
	Tempo trascorso	00:00:00,02	
	Dimensioni richieste	2	
	Celle disponibili	524245	


Riepilogo elaborazione casi	
	Casi	
	Valido	Mancante	Totale	
	N	Percentuale	N	Percentuale	N	
n. sintomi * Esito dopo	54	100,0%	0	0,0%	54	

Riepilogo elaborazione casi	
	Casi	
	Totale	
	Percentuale	
n. sintomi * Esito dopo	100,0%	


Tavola di contingenza n. sintomi * Esito dopo	
Conteggio  	
	Esito dopo	Totale	
	1	2		
n. sintomi	0	4	17	21	
	1	4	7	11	
	2	8	3	11	
	3	2	8	10	
	4	1	0	1	
Totale	19	35	54	


Test del chi-quadrato	
	Valore	df	Significatività asintotica (bilaterale)	
Chi-quadrato di Pearson	12,056a	4	,017	
Rapporto di verosimiglianza	12,277	4	,015	
Associazione lineare per lineare	2,282	1	,131	
N di casi validi	54			

a. 5 celle (50,0%) hanno un conteggio previsto inferiore a 5. Il conteggio previsto minimo è ,35.	


Misure direzionali	
	Valore	
Nominale per nominale	Lambda	Simmetrico	,192	
		n. sintomi dipendente	,121	
		Esito dopo dipendente	,316	
	Tau di Goodman e Kruskal	n. sintomi dipendente	,064	
		Esito dopo dipendente	,223	
	Coefficiente di incertezza	Simmetrico	,111	
		n. sintomi dipendente	,081	
		Esito dopo dipendente	,175	

Misure direzionali	
	Errore standard asintoticoa	
Nominale per nominale	Lambda	Simmetrico	,110	
		n. sintomi dipendente	,098	
		Esito dopo dipendente	,151	
	Tau di Goodman e Kruskal	n. sintomi dipendente	,040	
		Esito dopo dipendente	,107	
	Coefficiente di incertezza	Simmetrico	,057	
		n. sintomi dipendente	,041	
		Esito dopo dipendente	,091	

Misure direzionali	
	T approssimatob	
Nominale per nominale	Lambda	Simmetrico	1,619	
		n. sintomi dipendente	1,169	
		Esito dopo dipendente	1,782	
	Tau di Goodman e Kruskal	n. sintomi dipendente		
		Esito dopo dipendente		
	Coefficiente di incertezza	Simmetrico	1,922	
		n. sintomi dipendente	1,922	
		Esito dopo dipendente	1,922	

Misure direzionali	
	Significatività approssimata	
Nominale per nominale	Lambda	Simmetrico	,105	
		n. sintomi dipendente	,242	
		Esito dopo dipendente	,075	
	Tau di Goodman e Kruskal	n. sintomi dipendente	,009c	
		Esito dopo dipendente	,019c	
	Coefficiente di incertezza	Simmetrico	,015d	
		n. sintomi dipendente	,015d	
		Esito dopo dipendente	,015d	

a. Non viene assunta l'ipotesi nulla.	
b. Viene utilizzato l'errore standard asintotico presumendo l'ipotesi nulla.	
c. Basato sull'approssimazione chi-quadrato	
d. Probabilità chi-quadrato del rapporto di verosimiglianza.	


Misure simmetriche	
	Valore	Errore standard asintoticoa	T approssimatob	
Nominale per nominale	Phi	,473			
	V di Cramer	,473			
	Coefficiente di contingenza	,427			
Intervallo per intervallo	R di Pearson	-,208	,128	-1,530	
Ordinale per ordinale	Correlazione di Spearman	-,219	,130	-1,622	
N di casi validi	54			

Misure simmetriche	
	Significatività approssimata	
Nominale per nominale	Phi	,017	
	V di Cramer	,017	
	Coefficiente di contingenza	,017	
Intervallo per intervallo	R di Pearson	,132c	
Ordinale per ordinale	Correlazione di Spearman	,111c	
N di casi validi		

a. Non viene assunta l'ipotesi nulla.	
b. Viene utilizzato l'errore standard asintotico presumendo l'ipotesi nulla.	
c. Basato sull'approssimazione normale.	

FREQUENCIES VARIABLES=PregressaQuarantena
  /STATISTICS=STDDEV MINIMUM MAXIMUM MEAN MEDIAN
  /ORDER=ANALYSIS.


Frequenze


Note	
Output creato	01-DEC-2020 09:06:26	
Commenti		
Input	Dataset attivo	Dataset1	
	Filtro	<nessuno>	
	Peso	<nessuno>	
	Suddividi file	<nessuno>	
	N di righe nel file di dati di lavoro	54	
Gestione valori mancanti	Definizione di mancante	I valori mancanti definiti dall'utente vengono trattati come mancanti.	
	Casi utilizzati	Le statistiche sono basate su tutti i casi con dati validi.	
Sintassi	FREQUENCIES VARIABLES=PregressaQuarantena
  /STATISTICS=STDDEV MINIMUM MAXIMUM MEAN MEDIAN
  /ORDER=ANALYSIS.	
Risorse	Tempo processore	00:00:00,02	
	Tempo trascorso	00:00:00,02	


Statistiche	
PregressaQuarantena  	
N	Valido	54	
	Mancante	0	
Media	1,85	
Mediana	2,00	
Deviazione std.	,359	
Minimo	1	
Massimo	2	


PregressaQuarantena	
	Frequenza	Percentuale	Percentuale valida	Percentuale cumulativa	
Valido	1	8	14,8	14,8	14,8	
	2	46	85,2	85,2	100,0	
	Totale	54	100,0	100,0		

FREQUENCIES VARIABLES=ContattiContagi
  /STATISTICS=STDDEV MINIMUM MAXIMUM MEAN MEDIAN
  /ORDER=ANALYSIS.


Frequenze


Note	
Output creato	01-DEC-2020 09:07:55	
Commenti		
Input	Dataset attivo	Dataset1	
	Filtro	<nessuno>	
	Peso	<nessuno>	
	Suddividi file	<nessuno>	
	N di righe nel file di dati di lavoro	54	
Gestione valori mancanti	Definizione di mancante	I valori mancanti definiti dall'utente vengono trattati come mancanti.	
	Casi utilizzati	Le statistiche sono basate su tutti i casi con dati validi.	
Sintassi	FREQUENCIES VARIABLES=ContattiContagi
  /STATISTICS=STDDEV MINIMUM MAXIMUM MEAN MEDIAN
  /ORDER=ANALYSIS.	
Risorse	Tempo processore	00:00:00,00	
	Tempo trascorso	00:00:00,00	


Statistiche	
ContattiContagi  	
N	Valido	54	
	Mancante	0	
Media	1,00	
Mediana	1,00	
Deviazione std.	,194	
Minimo	0	
Massimo	2	


ContattiContagi	
	Frequenza	Percentuale	Percentuale valida	Percentuale cumulativa	
Valido	0	1	1,9	1,9	1,9	
	1	52	96,3	96,3	98,1	
	2	1	1,9	1,9	100,0	
	Totale	54	100,0	100,0		

FREQUENCIES VARIABLES=ESITOprima
  /STATISTICS=STDDEV MINIMUM MAXIMUM MEAN MEDIAN
  /ORDER=ANALYSIS.


Frequenze


Note	
Output creato	01-DEC-2020 09:10:29	
Commenti		
Input	Dataset attivo	Dataset1	
	Filtro	<nessuno>	
	Peso	<nessuno>	
	Suddividi file	<nessuno>	
	N di righe nel file di dati di lavoro	54	
Gestione valori mancanti	Definizione di mancante	I valori mancanti definiti dall'utente vengono trattati come mancanti.	
	Casi utilizzati	Le statistiche sono basate su tutti i casi con dati validi.	
Sintassi	FREQUENCIES VARIABLES=ESITOprima
  /STATISTICS=STDDEV MINIMUM MAXIMUM MEAN MEDIAN
  /ORDER=ANALYSIS.	
Risorse	Tempo processore	00:00:00,00	
	Tempo trascorso	00:00:00,00	


Statistiche	
ESITO prima  	
N	Valido	54	
	Mancante	0	
Media	,30	
Mediana	,00	
Deviazione std.	,461	
Minimo	0	
Massimo	1	


ESITO prima	
	Frequenza	Percentuale	Percentuale valida	Percentuale cumulativa	
Valido	0	38	70,4	70,4	70,4	
	1	16	29,6	29,6	100,0	
	Totale	54	100,0	100,0		

FREQUENCIES VARIABLES=Esitodopo
  /STATISTICS=STDDEV MINIMUM MAXIMUM MEAN MEDIAN
  /ORDER=ANALYSIS.


Frequenze


Note	
Output creato	01-DEC-2020 09:12:34	
Commenti		
Input	Dataset attivo	Dataset1	
	Filtro	<nessuno>	
	Peso	<nessuno>	
	Suddividi file	<nessuno>	
	N di righe nel file di dati di lavoro	54	
Gestione valori mancanti	Definizione di mancante	I valori mancanti definiti dall'utente vengono trattati come mancanti.	
	Casi utilizzati	Le statistiche sono basate su tutti i casi con dati validi.	
Sintassi	FREQUENCIES VARIABLES=Esitodopo
  /STATISTICS=STDDEV MINIMUM MAXIMUM MEAN MEDIAN
  /ORDER=ANALYSIS.	
Risorse	Tempo processore	00:00:00,00	
	Tempo trascorso	00:00:00,00	


Statistiche	
Esito dopo  	
N	Valido	54	
	Mancante	0	
Media	1,65	
Mediana	2,00	
Deviazione std.	,482	
Minimo	1	
Massimo	2	


Esito dopo	
	Frequenza	Percentuale	Percentuale valida	Percentuale cumulativa	
Valido	1	19	35,2	35,2	35,2	
	2	35	64,8	64,8	100,0	
	Totale	54	100,0	100,0		

FREQUENCIES VARIABLES=Etàgruppo RelazioneNucleo
  /STATISTICS=STDDEV MINIMUM MAXIMUM MEAN MEDIAN
  /ORDER=ANALYSIS.


Frequenze


Note	
Output creato	01-DEC-2020 09:27:19	
Commenti		
Input	Dataset attivo	Dataset1	
	Filtro	<nessuno>	
	Peso	<nessuno>	
	Suddividi file	<nessuno>	
	N di righe nel file di dati di lavoro	54	
Gestione valori mancanti	Definizione di mancante	I valori mancanti definiti dall'utente vengono trattati come mancanti.	
	Casi utilizzati	Le statistiche sono basate su tutti i casi con dati validi.	
Sintassi	FREQUENCIES VARIABLES=Etàgruppo RelazioneNucleo
  /STATISTICS=STDDEV MINIMUM MAXIMUM MEAN MEDIAN
  /ORDER=ANALYSIS.	
Risorse	Tempo processore	00:00:00,00	
	Tempo trascorso	00:00:00,00	


Statistiche	
	Età gruppo	RelazioneNucleo	
N	Valido	54	54	
	Mancante	0	0	
Media	3,57	3,57	
Mediana	4,00	2,00	
Deviazione std.	1,899	3,124	
Minimo	0	0	
Massimo	7	8	


Tabella delle frequenze


Età gruppo	
	Frequenza	Percentuale	Percentuale valida	Percentuale cumulativa	
Valido	0	1	1,9	1,9	1,9	
	1	9	16,7	16,7	18,5	
	2	10	18,5	18,5	37,0	
	3	6	11,1	11,1	48,1	
	4	5	9,3	9,3	57,4	
	5	13	24,1	24,1	81,5	
	6	9	16,7	16,7	98,1	
	7	1	1,9	1,9	100,0	
	Totale	54	100,0	100,0		


RelazioneNucleo	
	Frequenza	Percentuale	Percentuale valida	Percentuale cumulativa	
Valido	0	1	1,9	1,9	1,9	
	1	25	46,3	46,3	48,1	
	2	7	13,0	13,0	61,1	
	3	1	1,9	1,9	63,0	
	7	9	16,7	16,7	79,6	
	8	11	20,4	20,4	100,0	
	Totale	54	100,0	100,0		

FREQUENCIES VARIABLES=Etàgruppo
  /STATISTICS=STDDEV MINIMUM MAXIMUM MEAN MEDIAN
  /ORDER=ANALYSIS.


Frequenze


Note	
Output creato	01-DEC-2020 09:27:58	
Commenti		
Input	Dataset attivo	Dataset1	
	Filtro	<nessuno>	
	Peso	<nessuno>	
	Suddividi file	<nessuno>	
	N di righe nel file di dati di lavoro	54	
Gestione valori mancanti	Definizione di mancante	I valori mancanti definiti dall'utente vengono trattati come mancanti.	
	Casi utilizzati	Le statistiche sono basate su tutti i casi con dati validi.	
Sintassi	FREQUENCIES VARIABLES=Etàgruppo
  /STATISTICS=STDDEV MINIMUM MAXIMUM MEAN MEDIAN
  /ORDER=ANALYSIS.	
Risorse	Tempo processore	00:00:00,00	
	Tempo trascorso	00:00:00,00	


Statistiche	
Età gruppo  	
N	Valido	54	
	Mancante	0	
Media	3,57	
Mediana	4,00	
Deviazione std.	1,899	
Minimo	0	
Massimo	7	


Età gruppo	
	Frequenza	Percentuale	Percentuale valida	Percentuale cumulativa	
Valido	0	1	1,9	1,9	1,9	
	1	9	16,7	16,7	18,5	
	2	10	18,5	18,5	37,0	
	3	6	11,1	11,1	48,1	
	4	5	9,3	9,3	57,4	
	5	13	24,1	24,1	81,5	
	6	9	16,7	16,7	98,1	
	7	1	1,9	1,9	100,0	
	Totale	54	100,0	100,0		

CROSSTABS
  /TABLES=Etàgruppo BY Esitodopo
  /FORMAT=AVALUE TABLES
  /STATISTICS=CHISQ CC PHI LAMBDA UC CORR
  /CELLS=COUNT
  /COUNT ROUND CELL.


Tabelle di contingenza


Note	
Output creato	01-DEC-2020 09:28:40	
Commenti		
Input	Dataset attivo	Dataset1	
	Filtro	<nessuno>	
	Peso	<nessuno>	
	Suddividi file	<nessuno>	
	N di righe nel file di dati di lavoro	54	
Gestione valori mancanti	Definizione di mancante	I valori mancanti definiti dall'utente vengono trattati come mancanti.	
	Casi utilizzati	Le statistiche per ciascuna tabella sono basate su tutti i casi con dati validi nell'intervallo o negli intervalli specificati per tutte le variabili in ciascuna tabella.	
Sintassi	CROSSTABS
  /TABLES=Etàgruppo BY Esitodopo
  /FORMAT=AVALUE TABLES
  /STATISTICS=CHISQ CC PHI LAMBDA UC CORR
  /CELLS=COUNT
  /COUNT ROUND CELL.	
Risorse	Tempo processore	00:00:00,02	
	Tempo trascorso	00:00:00,01	
	Dimensioni richieste	2	
	Celle disponibili	524245	


Riepilogo elaborazione casi	
	Casi	
	Valido	Mancante	Totale	
	N	Percentuale	N	Percentuale	N	
Età gruppo * Esito dopo	54	100,0%	0	0,0%	54	

Riepilogo elaborazione casi	
	Casi	
	Totale	
	Percentuale	
Età gruppo * Esito dopo	100,0%	


Tavola di contingenza Età gruppo * Esito dopo	
Conteggio  	
	Esito dopo	Totale	
	1	2		
Età gruppo	0	0	1	1	
	1	2	7	9	
	2	2	8	10	
	3	2	4	6	
	4	3	2	5	
	5	4	9	13	
	6	6	3	9	
	7	0	1	1	
Totale	19	35	54	


Test del chi-quadrato	
	Valore	df	Significatività asintotica (bilaterale)	
Chi-quadrato di Pearson	8,142a	7	,320	
Rapporto di verosimiglianza	8,631	7	,280	
Associazione lineare per lineare	3,291	1	,070	
N di casi validi	54			

a. 12 celle (75,0%) hanno un conteggio previsto inferiore a 5. Il conteggio previsto minimo è ,35.	


Misure direzionali	
	Valore	
Nominale per nominale	Lambda	Simmetrico	,100	
		Età gruppo dipendente	,049	
		Esito dopo dipendente	,211	
	Tau di Goodman e Kruskal	Età gruppo dipendente	,025	
		Esito dopo dipendente	,151	
	Coefficiente di incertezza	Simmetrico	,064	
		Età gruppo dipendente	,043	
		Esito dopo dipendente	,123	

Misure direzionali	
	Errore standard asintoticoa	
Nominale per nominale	Lambda	Simmetrico	,095	
		Età gruppo dipendente	,075	
		Esito dopo dipendente	,175	
	Tau di Goodman e Kruskal	Età gruppo dipendente	,019	
		Esito dopo dipendente	,093	
	Coefficiente di incertezza	Simmetrico	,038	
		Età gruppo dipendente	,026	
		Esito dopo dipendente	,074	

Misure direzionali	
	T approssimatob	
Nominale per nominale	Lambda	Simmetrico	1,009	
		Età gruppo dipendente	,635	
		Esito dopo dipendente	1,081	
	Tau di Goodman e Kruskal	Età gruppo dipendente		
		Esito dopo dipendente		
	Coefficiente di incertezza	Simmetrico	1,643	
		Età gruppo dipendente	1,643	
		Esito dopo dipendente	1,643	

Misure direzionali	
	Significatività approssimata	
Nominale per nominale	Lambda	Simmetrico	,313	
		Età gruppo dipendente	,526	
		Esito dopo dipendente	,280	
	Tau di Goodman e Kruskal	Età gruppo dipendente	,233c	
		Esito dopo dipendente	,333c	
	Coefficiente di incertezza	Simmetrico	,280d	
		Età gruppo dipendente	,280d	
		Esito dopo dipendente	,280d	

a. Non viene assunta l'ipotesi nulla.	
b. Viene utilizzato l'errore standard asintotico presumendo l'ipotesi nulla.	
c. Basato sull'approssimazione chi-quadrato	
d. Probabilità chi-quadrato del rapporto di verosimiglianza.	


Misure simmetriche	
	Valore	Errore standard asintoticoa	T approssimatob	
Nominale per nominale	Phi	,388			
	V di Cramer	,388			
	Coefficiente di contingenza	,362			
Intervallo per intervallo	R di Pearson	-,249	,127	-1,856	
Ordinale per ordinale	Correlazione di Spearman	-,254	,128	-1,894	
N di casi validi	54			

Misure simmetriche	
	Significatività approssimata	
Nominale per nominale	Phi	,320	
	V di Cramer	,320	
	Coefficiente di contingenza	,320	
Intervallo per intervallo	R di Pearson	,069c	
Ordinale per ordinale	Correlazione di Spearman	,064c	
N di casi validi		

a. Non viene assunta l'ipotesi nulla.	
b. Viene utilizzato l'errore standard asintotico presumendo l'ipotesi nulla.	
c. Basato sull'approssimazione normale.	

CROSSTABS
  /TABLES=Etàgruppo BY Esitodopo
  /FORMAT=AVALUE TABLES
  /STATISTICS=CHISQ CORR
  /CELLS=COUNT
  /COUNT ROUND CELL.


Tabelle di contingenza


Note	
Output creato	01-DEC-2020 10:36:12	
Commenti		
Input	Dataset attivo	Dataset1	
	Filtro	<nessuno>	
	Peso	<nessuno>	
	Suddividi file	<nessuno>	
	N di righe nel file di dati di lavoro	54	
Gestione valori mancanti	Definizione di mancante	I valori mancanti definiti dall'utente vengono trattati come mancanti.	
	Casi utilizzati	Le statistiche per ciascuna tabella sono basate su tutti i casi con dati validi nell'intervallo o negli intervalli specificati per tutte le variabili in ciascuna tabella.	
Sintassi	CROSSTABS
  /TABLES=Etàgruppo BY Esitodopo
  /FORMAT=AVALUE TABLES
  /STATISTICS=CHISQ CORR
  /CELLS=COUNT
  /COUNT ROUND CELL.	
Risorse	Tempo processore	00:00:00,00	
	Tempo trascorso	00:00:00,00	
	Dimensioni richieste	2	
	Celle disponibili	524245	


Riepilogo elaborazione casi	
	Casi	
	Valido	Mancante	Totale	
	N	Percentuale	N	Percentuale	N	
Età gruppo * Esito dopo	54	100,0%	0	0,0%	54	

Riepilogo elaborazione casi	
	Casi	
	Totale	
	Percentuale	
Età gruppo * Esito dopo	100,0%	


Tavola di contingenza Età gruppo * Esito dopo	
Conteggio  	
	Esito dopo	Totale	
	1	2		
Età gruppo	0	0	1	1	
	1	2	7	9	
	2	2	8	10	
	3	2	4	6	
	4	3	2	5	
	5	4	9	13	
	6	6	3	9	
	7	0	1	1	
Totale	19	35	54	


Test del chi-quadrato	
	Valore	df	Significatività asintotica (bilaterale)	
Chi-quadrato di Pearson	8,142a	7	,320	
Rapporto di verosimiglianza	8,631	7	,280	
Associazione lineare per lineare	3,291	1	,070	
N di casi validi	54			

a. 12 celle (75,0%) hanno un conteggio previsto inferiore a 5. Il conteggio previsto minimo è ,35.	


Misure simmetriche	
	Valore	Errore standard asintoticoa	T approssimatob	
Intervallo per intervallo	R di Pearson	-,249	,127	-1,856	
Ordinale per ordinale	Correlazione di Spearman	-,254	,128	-1,894	
N di casi validi	54			

Misure simmetriche	
	Significatività approssimata	
Intervallo per intervallo	R di Pearson	,069c	
Ordinale per ordinale	Correlazione di Spearman	,064c	
N di casi validi		

a. Non viene assunta l'ipotesi nulla.	
b. Viene utilizzato l'errore standard asintotico presumendo l'ipotesi nulla.	
c. Basato sull'approssimazione normale.	
